# Characteristics and Functions of MYB (v-Myb avivan myoblastsis virus oncogene homolog)-Related Genes in *Arabidopsis thaliana*

**DOI:** 10.3390/genes14112026

**Published:** 2023-10-31

**Authors:** Guofan Wu, Aohua Cao, Yuhan Wen, Wencheng Bao, Fawen She, Wangze Wu, Sheng Zheng, Ning Yang

**Affiliations:** College of Life Sciences, Northwest Normal University, Lanzhou 730070, China; 2021212786@nwnu.edu.cn (A.C.); 18845489920@163.com (Y.W.); 18093281592@163.com (W.B.); 2021212794@nwnu.edu.cn (F.S.); wangzew78@sina.cn (W.W.); zhengsheng@nwnu.edu.cn (S.Z.); xbsd-yn@163.com (N.Y.)

**Keywords:** *Arabidopsis thaliana*, MYB-related, bioinformatics analysis, gene functions

## Abstract

The MYB (*v-Myb* avivan myoblastsis virus oncogene homolog) transcription factor family is one of the largest families of plant transcription factors which plays a vital role in many aspects of plant growth and development. MYB-related is a subclass of the MYB family. Fifty-nine *Arabidopsis thaliana* MYB-related (*AtMYB*-related) genes have been identified. In order to understand the functions of these genes, in this review, the promoters of *AtMYB*-related genes were analyzed by means of bioinformatics, and the progress of research into the functions of these genes has been described. The main functions of these *AtMYB*-related genes are light response and circadian rhythm regulation, root hair and trichome development, telomere DNA binding, and hormone response. From an analysis of cis-acting elements, it was found that the promoters of these genes contained light-responsive elements and plant hormone response elements. Most genes contained elements related to drought, low temperature, and defense and stress responses. These analyses suggest that *AtMYB*-related genes may be involved in *A. thaliana* growth and development, and environmental adaptation through plant hormone pathways. However, the functions of many genes do not occur independently but instead interact with each other through different pathways. In the future, the study of the role of the gene in different pathways will be conducive to a comprehensive understanding of the function of the gene. Therefore, gene cloning and protein functional analyses can be subsequently used to understand the regulatory mechanisms of *AtMYB*-related genes in the interaction of multiple signal pathways. This review provides theoretical guidance for the follow-up study of plant MYB-related genes.

## 1. Introduction

The regulation of gene expression controls many important biological processes. This regulatory pathway is often complex and diverse, requiring the involvement of multiple factors, including transcription factors, which have been studied in considerable detail. Transcription factors can recognize specific DNA motifs in gene regulatory regions and, when combined with specific sites on the target gene promoter, regulate transcription [1,2]. The MYB (*v-Myb* avivan myoblastsis virus oncogene homolog) transcription factors are one of the largest families of transcriptional regulators in plants; they are involved in growth and development, secondary metabolism, signal transduction, and biotic and abiotic stresses [3]. There has been much research and interest in the R2R3-MYB gene, likely due to the wide distribution of R2R3-MYB in plants. However, little attention has been paid to the analysis and systematic induction of *AtMYB*-related genes. This review analyzes the structure and promoter characteristics of the 59 *Arabidopsis* MYB-related genes recently identified by Lal et al. [4], and summarizes the functions of these genes. This work will help direct follow-up experiments to verify the functions of these genes in growth, development, and stress responses, and will provide a theoretical basis for the study of MYB-related genes.

## 2. Survey Methodology

Based on the *AtMYB*-related genes’ IDs reported by Lal et al. [4], we searched the literature for information on *AtMYB*-related genes in TAIR (https://www.arabidopsis.org/, accessed on 20 January 2023) and NCBI PubMed [4]. We performed a content review and analysis of the literature, both classic and published within the last 5 years.

The genome sequence file and General Feature Format Version 3 (gff3) file for *Arabidopsis thaliana* were downloaded from Ensembl Plants (http://plants.ensembl.org/index.html, accessed on 20 January 2023). Sequences of *AtMYB*-related proteins were extracted using TBtools [5]. The MAFFT version 7 website (https://mafft.cbrc.jp/alignment/server/, accessed on 20 January 2023) was used with default parameters to perform multiple sequence alignment on the *AtMYB*-related protein sequences [6,7]. Then, a neighbor-joining (NJ) phylogenetic tree was constructed using MAGE version 11 with the following parameters: p-distance, pairwise deletion, and bootstrap analysis with 1000 replicates [8,9]. The conserved motifs (Supplementary Figure S1, Supplementary Table S1) of *AtMYB*-related genes were predicted by using MEME (https://meme-suite.org/meme/tools/meme, accessed on 21 January 2023) with the following parameters: the distribution of motifs—0 or 1 per sequence; maximum number of motifs to find—8; and other parameters—default values [10]. The intron/exon structure information was obtained from the gff3 file. The sequences of the 2000 base pairs upstream of the *AtMYB*-related genes were extracted using TBtools. The PlantCARE database (http://bioinformatics.psb.ugent.be/webtools/plantcare/html/, accessed on 21 January 2023) was used to predict and analyze the cis-acting elements of the sequence 2000 bp upstream of each *AtMYB*-related gene [11]. The promoter sequences of *AtMYB*-related genes are shown in Supplementary File S1. Finally, TBtools was used for visualization.

## 3. Identification and Characterization of the MYB Genes

The transcription factor MYB was first found in the avian acute myeloblastic leukemia virus in 1941, and in 1982 it was identified and named *v-myb* [12,13]. In 1987, the first plant MYB transcription factor *ZmMYBC1* was identified in maize. Its function is related to anthocyanin synthesis [14]. In 1989, the first fungal MYB transcription factor *Bas1* was identified in *Saccharomyces cerevisiae*, which was found to be necessary to activate the transcription of the histidine dehydrogenase gene *HIS4* [15]. The characterization and classification of a gene family is the first step in functional research. Subsequently, increasing numbers of members of the MYB gene family have been identified, and the functions of more MYB genes have been discovered. Chen et al. [16] identified a total of 198 MYB genes in *A. thaliana*, of which 64 were MYB-related genes. From the phylogenetic analysis, 60 MYB-related genes were divided into five categories: CCA1-like, CPC-like, I-box-like, TBP-like, and R-R type [16]. Subsequently, Katiyar et al. identified 197 MYB genes in *A. thaliana*, including 52 MYB-related genes [17]. Recently, Lal et al. identified 193 MYB genes in *A. thaliana*, including 59 MYB-related genes, in a comprehensive analysis of 1R- and 2R-MYBs [4]. There are a total of 122 MYBs in *Brachypodium distachyon* [18] compared with 171 MYBs in *Chinese jujube* [19]. Arce-Rodriguez et al. identified a total of 235 MYBs in chili pepper and proposed some candidate genes that may be involved in the regulation of phenylpropane, capsaicin, carotenoid, and vitamin C biosynthesis [20]. In *Morus alba*, a total of 166 MYBs were identified [21]. A number of identifications and studies have also been published on MYB-related genes in other plants, including soybean [22], potato [23], *Brassica napus* L. [24], populus [9], pepper [25], and sweet osmanthus [26].

## 4. Structures and Main Functions of the MYB Gene Family

**Figure 1:**
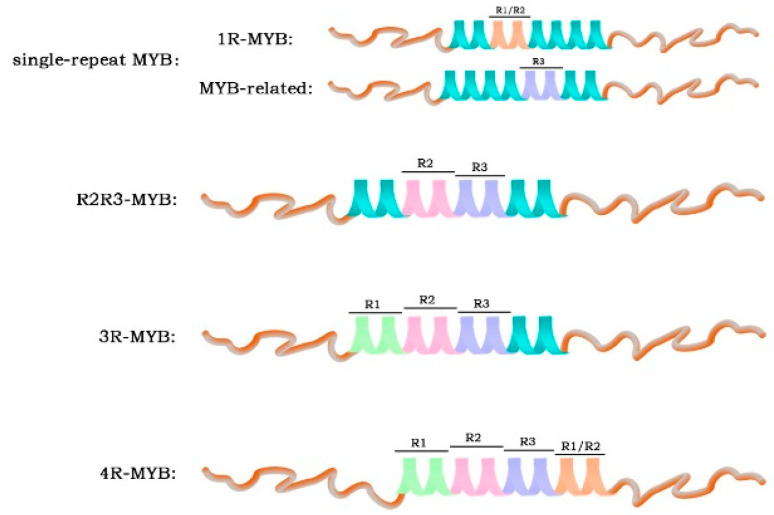
Classification of MYB transcription factors in plants.

The N-terminus of the MYB transcription factor contains a conserved specific DNA-binding domain (MYB domain) [27]. This domain generally consists of one to four sequence repeats (R) of about 52 amino acids, each of which forms three α helices. The second and third α helices of each repeat form a helix–angle–helix (HTH) structure [2,27]. Based on the number of domains, MYB can be divided into four categories (Figure 1): a single-repeated MYB with one R sequence, called 1R-MYB or MYB-related; R2R3-MYB with two R sequences; 3R-MYB with three R sequences; and 4R-MYB with four R sequences [27]. 

1R-MYB, which contains complete or partially repeated proteins, is the second largest subclass of the MYB family and is widely distributed in plants [27]. Phylogenetic and expression analyses revealed the conservation and diversity of MYB-related genes, and functional studies showed that they regulate plant development and stress response [28]. The R2R3-MYB subfamily is the largest group in the plant MYB family. It may have evolved from a 3R-MYB that lost the R1 sequence or from a 1R-MYB that evolved from the copying of the sequence [29,30]. The abundance of 3R-MYB is relatively low in plants. Recent studies have shown that its main function is related to the cell cycle and protein regulation, as well as cell differentiation [29,31]. The smallest member of the MYB family is 4R-MYB. Its function is still unclear and is under investigation [32]. 

In recent years, with the identification and functional studies of the MYB genes, the number of reviews about the MYB genes has increased. These reviews describe two main points: (1) the biochemical and molecular characteristics of MYB transcription factors [33], including their type, structure, evolution, and function [1,27]; and (2) the role of MYB transcription factors in controlling various biological processes, including stress responses [34,35] and secondary metabolic processes [36]. For example, regarding advances in research on MYB transcription factors in plant stress resistance and breeding [37], Yan et al. discussed the regulatory mechanism of the MYB transcription factors in anthocyanin biosynthesis [32]. Recently, progress has been reported on MYB transcription factors regulating multiple functions in medicinal plants [38]. In addition, studies based on the genome-wide analysis of the structure and evolution of MYB genes have also been published [28]. 

## 5. Functions of MYB-Related Genes

Although MYB-related genes contain only one or part of the MYB/SANT domain, this domain is still necessary for the function of MYB transcription factors. On the one hand, it plays an important role in overcoming abiotic and biotic stresses by regulating various defense mechanisms in many plants [39]. For example, the MYB-related transcription factor *TaLHY* plays an important role in resistance to stripe rust in wheat [40]. On the other hand, some MYB-related genes participate in some biological pathways and regulate other related genes. For example, *ZmMYB48*, *OsMYB48-1*, and *StMYB1R-1* confer drought resistance by regulating the expression of stress response marker genes and controlling physiological functions [41,42,43]. The wheat MYB-related transcription factor *TaMYB72* promotes rice flowering by upregulating the Florigen genes *HD3A* and *RFT1* [44]; *GmMYB118* improves drought and salt tolerance by inducing the expression of stress-related genes and regulating osmotic and oxidizing substances [22]; an MYB-related transcription factor from *Lilium lancifolium* L. (*LlMYB3*) participates in the *Arabidopsis* anthocyanin biosynthesis pathway and enhances the tolerance of *A. thaliana* to a variety of abiotic stresses [45]; and the *OsMYB-R1* gene regulates resistance to multiple stressors in rice through the interaction between auxin and salicylic acid [46].

Supplementary Table S2 presents information on *AtMYB*-related genes. The functions of *AtMYB*-related genes are described in detail below.

## 6. Light Response and Circadian Rhythm Regulation

The large number of circadian rhythm regulation genes in *Arabidopsis* reflects the important role of the circadian rhythm in plant growth and development. This is consistent with the fact that the expression of many genes, including those involved in photosynthesis and light signaling, oscillates rhythmically. In addition, many physiological processes are controlled by the circadian rhythm, such as flowering and the movement of cotyledons and leaves [47].

CIRCADIAN CLOCK ASSOCIATED 1 (CCA1) and LATE ELONGATED HYPOCOTYL (LHY) are MYB-related proteins that play a role in or close to the central oscillator of *Arabidopsis,* and have a synergistic effect in regulating the circadian rhythm of *Arabidopsis* [48]. The MYB-related protein encoded by the *CCA1* gene binds to a region of the light-harvesting chlorophyll a/b protein gene (Lhcb1*3) promoter, which can affect the regulation of the phytochrome promoter in vivo. The expression of the *CCA1* gene itself can also be regulated by light, leading to the increased transcription of the Lhcbl*3 gene, which is considered to be part of the phytochrome signal transduction chain [49]. A vernalization-responsive cis-element (VREVIN3) was identified in the *VERNALIZATION INSENSITIVE 3 (VIN3)* promoter, which consists of two known continuous cis elements: a G-box and an evening element (EE). Kyung et al. found that *CCA1* and *LHY* are involved in the transcriptional activation of *VIN3* by binding to EE. In addition, the rhythmic expression patterns of *CCA1* and *LHY* also changed after long-term cold exposure. Therefore, it is considered that *CCA1* and *LHY* are part of the signal transduction mechanism to ensure the vernalization of *Arabidopsis* [50]. *CCA1* and *LHY* also promote the expression of two day-phased genes, *PRR7* and *PRR9*, which in turn are suppressed by these PRRs and their homolog *PRR5*, forming another negative feedback circuit [51,52]. *EARLY-PHYTOCHROME-RESPONSIVE1 (EPR1)* leads to the enhanced opening and delayed flowering of cotyledons induced by far-red light. In wild *Arabidopsis* plants growing under continuous light, *EPR1* shows a similar circadian rhythm to *CCA1* and *LHY*. In addition, *EPR1* can adjust its expression to form a slave oscillator, which also adjusts the rhythm of *Lhcb* [53].

The expression of *CIRCADIAN1 (CIR1/RVE2)* is transiently induced by light and oscillates in a circadian rhythm controlled by the central oscillator *CCA1*. The constitutive expression of *CIR1 (RVE2)* inhibits the rhythmic expression of the endogenous *CIR1 (RVE2)* gene, changes the cycle length of the central oscillator, reduces the amplitude of *CCA1* and *LHY*, and seriously affects the rhythm of the *EPR1* and *Lhcb* genes. In addition, the overexpression of *CIR1 (RVE2)* delayed photoperiod flowering, decreased the expression of *CON-STANS (CO)* and *FLOWERING LOCUS T (FT)*, increased hypocotyl elongation, and inhibited seed germination in the dark [54]. In addition, plants overexpressing *CIR1 (RVE2)* showed an increased expression of the *CBF* gene and enhanced tolerance to freezing stress before and after cold acclimation. This indicates that *CIR1 (RVE2)* positively regulates cold response genes and subsequent cold tolerance [55]. *RVE2* and *REVEILLE1 (RVE1)* can promote the primary dormancy of *Arabidopsis* seeds and inhibit the red/far-red light-mediated germination downstream of phytochrome B (phyB) [56]. *RVE1* was first proved to be a clock-regulated transcription factor that promotes the expression of the auxin biosynthesis gene *YUCCA8 (YUC8)* and is essential for the circadian rhythm of auxin. Therefore, *RVE1* can promote the accumulation of free auxin and the elongation of hypocotyl of seedlings in the daytime [57]. In another study, two independent knockout mutants of *RVE1* against the background of Col showed enhanced freezing resistance under domestication, which proved that *RVE1* was a negative regulator of freezing resistance in *Arabidopsis* [58]. Recently, it has been found that *RVE1* positively regulates the transcription of *PORA* (protochlorophyllide oxidoreductase A) by directly binding to the EE-box cis-regulatory element of the *PORA* promoter. *PORA* can catalyze the reduction of protochlorophyllide to chlorophyll. The analysis of *PORA* expression in loss-of-function and overexpressing *RVE1 Arabidopsis* plants showed that *RVE1* regulated the transcription of *PORA* and promoted seedling greening [59].

Many metabolic, physiological, and behavioral processes in plants are controlled by the biological clock [60,61], for instance, the response of plants to auxin. Recently, it has been reported that *CCA1* and *LHY* are necessary for the control of the auxin response [62]. *LHY* has also been found to be involved in the regulation of the ABA pathway. The genome-wide analysis of its binding targets has shown that *LHY* can bind to the promoters of many ABA biosynthesis and signal transduction genes. Under drought stress, plants with *LHY* overexpression accumulated lower levels of ABA, and *LHY* mutants were more sensitive to ABA treatment during seed germination [63]. Similarly, the biosynthesis and signal genes of most brassinosteroids (BRs) are also controlled by clocks [64]. The BR-activated transcription factor bri1-EMS-suppressor 1 (BES1) regulates the expression of *CCA1* and *LHY* and then transmits the BR signal to the clock oscillator. In the presence of BRs, BES1 binds the *CCA1* and *LHY* promoters and inhibits their expression, especially at night. This BES1-CCA1/LHY module subtly adjusts the circadian rhythm oscillation to ensure that the BR signal is activated acutely at a specific time of the day [65]. Lei and Zhu-Salzman reported that both *CCA1* and *LHY* are necessary for the circadian regulation of indole glucosinolate biosynthesis, and contribute to plant defense against aphids [66]. Furthermore, *CCA1* and *LHY* inhibited the expression of the dehydration-responsive element (DRE) binding protein 1 *(DREB1)* under non-stress and were degraded rapidly and specifically under cold stress. Therefore, as transcriptional suppressors, *CCA1* and *LHY* can indirectly regulate the cold-induced expression of *DREB1* [67].

The functional deletion mutation of *REVEILLE5 (RVE5)* decreased the expression of the circadian gene *EARLY FLOWERING 4 (ELF4)* in *Arabidopsis,* and promoted the growth of hypocotyls under warm conditions [68]. *REVEILLE8 (RVE8)* can bind directly to the promoter of *TIMING OF CAB EXPRESSION1 (TOC1)* and promote the expression of histone H3 by increasing the acetylation level of histone H3, while *CCA1* inhibits the expression of *TOC1* by reducing the level of histone acetylation [69]. RVE8 can directly activate the clock and output genes containing EE. The loss of *RVE8* and its homologs, *RVE4* and *RVE6*, causes a delay and reduction in levels of evening-phased clock gene transcripts and a significant lengthening of the clock pace [70]. In addition, Perez-Garcia et al. showed that *RVE8* could directly bind to the promoters of anthocyanin-biosynthesis-related genes and regulate the expression of these genes in response to diurnal fluctuations. *NIGHT LIGHT-INDUCIBLE AND CLOCK-REGULATED (LNK)* and *RVE8* can collaboratively control the anthocyanin metabolic pathway [71]. Based on these findings, *RVE8* can be considered a regulator of anthocyanin biosynthesis in plants [72]. In another study [73], *RVE3* and *RVE5* promoted only the clock speed together with *RVE4*, *RVE6*, and *RVE8*, and their roles in the clock function were subtle. However, *RVE3* and *RVE5* only played a secondary role in the adjustment of the clock function [73].

Light morphogenesis in plants is often regulated by a variety of hormone signal pathways. The *MYBS3* homologous gene in *Arabidopsis*, *MYBH*, was induced in the dark, which enhanced the expression of auxin-related genes, such as *PIF4* and *PIF5*, and then induced the accumulation of auxin, thus increasing the elongation of the hypocotyl. At the same time, it was found that the transcription level of the auxin biosynthesis gene *YUCCA8 (YUC8)* was also increased in *MYBH*-overexpressing seedlings [74]. Additionally, researchers [75] have observed that, in transgenic *Arabidopsis*, the overexpression of *MYBH* enhanced the gene expression of *SAUR36*, a key regulator of auxin-induced leaf senescence, and accelerated leaf senescence induced by ABA and ethylene. In this study, it was also found that darkness and aging could activate the activity of the *MYBH* promoter [75]. The biosynthesis of anthocyanins is often regulated by many factors, such as light, auxin, and cytokinin. Light and cytokinin can stimulate the expression of *MYBD*, a homologous gene of *MYBH*. *MYBD* inhibits the expression of *MYBL2* and promotes the biosynthesis of anthocyanin by directly binding to the *MYBL2* promoter. It was further observed that *ELONGATED HYPOCOTYL 5 (HY5)* directly binds to the *MYBD* promoter, especially in the G-box-containing region, resulting in anthocyanin accumulation. It can be seen that *MYBD* and *MYBH* have opposing roles in the process of plant photomorphogenesis [76]. The expression of the *MYBL2* gene is not only inhibited by *MYBD* but also by *HY5*. Furthermore, the translation of *MYBL2* is suppressed by microRNA MIR858a. MIR858a is the direct target of *HY5* and displays light-responsive expression in an HY5-dependent manner [77]. Thus, AtHY5-AtMYBD-AtMYBL2 and AtHY5-miR858a-AtMYBL2 work together to form a control system for anthocyanin regulation [36]. Recently, *AtGLK1 (GOLDEN2-LIKE 1)* was reported to regulate sucrose-induced anthocyanin synthesis upstream of *MYBL2* [78].

## 7. The Development of Trichomes and Root Hairs

*CAPRICE (CPC)* was first reported to be involved in the development of trichomes and root hairs [79]. Studies have shown that *CPC* reduces the formation of the TRANSPARENT TESTA GLABRA1 (TTG1)-(E) GL3-GL1 complex by competing with R2R3-MYB *GL1* for bHLH binding at the initiation of trichomes, thus inhibiting trichome formation [80]. Then, it was found that *CPC* is a positive regulator of stomatal formation [81]. In addition, in the overexpression of *CPC* in plants, the accumulation of anthocyanin is negatively correlated with the level of *CPC.* It was also found that the regulation of anthocyanin biosynthesis in *Arabidopsis* is inhibited by competition with R2R3-MYB for the binding site of the bHLH protein, which prevents R2R3-MYB and bHLHs from forming an active complex of anthocyanin biosynthesis, thus negatively controlling anthocyanin biosynthesis [82]. According to previous studies, *CPC* and its six homologs—*TRIPTYCHON (TRY)*, *ENHANCER OF TRY AND CPC1 (ETC1)*, *ENHANCER OF TRY AND CPC2 (ETC2)*, *ENHANCER OF TRY AND CPC3 (ETC3)/CAPRICELIKE MYB3 (CPL3)*, *TRICHOMELESS1 (TCL1),* and *TRICHOMELESS2 (TCL2)*—can induce root hair differentiation and inhibit trichome formation [83,84,85,86,87,88,89,90,91]. These *CPC* family genes lead to root hair formation mainly by inhibiting the expression of *GLABRA2 (GL2)* [92]. The overexpression of the *MYBL2* gene inhibits root hair development in transgenic *Arabidopsis*. The synergistic effect of *GL3* gene function and the overexpression of *MYBL2* inhibit hair formation by negatively regulating the expression of *GLABRA2 (GL2)* [93]. Among the homologs, only *CPL3* has multiple effects on flower development and epidermal cell size by regulating internal replication [86]. It was reported that, under the condition of phosphate (Pi) deficiency, the expression of the *ETC1* and *ETC3* genes is enhanced, which can promote root hair formation by inhibiting the expression of *GL2* [94]. Another study found that the rice gene *OsTCL1* could inhibit the formation of trichomes and promote the formation of root hairs when expressed in *Arabidopsis*. However, the expression of *OsTCL1* in rice had no effect on the formation of root hairs and trichomes, and the expression of the *OsGL2* gene was increased rather than decreased. This shows that rice regulates the formation of trichomes and root hairs differently from *Arabidopsis* [95]. Recently, it has been reported that different climates and genomic structures lead to trichome diversity [96].

## 8. Telomere Metabolism

In *Arabidopsis*, *AtTRB1-5* is a plant-DNA-binding protein [97,98]. The C-terminus of *AtTBP1* can bind sequence-specific DNA with plant double-stranded telomere DNA, which may play an important role in plant telomere function in vivo [99]. *AtTBP2 (TRB3)* and *AtTBP3 (AtTRB2)* have MYB-like domains at the N-terminus. They do not affect telomerase activity in vitro, but are similar to other MYB-like telomere-binding proteins and can indirectly participate in the regulation of telomere metabolism [100]. The TRFL family 1 proteins have a highly conserved region in the C-terminus of the MYB domain, called MYB expansion (MYB-ext), which is necessary for binding to plant telomere DNA and does not exist in the TRFL family 2. The TRFL family 1 includes *TBP1, TRP1, TRFL1, TRFL2, TRFL4,* and *TRFL9*, while the TRFL family 2 contains *TRFL3, TRFL5-8*, and *TRFL10*. In vitro, the C-terminal fragment of the TRFL family 1 protein binds specifically to the double-stranded plant telomere DNA. TRFL family 2 proteins cannot bind to plant telomere DNA in vitro. TRFL family 2 proteins may bind to telomeres in vivo through protein interactions, similar to human *Rap1*. The TRFL family 1 protein is not the only factor that can specifically bind to double-stranded plant telomere DNA in vitro [101]. Telomere binding proteins are not only considered to be essential components of the telomere structure but are also important components of the telomere metabolism involved in telomere length regulation and telomere protection [102]. For instance, a double-stranded telomeric repeat binding factor in *Nicotiana tabacum*, *NgTRF1*, is involved in the maintenance of telomere length and stability [103]. In another study, *RICE TELOMERE BINDING PROTEIN1 (RTBP1)* was also involved in controlling telomere length and telomere stability in rice [104]. We found that previous studies of these telomere-binding proteins in plants focused mainly on their interactions in vitro, and the physiological role of these proteins in plants is still unclear.

## 9. Plant Hormone Response

The RAD-like family in *Arabidopsis* consists of at least four members: *RADILAS-LIKE SANT/MYB1-4 (RSM1, RSM2, RSM3*, and *RSM4)*. *RSM1 (RL2)* is closely related to the *HLS1* gene in early morphogenesis [105]. *HLS1* was originally identified as an important regulator implicated in the formation and maintenance of the apical hook of dark-grown etiolated seedlings in response to ethylene [106,107]. In addition, *RSM1* and *HY5/HYH* may converge on the *ABI5* promoter and independently (or possibly, dependently) regulate *ABI5* expression and ABI5-targeted ABA-responsive genes, thereby modulating ABA and abiotic stress responses [108]. At present, it is known that these four genes in the RAD-like family are highly homologous to the *Antirrhinum RAD* genes, although the functions of *RSM2, RSM3,* and *RSM4* in *Arabidopsis* have not been studied [105]. *DRMY1* controls cell expansion in vegetative and reproductive organs and is strongly expressed in developing organs. Its expression is inhibited by ethylene and induced by ABA. *DRMY1* plays an important role in organ development by directly affecting the cell wall structure and cytoplasmic growth, or by indirectly regulating cell expansion through ethylene or ABA signaling pathways [109]. *DRMY1* focuses the spatiotemporal signaling patterns of the plant hormones auxin and cytokinin, which jointly control the timing of sepal initiation. *DRMY1* ensures sepal size uniformity by coordinating the timing of sepal initiation [110]. As a transcription factor, *NID1* binds directly to the *CHL1* promoter under a low-nitrate condition, activates an unknown pathway through the *CHL1* receptor, and promotes ABA accumulation, thereby inhibiting root growth [111]. 

## 10. Promoter Analysis of *AtMYB*-Related Genes

**Figure 2:**
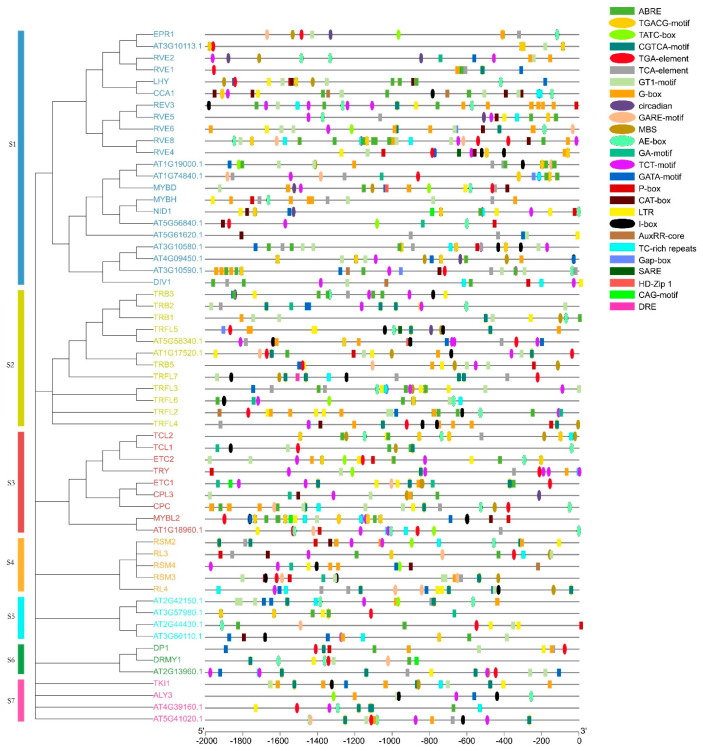
Cis-elements in the promoter regions of *AtMYB*-related genes. The promoter sequences of *AtMYB*-related genes (−2000 bp) were analyzed by using PlantCARE. The upstream lengths of the translation start sites can be estimated using the scale at the bottom.

In order to gain a comprehensive and in-depth understanding of the functions of MYB-related genes during plant development, we divided these genes into seven subgroups according to the phylogenetic tree (Supplementary Figure S2) and predicted the promoter sequences of *AtMYB*-related genes (Figure 2, Supplementary Table S3). Except for the core promoter element and common cis-acting element in the promoter (CAAT-box and TATA box), the 26 cis-elements detected could be divided into three types: growth and development response, hormone response, and stress response. Among the response elements related to plant growth and development, the number of light-responsive elements was the largest, including TCT-motif, G-box, GT1-motif, and AE-box. The distribution of light-responsive elements was also the widest. All *AtMYB*-related genes contained light-responsive elements, indicating that *AtMYB*-related genes played a role in light-response-mediated regulation. In addition, cis-acting elements involved in circadian rhythm control were detected in nine genes. Of course, this response element was not detected in many genes, but this does not mean that they do not play a role in the regulation of circadian rhythm because they may play a role through indirect effects with other related genes, such as *CCA1*. CAT-box and HD-Zip1 are related to gene expression in the meristem and the differentiation of palisade mesophyll cells, respectively, and may be related to specific expression. Ten cis-acting elements are involved in the reactions of abscisic acid (ABA), auxin, gibberellin (GA), salicylic acid (SA), and jasmonic acid (JA), including ABRE, TGA-element, GARE-motif, TCA-element, and the TGACG-motif. Stress response elements include MBS and DRE, which are related to drought and low temperature. There were 30 drought-responsive elements (MBS) in 23 *AtMYB*-related genes, while DRE was predicted only in *TRFL7*. The genes containing these cis-elements in the promoter region may be involved in the adaptation of *Arabidopsis* to different environmental conditions.

## 11. Discussion

The analysis of cis-acting elements of *AtMYB*-related gene promoters and the review of previous functional studies can not only help us better understand the functions these genes already possess, but can also provide a direction for future research on the functions of these genes. We found that genes belonging to the same subgroup have similar structures and functions. For example, genes with known functions in the S1 subgroup are involved in the regulation of light response or circadian rhythm. Therefore, it is speculated that those genes with unknown functions in the S1 subgroup may also be involved in the regulation of related functions. In addition, the activity of the *RVE1* promoter is regulated by the clock [57]; the expression of *RVE2* can be rapidly induced by light [54]; and the activity of the *MYBH* promoter is regulated by light [74]. All of these findings are consistent with the fact that their promoter regions contain light-responsive elements. It is not difficult to see that *CCA1* and *LHY* are involved in many biological processes, complex pathways, and interactions with many other transcription factors. For example, *ATAF2 (ANAC081)* is an NAC (NAM, ATAF, and CUC) transcription factor (TF). *CCA1* physically interacts with *ATAF2* and inhibits *ATAF2* expression through promoters that bind to CBS motifs. *CCA1* and *ATAF2* were reported to synergistically inhibit the light morphogenesis of seedlings [112]. Genes belonging to the S3 subgroup are all involved in the regulation of trichome or root hair development. *TRB1, TRB2, TRB3*, and the genes of TRFL subsets belonging to the S2 subgroup all bind to telomere DNA, but their physiological roles and developmental regulation are still unclear. The function of *DRMY1* in the S6 group is to regulate cell expansion, but the function of its paralog gene, *DP1,* is still unknown. RAD-like genes in the S4 subgroup are related to the growth and development of *Arabidopsis*, but their specific function is not clear. In particular, although *NID1* belongs to the S1 subgroup and its promoter contains cis-acting elements for circadian control, it has not been reported that it is involved in the regulation of circadian rhythm, so further study is needed. Therefore, the known functions of these genes partly verify the results of the promoter analysis, but the functions of these genes are often diverse. For example, *CPC* not only participates in the development of trichomes and root hairs but also negatively regulates anthocyanin accumulation and positively regulates stomatal formation. The same is true of MYB-related genes in other plants. For example, the overexpression of the rice *MYB-R1* gene can increase tolerance to drought, salt, and chromium stresses simultaneously [113].

Of course, in recent years, there have also been reports that *AtMYB*-related genes are involved in both biological clock and abiotic stress responses. For example, the genome-wide analysis of *LHY* binding sites shows that *LHY* directly controls the expression of genes related to the biosynthesis of ABA and the rhythmic accumulation of this hormone. Furthermore, *LHY* also regulates the expression of ABA signal modules and downstream response genes to enhance some ABA responses and inhibit others. This reveals the complex coupling between the biological clock and the ABA pathway, which may make an important contribution to plant performance under drought and osmotic stress [63]. Therefore, these genes not only play a role in one pathway, but also interact with each other in multiple signaling pathways. 

## 12. Conclusions and Outlook

This review summarized the advancement in research on the functions of MYB-related genes in *A. thaliana* and analyzed the promoter characteristics of *AtMYB*-related genes using bioinformatics. However, the functional studies of most reported *AtMYB*-related genes are relatively simple, and the research on their regulatory mechanisms in the interaction of different signal pathways is not sufficiently extensive. Moreover, the functions of some *AtMYB*-related genes, such as *TKI1, ALY3,* and *DIV1*, are still unknown. There are even some unnamed *AtMYB*-related genes with functions that have not yet been separately reported. Moreover, there are few studies on these gene promoters. On the one hand, it is important to identify the upstream regulators that interact with the promoters of these genes. On the other hand, as transcription factors, it is crucial to identify the downstream target genes to which they bind. Therefore, there is still a long way to go to map complete complex pathways. First, the unidentified MYB-related transcription factors’ biological roles can be anticipated by studying their protein structures and promoter cis-acting elements. Then, we can use gene cloning and protein functional analysis to study their mechanisms in growth, development, and stress responses. Second, a study of the regulatory mechanism of *AtMYB*-related genes in the interactions of multiple signal pathways is also very important for understanding their function. Finally, these studies can be applied to the development and utilization of related genes in other plants, especially crops, to improve their yield through crop breeding.

## Data Availability

All data supporting the findings of this study are available within the paper and its Supplementary Information.

## Supplementary Materials

The following supporting information can be downloaded at: https://www.mdpi.com/article/10.3390/genes14112026/s1, File S1: The promoter sequences of *AtMYB*-related genes; Figure S1: The logo of the conserved motifs in *AtMYB*-related genes; Figure S2: Phylogenetic tree, motif and gene structure of *AtMYB*-related genes; Table S1: The sequence of the conserved motifs in *AtMYB*-related genes; Table S2: The overall information of *AtMYB*-related genes; Table S3: Cis-elements analysis of the *AtMYB*-related genes

## References

[1] Feller A., Machemer K., Braun E.L., Grotewold E. (2011). Evolutionary and comparative analysis of MYB and bHLH plant transcription factors. Plant J..

[2] Stracke R., Werber M., Weisshaar B. (2001). The R2R3-MYB gene family in *Arabidopsis thaliana*. Curr. Opin. Plant Biol..

[3] Cao Y., Li K., Li Y., Zhao X., Wang L. (2020). MYB Transcription Factors as Regulators of Secondary Metabolism in Plants. Biology.

[4] Lal M., Bhardwaj E., Chahar N., Yadav S., Das S. (2022). Comprehensive analysis of 1R- and 2R-MYBs reveals novel genic and protein features, complex organisation, selective expansion and insights into evolutionary tendencies. Funct. Integr. Genom..

[5] Chen C., Chen H., Zhang Y., Thomas H.R., Frank M.H., He Y., Xia R. (2020). TBtools: An Integrative Toolkit Developed for Interactive Analyses of Big Biological Data. Mol. Plant.

[6] Kuraku S., Zmasek C.M., Nishimura O., Katoh K. (2013). aLeaves facilitates on-demand exploration of metazoan gene family trees on MAFFT sequence alignment server with enhanced interactivity. Nucleic Acids Res..

[7] Katoh K., Rozewicki J., Yamada K.D. (2019). MAFFT online service: Multiple sequence alignment, interactive sequence choice and visualization. Brief. Bioinform..

[8] Tamura K., Stecher G., Kumar S. (2021). MEGA11: Molecular Evolutionary Genetics Analysis Version 11. Mol. Biol. Evol..

[9] Yang X., Guo T., Li J., Chen Z., Guo B., An X. (2021). Genome-wide analysis of the MYB-related transcription factor family and associated responses to abiotic stressors in Populus. Int. J. Biol. Macromol..

[10] Bailey T.L., Boden M., Buske F.A., Frith M., Grant C.E., Clementi L., Ren J., Li W.W., Noble W.S. (2009). MEME SUITE: Tools for motif discovery and searching. Nucleic Acids Res..

[11] Lescot M., Dehais P., Thijs G., Marchal K., Moreau Y., Van de Peer Y., Rouze P., Rombauts S. (2002). PlantCARE, a database of plant cis-acting regulatory elements and a portal to tools for in silico analysis of promoter sequences. Nucleic Acids Res..

[12] Graf T. (1992). Myb: A transcriptional activator linking proliferation and differentiation in hematopoietic cells. Curr. Opin. Genet. Dev..

[13] Klempnauer K.-H., Gonda T.J., Michael Bishop J. (1982). Nucleotide sequence of the retroviral leukemia gene v-myb and its cellular progenitor c-myb: The architecture of a transduced oncogene. Cell.

[14] Paz-Ares J., Ghosal D., Wienand U., Peterson P.A., Saedler H. (1987). The regulatory c1 locus of Zea mays encodes a protein with homology to myb proto-oncogene products and with structural similarities to transcriptional activators. EMBO J..

[15] Tice-Baldwin K., Fink G.R., Arndt K.T. (1989). BAS1 has a Myb motif and activates HIS4 transcription only in combination with BAS2. Science.

[16] Chen Y., Yang X., He K., Liu M., Li J., Gao Z., Lin Z., Zhang Y., Wang X., Qiu X. (2006). The MYB transcription factor superfamily of *Arabidopsis*: Expression analysis and phylogenetic comparison with the rice MYB family. Plant Mol. Biol..

[17] Katiyar A., Smita S., Lenka S.K., Rajwanshi R., Chinnusamy V., Bansal K.C. (2012). Genome-wide classification and expression analysis of MYB transcription factor families in rice and *Arabidopsis*. BMC Genom..

[18] Chen S., Niu X., Guan Y., Li H. (2017). Genome-Wide Analysis and Expression Profiles of the MYB Genes in *Brachypodium distachyon*. Plant Cell Physiol..

[19] Qing J., Dawei W., Jun Z., Yulan X., Bingqi S., Fan Z. (2019). Genome-wide characterization and expression analyses of the MYB superfamily genes during developmental stages in *Chinese jujube*. PeerJ.

[20] Arce-Rodriguez M.L., Martinez O., Ochoa-Alejo N. (2021). Genome-Wide Identification and Analysis of the MYB Transcription Factor Gene Family in Chili Pepper (*Capsicum* spp.). Int. J. Mol. Sci..

[21] Liu L., Chao N., Yidilisi K., Kang X., Cao X. (2022). Comprehensive analysis of the MYB transcription factor gene family in *Morus alba*. BMC Plant Biol..

[22] Du Y.T., Zhao M.J., Wang C.T., Gao Y., Wang Y.X., Liu Y.W., Chen M., Chen J., Zhou Y.B., Xu Z.S. (2018). Identification and characterization of GmMYB118 responses to drought and salt stress. BMC Plant Biol..

[23] Liu Y., Zeng Y., Li Y., Liu Z., Lin-Wang K., Espley R.V., Allan A.C., Zhang J. (2020). Genomic survey and gene expression analysis of the MYB-related transcription factor superfamily in potato (*Solanum tuberosum* L.). Int. J. Biol. Macromol..

[24] Li J., Lin K., Zhang S., Wu J., Fang Y., Wang Y. (2021). Genome-Wide Analysis of Myeloblastosis-Related Genes in *Brassica napus* L. and Positive Modulation of Osmotic Tolerance by BnMRD107. Front. Plant Sci..

[25] Liu Y., Zhang Z., Fang K., Shan Q., He L., Dai X., Zou X., Liu F. (2022). Genome-Wide Analysis of the MYB-Related Transcription Factor Family in Pepper and Functional Studies of CaMYB37 Involvement in Capsaicin Biosynthesis. Int. J. Mol. Sci..

[26] Yan X., Ding W., Wu X., Wang L., Yang X., Yue Y. (2022). Insights Into the MYB-Related Transcription Factors Involved in Regulating Floral Aroma Synthesis in Sweet Osmanthus. Front. Plant Sci..

[27] Dubos C., Stracke R., Grotewold E., Weisshaar B., Martin C., Lepiniec L. (2010). MYB transcription factors in *Arabidopsis*. Trends Plant Sci..

[28] Du H., Wang Y.B., Xie Y., Liang Z., Jiang S.J., Zhang S.S., Huang Y.B., Tang Y.X. (2013). Genome-wide identification and evolutionary and expression analyses of MYB-related genes in land plants. DNA Res..

[29] Rosinski J.A., Atchley W.R. (1998). Molecular evolution of the Myb family of transcription factors: Evidence for polyphyletic origin. J. Mol. Evol..

[30] Jiang C., Gu J., Chopra S., Gu X., Peterson T. (2004). Ordered origin of the typical two- and three-repeat Myb genes. Gene.

[31] Feng G., Burleigh J.G., Braun E.L., Mei W., Barbazuk W.B. (2017). Evolution of the 3R-MYB Gene Family in Plants. Genome Biol. Evol..

[32] Yan H., Pei X., Zhang H., Li X., Zhang X., Zhao M., Chiang V.L., Sederoff R.R., Zhao X. (2021). MYB-Mediated Regulation of Anthocyanin Biosynthesis. Int. J. Mol. Sci..

[33] Du H., Zhang L., Liu L., Tang X.F., Yang W.J., Wu Y.M., Huang Y.B., Tang Y.X. (2009). Biochemical and molecular characterization of plant MYB transcription factor family. Biochemistry.

[34] Roy S. (2016). Function of MYB domain transcription factors in abiotic stress and epigenetic control of stress response in plant genome. Plant Signal. Behav..

[35] Baldoni E., Genga A., Cominelli E. (2015). Plant MYB Transcription Factors: Their Role in Drought Response Mechanisms. Int. J. Mol. Sci..

[36] Ma D., Constabel C.P. (2019). MYB Repressors as Regulators of Phenylpropanoid Metabolism in Plants. Trends Plant Sci..

[37] Li J., Han G., Sun C., Sui N. (2019). Research advances of MYB transcription factors in plant stress resistance and breeding. Plant Signal. Behav..

[38] Thakur S., Vasudev P.G. (2022). MYB transcription factors and their role in Medicinal plants. Mol. Biol. Rep..

[39] Erpen L., Devi H.S., Grosser J.W., Dutt M. (2017). Potential use of the DREB/ERF, MYB, NAC and WRKY transcription factors to improve abiotic and biotic stress in transgenic plants. Plant Cell Tissue Organ Cult. (PCTOC).

[40] Zhang Z., Chen J., Su Y., Liu H., Chen Y., Luo P., Du X., Wang D., Zhang H. (2015). TaLHY, a 1R-MYB Transcription Factor, Plays an Important Role in Disease Resistance against Stripe Rust Fungus and Ear Heading in Wheat. PLoS ONE.

[41] Shin D., Moon S.J., Han S., Kim B.G., Park S.R., Lee S.K., Yoon H.J., Lee H.E., Kwon H.B., Baek D. (2011). Expression of StMYB1R-1, a novel potato single MYB-like domain transcription factor, increases drought tolerance. Plant Physiol..

[42] Xiong H., Li J., Liu P., Duan J., Zhao Y., Guo X., Li Y., Zhang H., Ali J., Li Z. (2014). Overexpression of OsMYB48-1, a novel MYB-related transcription factor, enhances drought and salinity tolerance in rice. PLoS ONE.

[43] Wang Y., Wang Q., Liu M., Bo C., Wang X., Ma Q., Cheng B., Cai R. (2017). Overexpression of a maize MYB48 gene confers drought tolerance in transgenic arabidopsis plants. J. Plant Biol..

[44] Zhang L., Liu G., Jia J., Zhao G., Xia C., Zhang L., Li F., Zhang Q., Dong C., Gao S. (2016). The wheat MYB-related transcription factor TaMYB72 promotes flowering in rice. J. Integr. Plant Biol..

[45] Yong Y., Zhang Y., Lyu Y. (2019). A MYB-Related Transcription Factor from *Lilium lancifolium* L. (LlMYB3) Is Involved in Anthocyanin Biosynthesis Pathway and Enhances Multiple Abiotic Stress Tolerance in *Arabidopsis thaliana*. Int. J. Mol. Sci..

[46] Tiwari P., Indoliya Y., Chauhan A.S., Singh P., Singh P.K., Singh P.C., Srivastava S., Pande V., Chakrabarty D. (2020). Auxin-salicylic acid cross-talk ameliorates OsMYB-R1 mediated defense towards heavy metal, drought and fungal stress. J. Hazard. Mater..

[47] Harmer S.L., Hogenesch J.B., Straume M., Chang H.S., Han B., Zhu T., Wang X., Kreps J.A., Kay S.A. (2000). Orchestrated transcription of key pathways in *Arabidopsis* by the circadian clock. Science.

[48] Lu S.X., Knowles S.M., Andronis C., Ong M.S., Tobin E.M. (2009). CIRCADIAN CLOCK ASSOCIATED1 and LATE ELONGATED HYPOCOTYL function synergistically in the circadian clock of *Arabidopsis*. Plant Physiol..

[49] Wang Z.Y., Kenigsbuch D., Sun L., Harel E., Ong M.S., Tobin E.M. (1997). A Myb-related transcription factor is involved in the phytochrome regulation of an *Arabidopsis* Lhcb gene. Plant Cell.

[50] Kyung J., Jeon M., Jeong G., Shin Y., Seo E., Yu J., Kim H., Park C.M., Hwang D., Lee I. (2022). The two clock proteins CCA1 and LHY activate VIN3 transcription during vernalization through the vernalization-responsive cis-element. Plant Cell.

[51] Farre E.M., Harmer S.L., Harmon F.G., Yanovsky M.J., Kay S.A. (2005). Overlapping and distinct roles of PRR7 and PRR9 in the *Arabidopsis* circadian clock. Curr. Biol..

[52] Nakamichi N., Kiba T., Henriques R., Mizuno T., Chua N.H., Sakakibara H. (2010). PSEUDO-RESPONSE REGULATORS 9, 7, and 5 are transcriptional repressors in the *Arabidopsis* circadian clock. Plant Cell.

[53] Kuno N., Moller S.G., Shinomura T., Xu X., Chua N.H., Furuya M. (2003). The Novel MYB Protein EARLY-PHYTOCHROME-RESPONSIVE1 Is a Component of a Slave Circadian Oscillator in *Arabidopsis*. Plant Cell.

[54] Zhang X., Chen Y., Wang Z.Y., Chen Z., Gu H., Qu L.J. (2007). Constitutive expression of CIR1 (RVE2) affects several circadian-regulated processes and seed germination in *Arabidopsis*. Plant J..

[55] Guan Q., Wu J., Zhang Y., Jiang C., Liu R., Chai C., Zhu J. (2013). A DEAD box RNA helicase is critical for pre-mRNA splicing, cold-responsive gene regulation, and cold tolerance in *Arabidopsis*. Plant Cell.

[56] Jiang Z., Xu G., Jing Y., Tang W., Lin R. (2016). Phytochrome B and REVEILLE1/2-mediated signalling controls seed dormancy and germination in *Arabidopsis*. Nat. Commun..

[57] Rawat R., Schwartz J., Jones M.A., Sairanen I., Cheng Y., Andersson C.R., Zhao Y., Ljung K., Harmer S.L. (2009). REVEILLE1, a Myb-like transcription factor, integrates the circadian clock and auxin pathways. Proc. Natl. Acad. Sci. USA.

[58] Meissner M., Orsini E., Ruschhaupt M., Melchinger A.E., Hincha D.K., Heyer A.G. (2013). Mapping quantitative trait loci for freezing tolerance in a recombinant inbred line population of *Arabidopsis thaliana* accessions Tenela and C24 reveals REVEILLE1 as negative regulator of cold acclimation. Plant Cell Environ..

[59] Xu G., Guo H., Zhang D., Chen D., Jiang Z., Lin R. (2015). REVEILLE1 promotes NADPH: Protochlorophyllide oxidoreductase A expression and seedling greening in *Arabidopsis*. Photosynth. Res..

[60] Barak S., Tobin E.M., Andronis C., Sugano S., Green R.M. (2000). All in good time: The *Arabidopsis* circadian clock. Trends Plant Sci..

[61] McClung C.R. (2019). The Plant Circadian Oscillator. Biology.

[62] Xue X., Sun K., Zhu Z. (2020). CIRCADIAN CLOCK ASSOCIATED 1 gates morning phased auxin response in *Arabidopsis thaliana*. Biochem. Biophys. Res. Commun..

[63] Adams S., Grundy J., Veflingstad S.R., Dyer N.P., Hannah M.A., Ott S., Carre I.A. (2018). Circadian control of abscisic acid biosynthesis and signalling pathways revealed by genome-wide analysis of LHY binding targets. New Phytol..

[64] Bancos S., Szatmari A.M., Castle J., Kozma-Bognar L., Shibata K., Yokota T., Bishop G.J., Nagy F., Szekeres M. (2006). Diurnal regulation of the brassinosteroid-biosynthetic CPD gene in *Arabidopsis*. Plant Physiol..

[65] Lee H.G., Won J.H., Choi Y.R., Lee K., Seo P.J. (2020). Brassinosteroids Regulate Circadian Oscillation via the BES1/TPL-CCA1/LHY Module in *Arabidopsisthaliana*. iScience.

[66] Lei J., Zhu-Salzman K. (2021). LATE ELONGATED HYPOCOTYL potentiates resistance conferred by CIRCADIAN CLOCK ASSOCIATED1 to aphid by co-regulating the expression of indole glucosinolate biosynthetic genes. Plant Signal. Behav..

[67] Kidokoro S., Hayashi K., Haraguchi H., Ishikawa T., Soma F., Konoura I., Toda S., Mizoi J., Suzuki T., Shinozaki K. (2021). Posttranslational regulation of multiple clock-related transcription factors triggers cold-inducible gene expression in *Arabidopsis*. Proc. Natl. Acad. Sci. USA.

[68] Li W., Tian Y.Y., Li J.Y., Yuan L., Zhang L.L., Wang Z.Y., Xu X., Davis S.J., Liu J.X. (2022). A competition-attenuation mechanism modulates thermoresponsive growth at warm temperatures in plants. New Phytol..

[69] Farinas B., Mas P. (2011). Functional implication of the MYB transcription factor RVE8/LCL5 in the circadian control of histone acetylation. Plant J..

[70] Hsu P.Y., Devisetty U.K., Harmer S.L. (2013). Accurate timekeeping is controlled by a cycling activator in *Arabidopsis*. eLife.

[71] Perez-Garcia P., Ma Y., Yanovsky M.J., Mas P. (2015). Time-dependent sequestration of RVE8 by LNK proteins shapes the diurnal oscillation of anthocyanin biosynthesis. Proc. Natl. Acad. Sci. USA.

[72] Nguyen N.H., Lee H. (2016). MYB-related transcription factors function as regulators of the circadian clock and anthocyanin biosynthesis in *Arabidopsis*. Plant Signal. Behav..

[73] Gray J.A., Shalit-Kaneh A., Chu D.N., Hsu P.Y., Harmer S.L. (2017). The REVEILLE Clock Genes Inhibit Growth of Juvenile and Adult Plants by Control of Cell Size. Plant Physiol..

[74] Kwon Y., Kim J.H., Nguyen H.N., Jikumaru Y., Kamiya Y., Hong S.W., Lee H. (2013). A novel *Arabidopsis* MYB-like transcription factor, MYBH, regulates hypocotyl elongation by enhancing auxin accumulation. J. Exp. Bot..

[75] Huang C.K., Lo P.C., Huang L.F., Wu S.J., Yeh C.H., Lu C.A. (2015). A single-repeat MYB transcription repressor, MYBH, participates in regulation of leaf senescence in *Arabidopsis*. Plant Mol. Biol..

[76] Nguyen N.H., Jeong C.Y., Kang G.H., Yoo S.D., Hong S.W., Lee H. (2015). MYBD employed by HY5 increases anthocyanin accumulation via repression of MYBL2 in *Arabidopsis*. Plant J.

[77] Wang Y., Wang Y., Song Z., Zhang H. (2016). Repression of MYBL2 by Both microRNA858a and HY5 Leads to the Activation of Anthocyanin Biosynthetic Pathway in *Arabidopsis*. Mol. Plant.

[78] Zhao D., Zheng Y., Yang L., Yao Z., Cheng J., Zhang F., Jiang H., Liu D. (2021). The transcription factor AtGLK1 acts upstream of MYBL2 to genetically regulate sucrose-induced anthocyanin biosynthesis in *Arabidopsis*. BMC Plant Biol..

[79] Wada T., Tachibana T., Shimura Y., Okada K. (1997). Epidermal cell differentiation in *Arabidopsis* determined by a Myb homolog, CPC. Science.

[80] Zhao M., Morohashi K., Hatlestad G., Grotewold E., Lloyd A. (2008). The TTG1-bHLH-MYB complex controls trichome cell fate and patterning through direct targeting of regulatory loci. Development.

[81] Serna L. (2008). CAPRICE positively regulates stomatal formation in the *Arabidopsis* hypocotyl. Plant Signal. Behav..

[82] Zhu H.F., Fitzsimmons K., Khandelwal A., Kranz R.G. (2009). CPC, a single-repeat R3 MYB, is a negative regulator of anthocyanin biosynthesis in *Arabidopsis*. Mol. Plant.

[83] Kirik V., Simon M., Wester K., Schiefelbein J., Hulskamp M. (2004). ENHANCER of TRY and CPC 2 (ETC2) reveals redundancy in the region-specific control of trichome development of *Arabidopsis*. Plant Mol. Biol..

[84] Kirik V., Simon M., Huelskamp M., Schiefelbein J. (2004). The ENHANCER OF TRY AND CPC1 gene acts redundantly with TRIPTYCHON and CAPRICE in trichome and root hair cell patterning in *Arabidopsis*. Dev. Biol..

[85] Gan L., Xia K., Chen J.G., Wang S. (2011). Functional characterization of TRICHOMELESS2, a new single-repeat R3 MYB transcription factor in the regulation of trichome patterning in *Arabidopsis*. BMC Plant Biol..

[86] Tominaga R., Iwata M., Sano R., Inoue K., Okada K., Wada T. (2008). *Arabidopsis* CAPRICE-LIKE MYB 3 (CPL3) controls endoreduplication and flowering development in addition to trichome and root hair formation. Development.

[87] Wang S., Kwak S.H., Zeng Q., Ellis B.E., Chen X.Y., Schiefelbein J., Chen J.G. (2007). TRICHOMELESS1 regulates trichome patterning by suppressing GLABRA1 in *Arabidopsis*. Development.

[88] Tominaga-Wada R., Wada T. (2017). Extended C termini of CPC-LIKE MYB proteins confer functional diversity in *Arabidopsis* epidermal cell differentiation. Development.

[89] Schellmann S., Schnittger A., Kirik V., Wada T., Okada K., Beermann A., Thumfahrt J., Jurgens G., Hulskamp M. (2002). TRIPTYCHON and CAPRICE mediate lateral inhibition during trichome and root hair patterning in *Arabidopsis*. EMBO J..

[90] Wester K., Digiuni S., Geier F., Timmer J., Fleck C., Hulskamp M. (2009). Functional diversity of R3 single-repeat genes in trichome development. Development.

[91] Wang S., Hubbard L., Chang Y., Guo J., Schiefelbein J., Chen J.G. (2008). Comprehensive analysis of single-repeat R3 MYB proteins in epidermal cell patterning and their transcriptional regulation in *Arabidopsis*. BMC Plant Biol..

[92] Tominaga-Wada R., Nukumizu Y. (2012). Expression analysis of an R3-Type MYB transcription factor CPC-LIKE MYB4 (TRICHOMELESS2) and CPL4-Related transcripts in *Arabidopsis*. Int. J. Mol. Sci..

[93] Sawa S. (2002). Overexpression of the AtmybL2 gene represses trichome development in *Arabidopsis*. DNA Res..

[94] Ohmagari M., Kono Y., Tominaga R. (2020). Effect of phosphate starvation on CAPRICE homolog gene expression in the root of *Arabidopsis*. Plant Biotechnol..

[95] Zheng K., Tian H., Hu Q., Guo H., Yang L., Cai L., Wang X., Liu B., Wang S. (2016). Ectopic expression of R3 MYB transcription factor gene OsTCL1 in *Arabidopsis*, but not rice, affects trichome and root hair formation. Sci. Rep..

[96] Arteaga N., Mendez-Vigo B., Fuster-Pons A., Savic M., Murillo-Sanchez A., Pico F.X., Alonso-Blanco C. (2022). Differential environmental and genomic architectures shape the natural diversity for trichome patterning and morphology in different *Arabidopsis* organs. Plant Cell Environ..

[97] Marian C.O., Bordoli S.J., Goltz M., Santarella R.A., Jackson L.P., Danilevskaya O., Beckstette M., Meeley R., Bass H.W. (2003). The maize Single myb histone 1 gene, Smh1, belongs to a novel gene family and encodes a protein that binds telomere DNA repeats in vitro. Plant Physiol..

[98] Byun M.Y., Hong J.-P., Kim W.T. (2008). Identification and characterization of three telomere repeat-binding factors in rice. Biochem. Biophys. Res. Commun..

[99] Hwang M.G., Chung I.K., Kang B.G., Cho M.H. (2001). Sequence-specific binding property of *Arabidopsis thaliana* telomeric DNA binding protein 1 (AtTBP1). FEBS Lett..

[100] Schrumpfova P., Kuchar M., Mikova G., Skrisovska L., Kubicarova T., Fajkus J. (2004). Characterization of two *Arabidopsis thaliana* myb-like proteins showing affinity to telomeric DNA sequence. Genome.

[101] Karamysheva Z.N., Surovtseva Y.V., Vespa L., Shakirov E.V., Shippen D.E. (2004). A C-terminal Myb extension domain defines a novel family of double-strand telomeric DNA-binding proteins in *Arabidopsis*. J. Biol. Chem..

[102] Kuchar M. (2006). Plant telomere-binding proteins. Biol. Plant..

[103] Yang S.W., Kim S.K., Kim W.T. (2004). Perturbation of NgTRF1 expression induces apoptosis-like cell death in tobacco BY-2 cells and implicates NgTRF1 in the control of telomere length and stability. Plant Cell.

[104] Hong J.P., Byun M.Y., Koo D.H., An K., Bang J.W., Chung I.K., An G., Kim W.T. (2007). Suppression of RICE TELOMERE BINDING PROTEIN 1 results in severe and gradual developmental defects accompanied by genome instability in rice. Plant Cell.

[105] Hamaguchi A., Yamashino T., Koizumi N., Kiba T., Kojima M., Sakakibara H., Mizuno T. (2008). A small subfamily of *Arabidopsis* RADIALIS-LIKE SANT/MYB genes: A link to HOOKLESS1-mediated signal transduction during early morphogenesis. Biosci. Biotechnol. Biochem..

[106] Raz V., Ecker J.R. (1999). Regulation of differential growth in the apical hook of *Arabidopsis*. Development.

[107] Lehman A., Black R., Ecker J.R. (1996). HOOKLESS1, an Ethylene Response Gene, Is Required for Differential Cell Elongation in the *Arabidopsis* Hypocotyl. Cell.

[108] Yang B., Song Z., Li C., Jiang J., Zhou Y., Wang R., Wang Q., Ni C., Liang Q., Chen H. (2018). RSM1, an *Arabidopsis* MYB protein, interacts with HY5/HYH to modulate seed germination and seedling development in response to abscisic acid and salinity. PLoS Genet..

[109] Wu P., Peng M., Li Z., Yuan N., Hu Q., Foster C.E., Saski C., Wu G., Sun D., Luo H. (2019). DRMY1, a Myb-Like Protein, Regulates Cell Expansion and Seed Production in *Arabidopsis thaliana*. Plant Cell Physiol..

[110] Zhu M., Chen W., Mirabet V., Hong L., Bovio S., Strauss S., Schwarz E.M., Tsugawa S., Wang Z., Smith R.S. (2020). Robust organ size requires robust timing of initiation orchestrated by focused auxin and cytokinin signalling. Nat. Plants.

[111] Lee W.J., Truong H.A., Trinh C.S., Kim J.H., Lee S., Hong S.W., Lee H. (2020). NITROGEN RESPONSE DEFICIENCY 1-mediated CHL1 induction contributes to optimized growth performance during altered nitrate availability in *Arabidopsis*. Plant J..

[112] Peng H., Phung J., Zhai Y., Neff M.M. (2020). Self-transcriptional repression of the *Arabidopsis* NAC transcription factor ATAF2 and its genetic interaction with phytochrome A in modulating seedling photomorphogenesis. Planta.

[113] Tiwari P., Indoliya Y., Chauhan A.S., Pande V., Chakrabarty D. (2020). Over-expression of rice R1-type MYB transcription factor confers different abiotic stress tolerance in transgenic *Arabidopsis*. Ecotoxicol. Environ. Saf..

