# Supplementary material for: Characteristics and Functions of MYB (v-Myb avivan myoblastsis virus oncogene homolog)-Related Genes in Arabidopsis thaliana
Source: Genes (Basel). 2023 Oct 31;14(11):2026. doi: 10.3390/genes14112026 (PMC10671209; doi:10.3390/genes14112026)
Supplement: Supplementary file 1 [file genes-14-02026-s001.zip › File S1.pdf]

>transcript:AT1G18330.1 - Up_Stream_Len 2000
agtatgagatactttgcaaaacttttgattccgaagctaaatctgtgttggaggtttgcc
ctttcgctgcaccactcacatttatgacaaaactgctgatctaatataatatgtgagtgc
ttcttttgctttttatcttggtattgtattattaagactgctatgattgctttattttgc
aagaagaatgatgaagaacctaccaaagattgagtatcaagcatggaatagcacatatag
attcactgtaagtttccactttggtctactggagatatcagcaatcgtttattgtttagt
tttctgaaaaatctgttgcttgaatctgacagaccacagaggggtgtgataagatgcatt
tagaaaacagacgaatccgatgaagacttggttcctccaatgacttggttcacaccagag
gcagatacagaactccaacaatgcctagcaactgccaaattgaccattttgaaacctgag
tcctcgcaacaaaccagtcgtttgcaaccagcggagactaaatttggtaagctgtgagta
cttctaggctttcttaaccttacggggagattgtgcagttatatactaggactaggactt
agtagagtgagaacaattgttgaatttggttttgatataatgaatcagagagatatcttt
tcttttacaaaattgccaaaagtattgaatcatgagtgcttgaaccttaatttattgtag
atagacaaatgtgagatagaatatgaattgcgtaacatagaaggacttaaggacctcatt
atcatataattaaattgaggaaaagatcttgttctttttcatagtttggggtcagaaag
cgatcattttaatttaaaacatataatgtatatatcaaaaatgaaacaaaaagataataa
taaagagaggaatggggtgatctgtccgtatcaaaattgtggctctaatagtttgtgtga
ttaagaaatatagttgtcacatccgacaccatgtgatgcgacttgatttgcccattatcc
caaccattttataatcttttgtcatgaacttttctttcatacagttttataagactaaag
agatattttgaaatcgaattactacataatacatatcaattactcaagtatcaaatcatc
taattaatcaagtgattttttaactcaagaataatgtatttttccctttacaaaaatcg
aaggttacaacttacaaaactgaacaatgagactagttttggcctttttgggggcaaacc
ggcaaagagtaatatcacgaaagcaagtggaagtcagagacggatcggaaacacacaaaa
aaaggatatcttccaaataatcacaaaaaaatatcttcttcataatttgctaacgtttca
catttttatccacagaatctttttctgtacggattagagaaacagatcagattctttga
agaagacacagcgcgtggtgtacactagcgagagtgagtgttagctttgttctgagccac
aagatatctctctctcagtgtcaaccttctcgaaatttccatgaacattaaataatgata
aaaatctgaatctgacgtggcacatctctgctatttaatataaaaaattggtcacttgct
atttctagtaccttctttctttcttcactctctcctgtctcctcttctcactgtttccta
tttcgtaattttctcgagaaatcgtcttttcttgtttacgctaatttgatttctcgagaa
aaaaaaaaggaagcagaagaagattcttattttatctttacactgttcagaaatcttcgg
tggacggataacttgcagagttctgattgaggtttctttttcttttccctgttttctctt
ggatcttgtttcttcatgtgcggactatacaaaaaactgattcggtgaaattaatcagct
ttatgctctgttttgttcgctttcaggctggttttgtgagaattatagttgcagcaagaa
agcgtttcagatatttttta
>transcript:AT3G10113.1 - Up_Stream_Len 2000
gactcgtcaggctctggaagcaaaacgacaacgtcgatccccgggagagccagtcactcg
acctcttcatccgatatcgcatcatgttctccgacctcttcatttccttggggtttagta
atctatcttggattttagggtatagtttttaattccattgcatttcctaaatgtttttag
tattctatcttggctagtgtactagtctagtgtaccgaagtggtgtactgttctagtgtt
ctcactttgtgtactatcatagtgtacgcatttggtgtactagtcttgtgtactaatggt
gtactgttctagtgttctcacttggtgtactataatagtgtacccatttggtgtactatt
ctagtctacttctagaatgtcaaagtatcaatccaaattataatacaatatatttcaatt
caagtgttatgaagcataataataatctagggttatgactttgaattaggaatttattta

tttacttggaattctaaattctagtaatctaaggctttagggtatagttttaaacttcat
tccatatgatatatggttttccttgggtttagtcttctctctttgtcttttagtgtatag
ttttgaatttcactacatttcaataatgtcttttagtgtatagttttagggtatagtttt
taattccattgcatttcctaaatatttttagtataatagtagactagtctagtgcaccaa
agtggtgtactgtttttaattcacttggttactaattggtgtacccatttggtgtactat
tctagtatactattatattctatcttggctagtgtactagtctagtgtactagtctagtg
tactagtggtgtactattctagtgttctcacttggtgtactattatagtgtacccatttg
gtgtactgggtttctctcaaacgtctctcttctcgaactcgttcaccctcgatttgttgt
acaccgactgtggctagggttcgtgaaaaaccaagaagagtaaagtaactcaatctcaga
ataaacacgggctcagaagttatttggataaaaaacgtaatgaaactatcgggctcaaat
atttgggctcacttaatacgaattggagttaacgggcctaaaatgagttttaaaacccaa
tccaatttgtttcgtcgaacccagccggaatgtgtgtttttaaaacacaaggctactatt
gtatttgacacctcttttggatttttgctaccttttctatctcacatgaatcctattag
acaaacccaataaaaagtaccctctcagcatatttccaaaaaaactgtctagttctcaaa
taattttctaaaaactgcctacttttacaataaacccttgtaaaaactatgatactgcag
ttaaatcctatggaatgattagacatatgatcatgaaagcgacatatgtgattgagttgt
ccaccaagaaagaataacagtaactggctcaatatgattttcttttgatttaaatattgt
tttaaaaagtatatcttcattacgaaaattatgttttggttttggtgtcttaatttttag
tttggtttttcatatagagagggacatatctaccaattctaacttgttgatagccgtaac
caagattcaagaatagttacctgccaccacatcttcgcggcgtgtatctcaagcgtcatg
atcgttgacgccgtctccaccagatgtttgtcgccgtaagctaaccctaataaagcggcg
gagtaataagcgtttacggcctcgcttgtgctctcttgattccggccgtcccagaactcc
gttaaccctccagcccatgagtgtagcttgaaaagatcaaaatttctcaaacgcggataa
ctagaatttgaattattatcatcttttctcccaaacgtcatgaaatccgccaatagactg
tacgcttgagctctgtatctctctccccacaaaggatcaaacttagcgagcacggcaata
gcgtagagaaagtaaccaat
>transcript:AT5G37260.1 - Up_Stream_Len 2000
ttcaaattttataaaattttgtaagagtgatttgattgaaaatggacaacatttggagac
attttcaaaaaaaaatgttataaaaggttattttaaaatgctttcaaaaatatcaatata
ataaagagcgtgattatattttaattcagaattatttaagtgatcgaaaatatcataaat
tttgtataattagagcatttgttctgatatgcgacatattttactataattttataggaa
ccatataaatttaatttttaactaattccagttgcacatttgtttagatgaaaatgatt
tgattgctgcatgatataatttatttacatttaagcatatattttattctattttgatga
attgtgtggtattttcgtacttaaattttgatatattgtatgtaattgtacaacatactc
aatcatatgttattacttctaataatttgaatatggacatactcagaaaggtatcaaggg
ctctttctctagctattgtttctcatataatcttttcaaaatcatatatcatcaccaact
atactcatcattctcgggtactatgagctctaactagtggaatggtactagaactttgac
acaccgtctagttgagattatagaaattaaagattatgttatatatagtagaaacaaagt
aggccaaattcttatagaaaagaataaacaaaattaatataaaagataaatgaaaaaacg
gtcttgaattataaacaatttgctaggtgtgattcaacattgtctttattcaatacttgt
tgggtcataaacattcaaattgcatattattgttactctagtattcaaattgcatattat
gttttatctaaactaacttatatatttattttgataaacatgtcatatgatattaaactt
attttcaattgatcatcttctaagttgatgcattttagtattttatattcaaattttgag
atcttagtatgtaatatattttcaggtttttactattcatcagtttgatgatgcaaatat

acatagtacgaataaaatgagtgaaattgtttaattatatgatcaggaagaaaaaataat
attccaaaatccaaattttagtgggtaattcttagcacaaaaacaaattttgtttccaaa
tatatcacatatttttcaatgttccacaaatagcattaattgttgaaatttgttatgaat
aagaatatgttctataacattatatgaattagaattatcaatagataaagtgtgtcaact
caaatcgctaaattgttgaaaacaatttaataatatattcatttaatatattctcatgtc
aatatgatatgattagatctatttagtgagatcttatttaattgttaattctaataaaac
aaaaatagtgattatattactaataaataatataattgtttaatcttattttatggaact
gtatttctcaacttcaattaaatactgtacctaaacctaaaattaaatatgcaatcaact
tgattaataaaaaacaacaaaatggggtaagaagtaaacaacgttgttaaacaagacacg
cgctcaccagtcgcgagtttggcaataatcatgaaatgagcttctttaaaaaaactttct
agtagataaattaaacaattaaaaatctccgacaagatttaacaaagaacagataatatc
ttctccgcaaagacaaaagtgaaagcgaagaagaaatcctccacgtgtcattagcatctt
ttaacctgagccacatgatactagtttctcgaaacccctctttacgctttttcacccaaa
tttttataaaaagaaaaacattatgcttacgtggcttatcttcttcttgctcttttaaag
taaccccttgactctgctcctttcataaacaacaactctctcaccaatctcaaaacaatt
ctctctctctttattcttcttcttcagcttcagatttcagatcttaaatcttcaagtctt
cttcttcttcttctgcaacc
>transcript:AT5G17300.1 - Up_Stream_Len 2000
ttttttcgtaaaatatgaaacaaagacaacgacaaaacgaaaaagaaccaaaaatagtat
ctcattctactttcacttgattttggaatttcatttatttagattgtgattcaaaaccat
ttcaactatgtaaaattctgtgcaatatttttcccaactaaacatataagtttgacatga
aaaagaatataggaaaatttagaggtccaaattttatatgtatatcggaatcatggtagg
atacgttatcaattgcatttgtatgaggcttgctattgctagtatagaagcatcatcaac
aaatcttaatatcagtccaaatttgaataacccattttgtggatctcatttcataaaata
aaccattttagtgtcctgcaaaaatgtgtttagacagaaatctttttttaaaatctgaatt
tagatattatgatggttataatttatatataaatttactatcaaatattgtgttcatata
tggaagaatggagaaaaaagagtagaaccccacaaaataaaaaatggatagactatgttt
tctaagattggacaaagtgtataataaaatcttctgatctggtccaaaaataaaatgtta
agagttttcgaattcgaataatttgattttgattaagatatccacttcggatatgttttc
atatgtagctttttaaaataccctaataaatattttcataaagagtttatgtaaataaat
caaaatatatttttaactttttatttattatagttttatatattactgttttaattattt
tgaagtatttttgatagatatggatttggatggttccgcaaacaaatcctatggatccgg
atccaaagaaccaatcataaattttggaatatgtccacccatatatggatcaatagagat
atagttaagaaaagaaaatgaatttatcccgcaaatttacatttttatctcatgtagtgg
aaccaatgaaaagcgagtaagctaagaagtcatactctcgagagtacgcctttccatatt
ttcctttaaaagtatcttctggtggctcacatttctccgtccagcaaaatcttttttgtt
acataaatattatatttcctgatattttatattaaaagattatccaattgaaatgatgtc
aaaaaaggaaaggaaaagtaggggaattaaataaatacatacagtgataatcgaagtacc
aaatctaactgttttaaaacaagtagattcattttaaaaaattaaatatttgatttttca
aagagataaatgaataaatctagtacaaattttgttgcaaaaaaagaagatgaagaatat
gagaaagagcgacacgtggcaaagatggacattgaacgtgagccacaagattttccatct
gtttctcgagcgttccatggaaaaccaataagaattattatttttattttttaaaaattg
ggaaaaagcgcacatagcagaagctgacgtggcatcaccctcctttttgtcttccctatt
ttctcaccccccctaattcccttcttcctccttcacagaccccaaactcttctcttcttct

tctttctctctgttttcttcttcttccgccgaatcagaaagcttgtctttttctttactc
cggctaccaaaacttctcggagtttctctgagagtttcatttccgaccttcttcttatcc
ttatttgcggcaagatttccagcgagagtttttttctcttttcctttatcgaacataaga
aatctctggtttgctggaagtttttttttccggaaggtggaagcttagattggttctgatt
tcagaagcaaatcgtttgttgttgcctaagtaaaccgaatcaatttatgggatctataga
aacagacggagaacagaggaaattaagctgatttttttcaagtctagattcgcgaatttat
cttggtgtttccttttctgtgttccacacctttcgagtttttttttgttttcctccgccg
agatcttaatccgggaagtt
>transcript:AT1G01060.1 - Up_Stream_Len 2000
tctacagtgtgattggatcactagtgaccataacgggtctctacatgttcttgtgggga
agaacaaagaaacggaatcatcaactgcattgtcttcaggaatggataacgaagctcaat
atactactcctaataaggataacgactctaagtcgcccgtttaatttaattatattgttg
ttaatgtatgtttcaaataactgttatgtcctagttaatgtttaaggagaggtaatgaga
gataaaattttataattccaaacatcaacgtagagatttgtgaaatatttaaatccggtt
tgtttggttattttggaataatttcggttatttcaattagattcgggtagttcagttctt
cggttagtaacaaaaactggtctattgtttttggttaacctagaaccgaaccgaactaa
ccaaagttctcggtaaccttttgtagtggcttcctgaccgatgaggccgtcaacttcaaa
aaatattgcaactaagctctgctccaacattagagtatctataactatgttaaacgcttc
tgctttaagcaaaacacagttgtaagctggaatctaaaaaaaatgagtgtaatgatgtttg
ctgaattccataaataaatactacatgcttcggttaagacttaagagtaattaatgttcc
ttaatttctacaaatgttatataagcaagttgaccaaagttctcgatgataatttgttga
aattttgtataggcattgcatgatattatatgaaaagatgaagatttttatacagacgca
agttccccgagcagtccaagcttgtcgggtttaattcaactatgttaatacgcaaattta
tatagaataggcgtaaaagtgaggcccatacaatgtcttattacaagcccagatccagca
tagccaatacgtagcagtacaccatcacagctggcaccgtacccacttgtttagtcgtcc
aagtttgtaccaataatcgtttacacgtaagcaattgtggaccaccacactcacttttac
ctacgtgagcttcacattgaagcttctggctcgtagagaagcaacttgagatataccaaa
aagtgcagtagacagccactacaatatcaccacgtgtcgatctgcgatgacttctgtttt
ttccatttatacccttggtgctgttccagcctcaaataaacttttcaattaaaatttttc
caaaaattaggggaaaaattgttgtggctgagattgcttctggcttctcttcttcttctt
ccagtcttcttcagcctaaaacagtcttccttcttcttcttcttcttcttcttcttcttc
ttcttcagttatcttcttccttcttctctctgtttttaaatttatttttagagattttt
ttttgttttgcttccgatttgattatttccgggaacgatgacttctccggggagttcccg
gtgagatgataagtcagattgcatacttgtctcctccatggctactctcaagggtataac
agtttacattatgagcagtttctaggattcctataacatactaagatctctgtttggctg
ctgagaaacttatagaagcgattaactaaatcttattagctctaaaagttagcataaatg
atacgaatctggtgattgattactgatatgaagatttgtgaaggttttggctgcggtgga
ttcgtttgggtgaggcttttgtgaataataataaagggaattcttttgagttctgctgga
gaagcagcgactgtttcacggtggactttgaaaagatttctcttttgaatttcgctcatc
actcttatcttagtgtttgtggataaatatttctcataaagtactttctcctttgcagtt
tctctagaatctaaagaggttatcacaacggctttgcaatttgaaaactttcatgtttgg
ggagatcaaagatggtttctttttatactttacttgttagagaggatttgaagcagcga
atagctgcaccggtcctgtt
>transcript:AT2G46830.1 + Up_Stream_Len 2000

agagctactactgaagttggtgaagctcctgccactactaccgaagctgagactactgag
ttacctgaaatcgtcaagactgctcaagaagctgtaaatactcttactttatttatacaa
tgatgattctacctcttgcttctgggttacatgtactgaatttggttgtttggattgaag
tgggagaaagtggatgacaagtacgctattggttctcttgcctttgctggtgtagtggct
ctttggggttctgctggaatgatttcggtgagtagaagaatactactttcttcttaaaac
cctagtgttaaatttcctttatttgattccaaaatttgttattgtgaaacaggcaatcga
taggcttccattggttcctggtgttcttgaacttgtaggcatcggttacacaggagtgag
tttcttcttctctttgtatcacttgaaccaaagctctcatgaacctgttttgaggatata
gatgattcatcacttcacttttggatttaggattagttctctgaatttagaatccgaaca
tctgcaattcatatggagatatgatatcagaaattgattgctgcttctcgctagtgtttc
aatcttaaaagacgtgtgtagtttgtttcaattgtgtgatggaccttattaacatttggt
ttttctatggcagtggttcacttacaagaacctggtcttcaaaccagacaggttaaccaa
ttctctctttaactctgtgtttggttgcatgtaatactgagaatggaagactcaaattct
cgaggaaattgtttgttatctgtttcagggaggctttgtttgagaaggtcaagagcacat
acaaagacatattagggagcagctgaatcaaaggaggaagaagaagaagaagagcctttt
tgaggccattcatgaattggaatgaaggatatcaaaagaatctaacacaaaggccacgtc
cttccttcaatctttccttcttgtaactaaataattttcatcctttctctctctctgtct
ctggtctttttttagctcaaagtatcatccatttatgtcaaagtgttgtaaattcctcaag
actatatatgagatgttttgtttcattttccaaaatttcaaactttgtccccatttagtc
ttctacccttcatgcatggttagcttagcttaatgctgaactgttgaataacgatatggg
ccttatgctaaaagaacaaaaccttatgggtctaaaaaaaataagcccaatataaaacta
tggcccaaataagtttaggtccattagagtgtgagaatagcgcgtgtagtgaaccgcacg
agaatgcgcgttcgattgttggtgaagtagtcgtctagattcccgggtccactgatgttt
ctagtgtatcagacacgtgtcgacaaactggtgggagagattaacgatcttaagtaggtc
ccactagatcaagatattataacgaattgaccttttttaacctttcaggtagtcccggaac
tcgtggcctagaatacaaagaaggttgtgaacaagttgatgttaagatggacaagaatgt
aacttgaacaaaagctgaatcatctcttcagccactagtatgttgacatatggcagtttc
ttttgtagcctcgaaataaataaattaaaaagtttgaggttaaagataattatagtggct
gagatttctccatttccgtagcttctggtctcttttctttgtttcattgatcaaaagcaa
atcacttcttcttcttcttcttctcgatttcttactgttttcttatccaacgaaatctgg
aattaaaaatggaatctttatcgaatccaagctgattttgtttctttcattgaatcatct
ctctaaaggtacttaagattgatttattgtcatggtctttcttattgtttgatgaataac
ttgacttgattgtttttttgttttgtggattagtggaattttgtaaagagaagatctgaag
ttgtgtagaggagcttagtg
>transcript:AT1G01520.1 + Up_Stream_Len 2000
agataagggagaaggctatctctatcttgcattcatttttcttagatggtgtaattccaa
gtaacactgtttctgatgaggaagttgaggaaagtgaagcaagtgaagaagaagaacaat
cacctagcaaacacgagaaattagcaatagtggaatccaccagtaggcaacagggagaaa
gtactctcaccagcactgagatcgtacgtagagaggctagtgagttaaaagaatctctga
gccctggtcagcaacacgtttctcaaaatactgccgtaaaacctgaaggaagacgtagca
gatccggtaagaaagccaaaaagagacattcacagcaaaaatacatgcaaaaaacggatg
gttcctcagggttaaatgaagaaagtacttcacgaagagatgatattgctatgagtgaca
cagaagaagtattaagttccagttctagatgtgcttctcctgaagattccagaagtagga
aaacacctcttgaagtaatgcaagagtcttccccaaatcagcttgtaatgtcaagtaaga

agttcattggaaagtcaagtgagctactgaaagatggatatgtagtagccttgtatgcga
aagacctctcgggcctccacgtttccaggcaaagaacgaaaaacggtggctggttcctcg
atactttgtccaatgtatccaaacgagatcctgctgcacaattcattatcgcatacagaa
acaaggtaaaccttttctctctcttacttttcatttatcttgcttacaatgccagatag
accattataaattggttttggtgcatgaacttgttttccaggacactgttggtctgagat
catttgctgctggtgggaagttactgcaggtagctcttacattagagagtgttacttcca
ttggtaactcaatgttgctcttatggaatctaaaagtggttgtgtcatgggtgtgtgtgt
gtgcagatcaatagaagaatggagtttgtgtttgctagccatagttttgacgtgtgggag
agttggagtctagaaggttctctggacgaatgtcggcttgttaactgcaggaattcctct
gtaagtctctgtccttacagaaaatggcccgaaattgaaaaaccctacttcttggaaaac
agaaataatttgtgtaatgaatgttgcaggcggtgttggacgttcgtgtggagatattgg
caatggtaggagacgatggtatcacacgttggatcgattaaaaagaaaaacagagtctct
ccatttgtgagtttctctcttttaattacttttgttactttaacatccttaggattcaca
gacgaaaaacagagacacccaatttttgtgtttcgagactgtgtcgtgtgttgtgtagtt
ggtatcaaccaacttatatctgtaatcattgtttctttttatttattctcggtttgcaga
aacatccgatgagcttgtcttagagggacgtttgttgttgttttctgggtctggtcgtga
tgaactcgaaagcattgtgtgtttggttagtagtttgaaataggtgtgtgtattgtattt
gtatatgctgcgtttgtgttttagagatcatcgtacataaaacacatcatcgtacataac
taaaatttgagctaaactacaaaagaaagtaaccttcatttttagtcgaaccaggcccca
gctaggcagctatctcgtaaataagattgctggcttacgatcgtattccacgtggcaatt
tatgtgccgtggatttaaatttgtacgtggcatgagtgttaggagaatgtccacatggct
tgtagttgttagtcccacgctctgaaccagagcaaccggctccttacacgtgttcggctt
aaatccatttttcgaatgagattacacttctaaccttgtctccctctcccgcttatacca
ccaccactctcacacaagtctctcaagtcacaaactctgtttcaaaccaaaagggaactt
tgtgtgtgttgtcgagtttt
>transcript:AT4G01280.1 + Up_Stream_Len 2000
ttctgagtctaatatcttgttttgcatccagggtttctgatttaagcttggaggaagatg
atgtactgaagttggccttgttggggaataacgcgaagacgaaagatacaatagacacac
tagtcaagtccttggttattcggaataggtttgtcacatagattagttccactcagttca
catggcttcacttagttttggttatttgatataggtaggtgcttagcacttagttgtcac
tccttccctgctctcattttggtcattgacttagtgaacttggttcattatttcccattt
gtcatgtctacagcagtgtaatccgattaaaaaagattaatttgaccttcagtgacttat
gaaatggtgatttaccttagttcattcgtaacaacaacttgttaaactgaaattacgaaa
tttcataagtaggtatggtcaagaatttgaggaaaggaggattgaacattgcgtttcttt
tctcttaggtctttgaattttcttttatgaagtctctaataaaaaacaatctttcttact
ctgacaggagttataaggaattgctagccaagtgcaatcagctgggaagaggcgaagcac
gatcttctgagaaacttgaaaaagctttggaaaagatagaaaaactaaaggtgtgtgttt
tggtgtttatctcgatcttctttgtgtcttctatatctggctcttattatgtttattcta
tgaccagaaacgaatgagggaacttgagttgataactgaagagagagagagaacagagctct
acgggacataaacgtttcaaagaaatgcagttacacagaagtttccgagcctgcaatcga
gagcatgtcttctttcagaatgctttcatcagacaacaaagtggagaaaatctccacacc
acctggtaaattagaagaaaaggatggctttaccattcaaggatcatgcttaaggggaag
agaagactccttcgttagcagaacagactcggttatagatgtagatgatgattatgttcc
tgaaaccaacacttctggcattagagattggaatactaatattgaagagaaaggtgataa

ttccatggtgaaggatataaagttcaacatcagaaaagacccgacatcatcagtgtcacc
ttacagcaatggtacttttgttgtacatcatgaattcaagttcttgagtgtagtagtaga
ttgaagattgtccaatgcaggtagtggaaacatttggcagtcaagtggaacaaatagaaa
ccttggtagatggagcaaacatggagagagaaacgaagcaactccatcactaggaggttc
tgttccaagaaaagatgatctcatctctattggacctgatggtaaaggtggtagaatcaa
agtgctgagatctaaaccccaaatttctgtgagttttacattcttcttcagtttctcctc
tcattaatttccactaccttgcctaatgtgttgatatctgtgcatagaaaaccaatgcaa
gctcaggaagtggtaagagattcaagcttggaactaaaacaagtggctcgtcctctcaag
gttgtcttcagatagaacactatttcggaaaaactaatcgctaaattttcgacaatgtaa
ctttttgttggggctagtaaacacttgtagttaaagaatgaagattaatactactattta
aatagtggattagtgataaacaaaaattagtagttactagccaaaacgtgagagcttctc
cgtcctcgagatcgtctccacgcggacatctttatagccgttgattcttggtttaagtga
taacactagtttaaggcgtcagtggtatagtctgggtactagagcaacgagaccctaaca
cgtgtaaaggaaacaatattatgctcctgaaattacaattctacccctctgttcttcagc
cacaaaaaaaaactggcgaagtcaaaatcttttgacagtgaaaggacctttgaagaagaa
gaatcgcgatttcgagctct
>transcript:AT5G52660.1 - Up_Stream_Len 2000
ctttttccttcacgtctatcaaagtaactcctacaacaacaaacattattgcttaatttg
tattaagcctaaatagattcaattacttatattatatatagacattgtttgtacatatta
taatatacctggaaagtcacatacaactttcatagctcttcgtttgattctttcctcaaa
aacttccaacttgaatacaactttctcctgcaaagatttagatttgaagctttagacagt
tcattgtgtataagtagtacctttagttttctaagattttaagtaactaaaagaaaagac
ataccggcattttcggaaagtatagaaagtttgatgattgaggagatatgatgtaaagga
atatgaaatcaacctcgtagcttgctcattaacctttgtttcgccccatatatatacacg
atatcctttgcttgaattagcgaatatgaaatggttaagtcaagccatccaagcaagaaa
ctgaaatagtcaatttgatacgcttaaatgaagatgattaataaaacaggtttgagtctt
tctatataatagattgttagaatgtgttgttgcgcctcttttgtttagtactttagtagc
ttcaactaataccgtgtcataactcataattatgcatatgtaagatcttttgtcttttat
catctcgtcgatataagataataagagcgattaaatcaaccctatataataataacaata
taagttttctatttcgtttcgtttaatgaaaatatgaataaaattgggataagtttgtat
cgcctttttttctttttcacaataagtttgtatcccctaatttaatcatttaaatcatat
tatctacggctgctatcatgtttacaaatgttctttgtattaataaatagagatgttgtt
gatacgtaaatttcagcttcagatttttaataaaattatacattccttaataaaaatgtg
tgaaagcatgaggaaccacacaactcccaaaccacacgacattatcgtcatttacttggg
ctcatacgaagcgttacagtattagtaaacgccataatttaggaaattttcagtatagtt
atacaaatctatactataacttttggtactgcgctttagccaatgtcattaattcagtta
cgtaacagactccaacgccaaaacattagtataaacaatattaagaacgtggcattacca
acaatccaacaaagatgttctcacatcgaaaccaattagaaacctaaaagatatagtgaa
ggttgatctctttctttaacatgccacatttttgcatttcttcctttatttgcgaggtta
aaatatgttcatttgttaaagaaatttataaaccaatgtaaaccatccatttaaattctc
attcacaaatcaatcgttctaatcgaaggatgccaagaaaaaaagcattgttgtcttgtt
gattgggctaaacattaagacaaaaacagtggcatcaccaagtttttttttgacatatcg
atgtttcataatgggctaaatatataacccatttgcccattagtttttttttttttgacg
ggcaagttgcccattagctaaaaaggctccacaaaacataaaatttggacaattagaaaa

cctttacctaaactacaaaacaatcctaaattcaaccaaaaagaaaagaccctttttgg
tataaacggatacaaaacataaatggaaaaatctttattataaagatccaaatacgtggc
acgttatattaggtcacaagaactattttgccgacctcaaaaacgatcacaagaataagt
ttctttcatcgagaaacacaacatatacacaaaaaaactatagcttataaatttattaat
attttaataaaatctctgccgtcttcttctttctcataagccacatttcttctctctttc
tctcctacacaacacagctttttaaacccaacagagcaaaaaagcaaaagattcaacaaa
aatctcctcaaattctattc
>transcript:AT3G09600.1 + Up_Stream_Len 2000
ttttctttctgttccatgtcttcttaactgctatgagtaaaagagctctcccagactctt
cttctatctccaaaagcttttatttttgtttgtttattttggtactgataatatctgagt
atatatataatatagtagaaacaatgagcaaaagtcaatatgtccgagtggttaaggaga
ttgactcgaaatcaattgggctttgcccgcgcaggttcgaatcctgctgttgacggaatt
gtttttctttttagcttttgttgggttgtgaaaagtataacaaccttaataatttcgttt
ttttaatctaaaaatatacggtagttttctcatcatcaccaataagaattgtttgatatg
aaacaacagaatcaggaaaatttgttaaaatgtcagaaaatggaaaaatctctaactgaa
caaaagtaacaaaaaaaaactcccttagaaacagaggaatcatgatgagagacaaaattat
acagagacgtggagatgtctgtaacgttgtctcatccatcatcatgtcttcttcttctct
tccttctcttttccggtcatccctccgtcctcggaacctccatccccgacgcggtgaaca
cggcggcgcgtttggtaaacagagcgagaagagcaaaactgtcgagagaatttctacaag
cgcacaacgatgcacgtgtttcttcaggcgtgccaacgctcggatgggacagagacctgg
cccgattcgctgataaatgggctaaacaacgtaagtccgattgcagtatgatacattcag
gtgggccttacggtgagaacattttctggcaccggcgtaagaagacgtggtcgccggaga
aagtggtgacgagatggtttgaggaaaggtttaactacgacgtgaaaacaaacacgtgcg
caccgggaaagatgtgcggccattacacgcagatggtgtggagagagaccacagccgttg
gatgcgcacgtgtgaaatgccataacggtcgtggctatcttgtggtgtgtgaatatgatc
cacgcgggaattacgaaggtgaaagaccattttgatcaaaacagttaaaatgaaaaaatt
gttagagattcaaaatatttttttcttcttctaatgtaatgaagatggtatattattcttg
gtccttacatatatacagccatgtattgaagaggtataaattcaaatggttcggaatctt
ttaattttatatattaaagatcaataataaaaaaaaacgtcgacagcagggttcgaacct
gcgcgggcgaagcccaatagatttcaagtctatctccttaaccactcggacatatcgact
tgttgtatttgtttcgtaagatttgaatacaaaaccgaattctctgttgcctaaatcgga
gcttttcatcccttgactctctaagacatctgaagcagctaaagtgttggatgtttcaca
taacgacatgggcggcaagcacccagtggacacaacaaggtacctttgttcttggccgcc
ctctaattcgggtgaaattggaagagaggtgcttggtatatactaggatgcgtatttggt
gcttagagatttgatgaaactgaagcagttagatattagagacaacgacctaatatttgc
aggtgaagagttcagctcttttgtccgccaggtttttccaaagcttgtatggcttgatgg
ccacaaactcgcaagttaaagataaatgttaggccacttgttttaattaaagtataaaac
tctttcgttttttttggattcattttcatgaaatcaaaagactacaagtctacacgtgctt
caagcttgaggctctaaatgaagtggtaggaaagaatgaaataataataaaaaaagtcag
aaaaatggatggtctgagatcaaagtgaaacctactccacatacacatcccaacttcttc
cccgccatgtggacttttctttttcctactttctctctctgcattctctcttacacctgt
ctttatctgaaaaataaccg
>transcript:AT5G02840.1 + Up_Stream_Len 2000
cattattcagagacttagggatataaaagcaaaaactaatgataaacttagttttccaaa

aatactgatattgtacctttataatagtacaatatgtaaaggcgtccaacttcaacaatc
cggaagattccattcgttttgcttctttgtatatatcctgggcaagatcaacccgtccag
ccaggcaacatgttttcagtagtatgttgtaggatgtcatgtcagctgtaacatcaagta
tctgcccatgacatgtagagtttcaaggtcagtccacacccactcctaaatctaaaatac
tctctagacaattcaaatacttgtattcaagagacagaagaaagaacatacctgcatatt
cttgtatacttttagtgtgtacccaagatcatgggaattaacattcattagactgttaat
aacataaatatttggcttaatgttttctttgagaagatcctgccacaaaattatgaatta
actacaaatctcaaatgcaggatggcagacaaaatcgagcgtaaaggatacttacgcaaa
aatacagtataattagggaattacctcatatatgtacctcgatttaacgtaatcaccaca
caggccacaaacatctatcatcgttcggcatatatacatgttaggagtatccaaaatctg
tttgcaggcttcatatgctgtcataacagagaccatatctcctttctttccgaaaccatg
aataattctacaaagcaataactctgtatgcggcaaaagacatgcgtatctgcattcaat
aatcatcaaatcgttagcagaacacactacatccagcattcttgtaataactttctgaat
ttgaaaaccattaacctgaatcgttatgtgggaaacaaggcttacctaatagctaattga
ggattggaaatttcaacacaactctttacaacatcaaaaggatccaccaactctttgatt
ttgaatccgagacctgaaataagaagcaggtaatcaattcaagagttcatagtcacagtg
taacagcaagaattgatatagtatttaccggcaagtatctccattagatcaatagctttc
tccacttgcacagagttagccattgctcgaaactgtttcctcattaatttcacagacgaa
tcatcgacaaggtccaatggagcaattcctaccttctcaattctcttcaaagtgtaaacg
acgctctcaatcttaccttgtcgaagattcgacgaaatccctttcgataagagatcataa
tcaaccatggaagcgaaacgagccacattagctccggactctgcagccaaagtctcggcg
atgagagcgacgtcctcaattcgaccatcctcagctagcttcgaggcaaaatcagcgtaa
tactcaagcgagccatctggaatccatcttacgacattggagaagtggctagagagagaa
tgagtcgcggagactgaagcaggagaaggtgaatgttgctgcggtagtgacggaagtagc
ttggtgaaagaagaattggatttagttttccggttggactctggtgctgtggcgtaacag
cgacggtggtgatggtgaggattggttatggcggaggaggagccaaagacaataacaaag
tctctcattcctcgccaacggttaggatcatcatcgtatcatcattgtggctcagttttt
ctggttttctaatttcatgtaaatcaaatggattggatttgaaaggctatttttacaact
tccacaccggaccggatttgtagtgttaaaatacaccggtatatataaaccgatttggta
ctttgattttaaattttaagaaataaaataagaaaaaggagaaaaatggatggcctgaaa
ttgcaagcgaagcgtctccacatattatacaccaacttcttgcccgccatgtggatatcg
tcgtcatttctctctctaacccccttctctcttcagtctctctctctctagacgatctcta
tcttgaataaaataccgata
>transcript:AT1G19000.1 - Up_Stream_Len 2000
aagaaaaggtaatggtgtctttcattcataaccaatatatgtgcttttccttttcttttt
cttttccacaagaaaatgtttgattaataattagaagatttttatatctttcctatcatg
cagcatgcatgttgttaaacccccatgcttaaacttcgatgggatatgatcatgacacca
cgttaatttttaagttaaaaaaaaaaaaaaactaacaagataaagcgtaaaagttttaagt
ctctagtttgttttaaatcactaattgcaacaacaaaacatttttacaatggaatacttc
taatgttgaatgatttgagttaagacgaccaaagattaagaagataaaccagagagagtc
gatagagatagaaaggttaagttcatgtgctgaaccaagattattggagaagctcatcat
atactcttttctttttgttaaaccgtgttacatcattagctagactccaatttttttttaa
gttttatgatcattgtgatttgtaaagaaatttgttatagacattgaacttgtaatctct
tatttaatttgcaaaaaacaatatttaaccaactgatcaacaacggtcatttaatcgtac

aatataattcttataaaatatcatgtttatttgcgaacatataaagtcaaccccgatcag
caacaaccttctccaaaatttgtgtgaattcaattatttcttcactatatttgcaagaat
gtggttatactatacttttgtgtgatattttcaattaaaatagagtttagtgatatgatc
cacatgacaatatcccttaaaacatgattagtcagtcgaattaatcggtcatgtgtttca
ctagtggtgtgaatgtgaatcaatcagtttgtaaactatgaggagattcatacgttaatc
atgcttgtaacttgtaagggattatcatcatatctggttttaaaaggacaatttgcaagg
gaaattaaaactttaaaaggctattaattgaaggggaaaaggaaaaaaagaagaagagaa
ttgatggatacataaaatactagctagtatttaaagtgatgaggatatcatgtgctccca
ttggccccatgtcttcatcaaatattttgatgccacaatttggaatattcttttgattag
tttatcaatcatttaatcctaaccagaagtaatttgtatttcaataaaaacaatgttcta
aaagggatttggaggattaatttagattaaagcgaaggatgaaataaagagtactaacag
attgagattatcaaggtaactttttggttgcttgtgtgattcgttacaagctagctaaaa
taagataatggtttttatataaaggtaatcaggttggatgtaatcgggaatcgcactaag
taagttgaccagattcgtttaacactaacattacattataaaacacacttataataccat
cttatacagtttaattatgaactaccattcaaaaatcacatttcatcttcatgcgcatga
cacaccatacttcctttaccaactttttttttctcatttctatattcaaatcatcaataa
gaaaatcacactagaaaccatttttttttacgtgagagaaggacgtcgagacacattcact
ttgcttgaacaaaaaaaaacacatttaattaaaatgacaaaaataatgaatagagagaga
gacattatcagaatatatccaaagaaaataactcattaattaataaatccaaatggaaga
aatgataaatatagagagacaagcaggtcacagaggaaacgagtgacgaacaaatcataa
ataaacacgtgtcatgttatcgtaataacacacaacagagtaagtaatgccacgtggctt
atcgagtttttataaagacaagacagaagaagtagctcagatccactcacacctcgtctc
ctaatctgtacggttcttatttcgaaagggtaaaaaccaaaagcgacgcaaagggcaaaa
tcggaaaaagtgttttattt
>transcript:AT1G74840.1 - Up_Stream_Len 2000
agcagtgacacgaaccacagcggtaaccctacacaacgaagaaccaggacgaaaaaagaa
ctagcagggagcacagcttaagaactagacaacgtagtttctgttgccaagtccagaaat
caaaggcctcttctgccttcctccatatagactggtgggtgaaaactgtgggcgtatttc
acaagcactccatatccatgataactatgctatggtttcaagcagtagagtcaacctctg
ttagacagaagaagctggagaagaagataaatggtgatcttagtttatatgaagaagcgt
ctatatgaaaatctcaagtaagtagccaactaagtgttttgatggattgttagacccatg
attttgcagccatagattttttttcgttttttttccattcaactatgtttttgattact
ataaagctaaatgctaatgtaagatcaaagctctagttacaattaaaggatctattaatc
aagattcctcagggaagctctagtttttgcttgtgacctcacctgatgaactctctctgt
ttcgaaatctaacttattacatagcaatgacccaactccacccagaataaagaaaacaat
ctgttgtgcaagattccttcttcggtctgattaatctttttctctctctcgtcctcagat
atgtgtttgttttctgatttaggaaagtcactacaagtttgtcatccaaattggtaataa
aataagaaaaagattggagaaagaaaggatcttcctatagtgcttcacacattacaactt
taagattccactttgtgtgcgcataaaagtaatggtggctttgctttctaaccgatgcta
gtgctttgtgtgattttgctgtcttccaaaactatttaccaaattttgtaaatacttttt
agaatcttttccctacaaatctaaaatagataaataataatagtatgtgatcatattgct
tacagtcaaatcacatcctaacattctactaaaagactaaaactatcacctttttgtttt
ttaatgctatatagttgtgatcaagtctggacatacgatcatgctgtttatactacaatt
aggtccttaacagcgtcttaaaatttactcttagctagaaaacgacaaattcgactacac

aatcatttttgttgttgaattagattcctgaaattgtaagtttttgtgttgaaattcaaa
gttataatttccaaaaaggattcacctgtctcccaagattttttcttaataatcaactc
atagatccacaagatatagtattttagaagaaaaaaaagtacaaggatggagctgtaact
aaatagattattagagcagagtacagaatcatttcagaagctgactatttttctaagctc
aagttgcatgcgcttaggccttagagacattgcgttgaccagtacattataacctgtaga
tttttatcataaaaatacatgagggtaacgttaagaaaaataaaagcgtcgatttcattt
catttttctcattctgttatttcattttttgtatttaactttgagtttgcgaaagtcaa
taaatatttcttcgttatacgacctacatttttggtcgacatagcgaaatacttttta
acttcttattataggataacatttatttaactctttttcgtcatcctataagattaatt
aaactactaacaaaaaacctaataaatctagtcagccaatgaataaaaaaagtctatcca
aatgaaaactctgtagagagacaggcaggtaagaaaaaaatacaagaacagacgaataaa
aacaaaacacgtgtcatatggtaccgtcaccaacacctacaacatgtaatagtttaaaaa
ggaaccacgtggcttttggcttataaaagggtccccaaagaagctcagatctaactcaca
ccactccattgcccattgctctgtgtactttgcttctttaggagaaagaaagcacaaggg
gcaaaatcggaaagattatc
>transcript:AT1G70000.1 - Up_Stream_Len 2000
tacaacaatctaagttatatacattaataaatacaaaccttcacctaagaaaatatcttt
aacccgacataaatttgttactacggacatgtttttgtttaattggagaaagatatatga
ttgtgtatggtgtctattgattgattgtcacagaagtataggcattttctgttattggca
aactaagaaagaaaagacattttccactagttatgtaatgcatcttcgaaatacccaacg
gaccaaacctgctcatcctttccgtttttgatattatataacaatattttatatctttta
atcgctaaaaacgaatattatataaatgtattggatgtccaaaaaaaaaaaatgtgaatgg
aggctttcatatgctatatcactatcaaccaatcacagaacatctttgacagaaggttac
ctatgcacgtacactacacatacgatgctctttgtttatatctttgcttatagtttatcg
atgctaaccactgtaagaattaagtaaagttaatgccaatttaacattaagttaaaatta
ctgtaaaaagtaaattagataggctgccttttaaaaaaattgttgaattataaccatatt
tgcttttcaaacatgcatgtcccaagtaacagaacaaacaaaaaaaaagaagaagtttggc
gtaatgctcctataccatgggttagtgctctcaagcgggtcatgtgtacaccaacaatat
caatttttgggtgtttgcttttaactgaaaaagtaaacgcatcgaatctttattatgatt
tcgatgaataatgataaaatttgggacaatttcaattcaagtgaggaaaacgtgtctgtg
ccttcttggtaaatctaacactgaaatttgacacgcagttgcttctatagtcagtaattc
caataaagcatctaaaattgcatttgaatatttaatcatagtcgtccctatcaataattg
tcaccaacaaagataaagggtaaataagaaacatataatgaaaattgaatggtcgtagtt
ttatatgttagttggaaccaaagattcttcaatctatcatccactcagagctacgtggac
agtcgatattttgggttatataaaaaaaaacaaaattgtactataagacaaaagaaaaca
gtattaaaaaaaaaaaaatcaataacaagattatatcttatcccaatgaacaaaatgaaa
atttagttattttgaataatgttacaggttttaattaaattatactatatcacaaacagc
aagagtcagatacatcgaacggtccagctttcctcctttatttttttctggctatcgttt
tgatgcaaagttgtttcgttaatcgtgaaaattaaaaagctacagcaatcttctccgaaa
cttcatacactcgccgacaagagaaacttcatccactcggatttctcgtaacttgcagca
ctaaacatttcatgaaactattcacaaaagtccacgaaccttgcaattggccacaaaatt
taaaatacttatatacgtaacgacaacctatccatatttttctttctaacgatccaaaac
tggccacgcttcgactccatggtgatattaactagggaagtggctaatggcaaacaagtg
cttagaagtggtaaattcaaataataaccgtcgattatttctttctaaccgttattgagg

ttaccaattaaaaaaagtgatcacaaactaggtcaatgaaaagggagattttttgttttc
tttgggccaatgaaaatagaaactaagatggacaaaagaaaatattataattggacaaaa
cagagtcatttgagatttaaaacagagaagtaataaaggtcaaatgtttttgtggtgagc
tctcaaaaacagacataaaaaaaagaagcagaatcattcccatttataacaaacctaaac
ctctcttttcttcttctctttaccttttttcattccaaaatctctctctctctctctct
ttctctggctcgggaagaag
>transcript:AT5G47390.1 + Up_Stream_Len 2000
acactgtactttcagtttccaccgaaaaataattcattactcatcatttattcaatgttc
gataatttcttggaagcagacagcatttgggcctcgagatttaaattggcccaaggtcca
cgtttacttgggtcaatcacagtcaaaacgttttcccggggtttctctcaattttttgt
caaagtctctcaacttttaaaaatacaaacactgggatcctccacggcatgcaaaaggat
attataatgaagatgaaatcaaattggtataaaaagttggagaaaggtataaacactatg
tatgtggctaaaaatagctagaaacttataatttaggtgaaagaagatttgagtaacgtt
gttgttgttatataataagatgaaataatgacaagaaatctgaaataatatgcgaatatg
ccaccgacgggagaaatacacgtggacggctgataaagctttgtgaaggaatcccacaat
aatataagaagatctgaccaaaggttcctttctggataagccgctctcctcgtgtccacg
tggcattttcaaacgtgggacctcctaatctatatcactaaacccatccactcatattat
ctctctttctatcatctaaaatccaaaatgttcgtccattttttttttctgttcataaa
caaaaactgcattgccttttaatttattcttcttctaatccatacaaatgattatggaac
tataaatctataataataatacaattaaccacaaaataaaatatatcttagtattataag
atatgacttttggagttagggggtgtctaatacaaatattatggtatttgtaatcttagc
aagaaatgaaatcacaatcatgtttatctttcttaagataacatattttactcagtcatg
ggtctacttttattttcagtttagattattaaataaataacaatagttttgtcgccttac
tatatgaccaaaaattagattttctctatgtgtaggatgttggcatttgtttaccatttt
tcttatgataacttattaaacagatgtcatattttacttatttgtgtttataactgttaa
aatctagtcaagctttctgtatggaacgcgtgaagggctattaagatattttaaaatgtt
gaatagtgtaattaatcttgtggctaataatattcatgccaattgttaatttctggtgga
gggttaacacttgtacataaatacttaatttttctttttgcagtttttgtatagatatcc
atccttatataaatagaaaaatataaggaaattgtaatgatttagcataagacaattgga
taatcacaaatgcattaaacctattaactagtctatatagaaagagttattagttacagc
tatactatttgtggtggcattaatatatagtaacttatttaagatatatgtcataatgtt
aaaaagaaatcacagagaaagctgttattttttttaatcctaaatttaaaactttggata
ttgagattagcctgtaatacatctttttccaaaaaatttatacaaaaatggccattaata
attaatagtaatagctagttggacttcttagttcaaagtaattcaacatttttttctaaaa
taaatcttctaataatagaacacgttggattgactaattaagtcaaatatgaaatatgga
aaaggaaaaaaatccaccaacgatacaccaaataatttccgatttttatatatataaata
aacatactaagagcaaaagaaaaacaaactgaaaataaaaaaaataaataaataaaaaaa
aaaatccggccagataaatcgaatttatgtaataaatccgaccagataaactgatattat
tgtctttcttccgctcctttgtctctctatctctttctcacaattagattctgtgcttct
tctgcgatcaactaagatccgatccgcgagcgtttcagacttcgatcagatccgattaag
agaagcaaatcgggtcgggt
>transcript:AT3G16350.1 + Up_Stream_Len 2000
tgcagctgtttttgccggagctttctacggacttttcaacctcttttctggttttgtcat
tcctagaccggttagtgttggttacttttctggcaagaaagctgaaaccctaaatgattc

tctattgattctatcataagctggttttactttcctcttgcagagaattccgaaatggtg
gatatggtattactggatctgtccagtggcttggactgtgtatggattgattgtttcgca
gtacggtgacgtggaggatacgattaaagttccgggtatggcaaatgatccgacgataaa
gtggtacattgaaaaccactatggatacgacgcagattttatgataccgattgctactgt
gttagtgggctttactctctttttgcgtttatgtttgcttttggcataagaacgctcaa
cttccaacaacgatagggtgtagttgcctgccggatatgtttggcgtgaagtagaaaaaa
atgaaaaatgagggtttatgccatatagtttgtgattgatgctttattgattgtaagtaa
agtgtacaatatatacaacacaatagattgtggggagaatttacaaggacaaatctaatt
ataattaacttaagatctaaatttatgtatatcctaacagttatatccgttttgtacact
attttaaaactttttacagatctaagataaacatagatttattttaagaatcaaattgtg
aaaactctgcgtggaaaaaattaatcatgagtcatgacaatgatgagctagttaaggttc
caagtgattgcaatcacattttgtgagcactaaagaacacatacacacactatatatata
ttcttgatcacctaacgagtgaatcaaaagagatgagtagtttattgtattttgggacaa
acttcgattaaataatatttacactatttcatgaaaacatttgatcactcatcaaataca
tcttatgaaggcgcatatatatctactgtttatgaaatctaatctactttactatatgaa
aaaaacatacaaatttgccggagctttctaaagacttctcagcttcttctccggtattat
catccctagaccggtcaattttggttacttaagttgcaagaaatctccgtgtaacccaaa
tttcataaatgcgtagtgggatttactcttcttctttttggtttcatatttggtgtttg
cctttcgcgtaaaataccaacatatagcaaagagcgtttagacgtcaaatgtgtttgttt
tatttgtgtagagtatacatactttttaagaaaagaaaaaagaagttatgaaagcttgt
tctttttgttaaagattatgaaagcttgtttaaatcctaatcacatctcgtactaaata
aagaactatatgtgtgtgggtaatattatccaatctctttaaatttaaaccacttgtctc
atctcatatgcaaaacgtgtatcttgtaagaacacacgctcttctaatatagaaatagat
cgcgtaacaagtggacttggaaaagctatagtttatggcgttttcggacagatttacact
taaataatattatcctcacatcttttaaattcaattatttcattatcttggttatcagaa
ctgagttttgtcggtaacattgtatctttaaaaaaaattatcctaaacaaagaacgaaac
gatacaatagtataaatttgttcttgtcataaacgataatttccaggtaagattaaggat
aaaacaaaaaagcttataatagcaatttaaaacacgaattcaacgtacgatccaccacaa
tcctcgtcgtctttcaaacttaaaataccgaaactacccttaccttcttctttccataag
aaatcaaaaccaagtcctatggtcgcaattgtatctctatctaaaaatcttctttgaatt
tgtgtatacacaaatcttattcaacgaatcatttatcaccaaaaggttctctctttttg
tttgtttctgtcttgtgtga
>transcript:AT5G56840.1 + Up_Stream_Len 2000
gagtgttaatgtggaagaaagttttgaagcagaagctgaaatattatactctttgaatga
cattactcagagccaagtggcaatgatggagagacaaagtgaagtgttgtcgttggtgca
aaagaatcaaaagatgctaatgcagatgcaaaaacaaatgatgatgttaatgcagcagaa
agatcatgtgttagcacactccgaatcttatgaggaggagatgacaaaagtgatcgagtc
tatggagaaacactacttgaagaaaaagtagttttgtttatttttgtttggttttggttt
tttctttgtttggtgttcctgaaagttgattaattactgatcatggattcattcataaaa
ttcgtctttgcataaattgtggacttcaacttttggtaattgtgagacttcttactcgaa
acagagacttattatattttgtcacctcttaaaacagagaaaaacagagaagtatcgatt
tagagatcaaaagaatgtgatgtgtgctgttattgtggatgaagagcatttgcataatgt
tctaatttggtagttttttccttcaataatatatgatatagtgagcaacatctttagaa
tatgtttagtaaatttttgtaaaacaaattgacctacctcaccaaaaacaaacttcattg

gaccagaattaagtagccattaggatcaaatcaacaataatatactatttatgtgatgaa
atatgaatccccatcattgtcctatatgaatcaacatttaatatattttaattagtcgaa
tcttaaaaatgaatcccagtcatattatctttatgtatgtagaagaagatataatcaaca
ttaaatagattattatagtacatcaacatgaataccccatcttgtcatattatatgtatg
tgggatagaatcaacatgaatccacattcagtattcaaattagaaatgatcgtaggctat
gactctaaagttaattttaatttatatgatgaatgcgagtacccccattatagattcata
ctatacattattttatatgatcaaatatgaatcatcatcattgttctcctatacttgctc
ttcattccgaatgtagcaatagaatctccatattgctttttaccaattctaaccataaac
aattcatagataaatgcatgttgggtagtgctttgtctactagtaattaataacattatc
gattacaatatttgagcaaatcaggcaatctttaaataaaaaatatgtatacgatgactct
tccgtccaactaatatattgaaaatattataagatccaaattatttcattatttatatca
aacaaaaacaagaaataaataagacttgtctttgttcatgtaaacaagaccactttgttg
tttgtttctctattttacaaatttgtaaattatatttttgttatgtttctttccacattt
ttattaggtcatccaactacccacctccccaaaactattggaggatccaagtcctctctc
tctcaaccatatatcttatatttcctttgcaaaaggatagcctttattatttaatacaca
tagattccattattgttatggctacctcattctattctataaagcatacatatgagaaga
agaagcaaaatatagactatgtgaaagataaatgaatatttcatatttgtgagacaaatt
aattagattagaattaacattttgtagctagtccatattagtgataaatttccacttata
aagacatattttcctcttttgttttttatacgtaagcttgtcttcttattggctaaaagt
agaaaacaccaacttggacaacatcatttaaattataaaaccctccaataaataaaactt
ggtttatccaatcatcttatttctctgtgctctccaccattttacataaaccccaaaatc
tctcttcatcatcatcattcatcaacatcatctctctttctctatctcgctgtccctcct
tctctttctcattcattgca
>transcript:AT5G61620.1 + Up_Stream_Len 2000
taatgctttgattgtaccaattgaaatttgttcatacatgcattgaccacatttttattt
tgtgtgttagttaacattatgtatgtgtgaatgacacttaggaaagaagagtattccaga
gctactttgcatgcacgaggaggtaggcctgatgggcctaacaagctggcagaaagtggc
aagcaatcaggaggagataatccgttgaaggaagacaagccgccagaaagagataagctg
ccgagaaaagacaagccatccaaggaagacaatctgctgaaaggagataagccggtcgag
gaggataaattgccggctgaggaagaaaaaccgccacaaaaagataaaccggcggaggga
cacaaaccgccacaaaaagataaaccggcggaaggagataaaccggtggaggaagacaaa
ccgccacaaaaagataaaccggcggaaggagataagcatgtggaagaagatatgccttta
ggaggagttgagcatttgtcaatccctgagataccaaaggtactatattagtgatgttca
aattttttcgttaatgatttttcacatttagcaattgccgggaaaagaatttggattag
caagattaaacactgttctgctttaatattcccttcataaattaatggtaggttatggtg
gtggagcaaaacccaattgtccccaagtgttgcggcccaagtacttgtactaaaaggtgt
cacccatttcgattcgccatggatgctaatgctactaaagcacgatctatttttatgaaa
gaataccaaaaagtcccccgtgtaggagcttttgcatataaatattttacatctcgtgac
aaaaaaatacgtaaaccattttcatatatgacatttgggacttgggtggttattattttaa
agggtacgcttttaccttataaagagttcaaacgaaatttatcattttgaaggtattaaa
tactataagctaatgtgtttttgtttataaatatgctgctattaatgtactttagaaagc
gtaaatttcagttatgaaaaaacacattaaacaggtggaatctctcaatttgtcagatta
ccatgtttttttattttattttaatatatataaaaattgcaatcggaatcttagtcgagatt
aaataaaaatctaaaactcagaacctactagattttaatttttgatcaaactaatcatat

tttttaccaccgtagattttgtatgtgtcacacactgactgcaataaacaaataaacaaa
aaaggtttccctaaataaggaaacgaggactttgttcctatattgatctaaatttccaag
tatccaattagatctcaacgcgtttagattggatcgaattatatcacaaagttatttaaa
aaaattatactctgaatttatcgcaaatatatataggagtatatatatgtaaaaactttg
gaagaaatagaaatatgggtaacttaggaaatatgagatattaactaatatggatgaaga
taaaagtaagtaaattccagttatattaagattaggattgatattgataaaaatatgggt
aacttaggaaagaaaataaaaaaataaaatcgaatatacgatttaatatctctcctaaat
atccgggatcaatatccagtacatgtttacctggaaaacaatctaacggctgattttcca
cgaatcctatcttaagaaaatgttaaccgtcgattatgatcctacgctgattctgctgga
tttttccttataatgacttgggtaaaaacttccaaataaaacaagataattaaggaaacg
ctttatctcttaaggaaaataaaaaaatgtaaaaacaccaaggacattcacaaataacag
ctcacgcatatccgcatatatatatatatatatatatatatatatcattacacaagag
agtttaaggtataaatacaattagcgagatcttgtgattctaattcttgtgataccgaaa
acattggtggcgagagagag
>transcript:AT3G10580.1 - Up_Stream_Len 2000
tgtataggataattaatgattctatcaagtatatgttagtttttattttaaatagtttca
aaacattaaaaaagccaagatattatattatatataaagtaagtgaatatgatcactaat
taaatgatcaatatttagtcaaaccgtttgtctttcagtatatttaatttacatggaact
tggaagcataataagaccaaaattttataagaagatatatactttgaattctttcttgaa
ctgaaaagtaatcttttctttctttctttaaattcaaaaaaaatagaatatttatttctg
tgataaaggccgcgcgtaaacacgtttaaaagaaagaaaattcataaagaagtcagtcat
acgtacttagaactctttcttgtactgaaagttttcatctttttattttattttttctg
tgataattaaacagacgtataaacacgtttaataaaaagaaagacagcttctgagttctg
agtttgatatatataatatggtggttaagaacgaaataaaataactcgggtttcttttat
taaataaagcaaagaggagggttaattttacatttttccttattatttctcattataaat
ctaatatgaaacgaaaaatctcaatgacattatatattgagggctaaatttgtagttttt
cttattagtttatccggcgaaatcacctctataaaattctcaggttctcaaaaaataatt
aaaggaacaacaaaccctcttcagtgacagacacagactgacagattccacaaactattt
ttcttcattttcttggatattatggctttgattccgccgtggacgagagacaatgacaag
cgttttgagttagccctggtgatattccctgagggctcgccgtatttttggagtacatt
gccgagtttctacagaaacctttggaggaggtgaagtactactacgacgcaatattggtc
tatgatgttgtgctgatcgaatcgggtaagtatgcattacccaagtacccggaagcctat
tacgtgtcactgacggaagcgactgagtccaaacacggggagacgaatcagataccgagg
ataattccttggacagaagaggaacacaggtttgtcaaagacataaatttagggtttctt
ttgattgaggaagagtcttaaatcttgctggagttgatgcattaagggttagtcaaagac
ttatgaattacatatgattatgataatcttggtggttgattttctgataaacgtctttag
attgttgaattaagatgatgaataatcgtgtttgtgagagaatgtttttatttgaccttg
ataatgacgattatgggatcttgatggattgaacaagtatgggaaaggagcttggagtat
gatatcaagggaatttgtgacctcaacacaagtagcaagtcatgcacaaaagtatgacaa
aaggcaaaaaattggacagtaagaagaggaaacgctggagtgtccttgacataactttgga
gtctactaagggcaagtcagattttgttaagtagaaaaccctccgggtgatgataagttc
aagtatatgagtatatgatgagttttgcttgacgatgatcaattgtatagggggatgttg
atgatgatgatgtactgatgtatatatcggatgatgatgattgatgaactgtataagacc
ggtttgaacaaactataaatgttttgggttgctttgtaaaagtataaatgttttaagacc

ctttggtatgttgagcaatatggaggttaaggacgaaataaagataactccggtttcttt
tgctaattaaagtaagacgagggttcattttgcaacattccctattattttctcactata
aatctgattagaagcggaaaacaataccctcagtaacagattccttaaagttctcttcaa
cattttcttatttttttgcttatatattagattcctgatctttagcgatctcaagatttc
tctctttttctttggatact
>transcript:AT4G09450.1	+	Up_Stream_Len 2000
atcaagtttggtgatacagaaaagaataaactaatagattctttaaatctgcaaacgatg
caatgattcaagaaaataaagttttgctaactgaaaagcttagctttataagaatgtata
taaatataagagttcattatcatcttgagtacataaaatttcatatagtcagatttgtat
ataaaagtataaaactaacaattgttttgcattgcagtataggtggttactgccacatat
atacaaattaagtcatgtaccagcactaatgacttatctcccaagagcagaaaggttagt
actttgacatttaccatctatgttgatttctggtcttcgaagttctcatcagttattttt
cactaatcgtcattgtaattccatagctcatcatttagcttcttcagagttcacaaaaac
attgtttcaagcacagattcaaacaatcagcatttgtggctcacaaatctcctttgtaaa
gatttattcttctaaataaaatttagtttgcagtaaaaaaaaaaaaagaagcaagagagct
attgagttacggattttataatagtggggttatgaatgaaattttaattactcgtgtttc
ttttgtaactattgtgggtttatgattggtagtctacaaaaaatcagaaatacctaagca
cctactaaagaaccaagaccaatatttgtacttgacttgtttttctatacaaggttaatt
ctttctacaactccaaactatgtatcaaaaaccatagtctgcaagcagaatttcagactg
ttcaaatccgtcagcctcaggtaggttcagccaatcaaacttaccctttgggagctcttt
aacctgctcattcactcctctctccacaatcacattgattctccacaacacgatcttact
ttcccatttcatgcctttgattgtatggaatttgtattctaacttcccgtaccattccat
ttgtttaggttcttgacaatgttttcaagatctctgaaagctctcctcacaagcccaact
tcaattcctatttccacaagctctctcacagaatctatcatcttatttgcaatctcctta
acggattcagcttggcttggaaattctctcaagtgtatggtcgcacagaatgcaccagta
aacgccaaaacccagcaactgttatctaccaacaagtctcttggtgggtttagccttatt
cttatctctgggagaagattctccatcacatgaatcacaaactcgtcatcctcaagcggc
gttgttaagcactggaagatataaaccgctctctgaaactttgttttgctttgtgataca
atataatcgcgtagaccgtcccatgcgatatcttggtgtttgaacatctcacacacaacg
tggttcaccaccttacctaggactctgaacatactgccagagattctttcttgcttaagg
caagagatgagcaatgtctggagttctctcatctctgacaattctattttcactttcttt
caagtgtgatcagagagtacaacttcaagtcttcttattgcaatctctcttgacctatct
tcaacaacatttgacacaaagattctcaagatatcaactgatgacaaagtcttcaaatgg
tcacacaagccacgaacatattctgctccatcatctttcgtttcctgaggtttcacgaca
ggtttgctcacagctttgtcctgtgttttaacaaaatctgactatactatttcctatatg
ccctattatttctccctataaaatctccctataaaatctgactacaaaatctgactatac
tatttcctatatgccctattattgttaaattttaatttgtccgataggccctattatttc
tccctataaaatctgactggaacaagaaaaaaccctcaataacaaaaccctcaatatttc
cttcaactaattgtttccttgcttctatcagattccttatctttcgcgatctccagtttt
ctctctttttcttagatact
>transcript:AT3G10590.1	-	Up_Stream_Len 2000
ggtctataatcttttgtatgttaaatttttaatttttaacacgtgtgaaaacataaattt
gtttatacacgtgttataatctaattggctgtaacacgtatcataattctgattgctaac
acgtgtgaaaataaaaatttatttgtacacgtatcataatctaattggctaaaatcgtca

atattataaaacaaatttaataaaaaggatataggaaactaaatgaatgacaataaatca
gtagaaagaaataaaataaagactttgtatgcaatatagcaaaatctgaaataaaattta
ccctttgaatataagaaaaaggaaggaaacttaatacaccccaatttgagagaagagaag
ccatatattttttgttacttgagttgatttataagctgccatatcccttacatgagttat
tatatatactgtagttctccagattcacaccattctgttttgtagacttcattttcctta
atttgttttgggaatcatggctgaaaattcatggacgacagaggagaacgagatgttcaa
ggatgcactggtgatgttcactgcctttttgcttactcggtttgagagtgtcgccgagta
tgtggatagatcggtggatgatgttaaggagcattacaaggaattggtcaatgatctttt
agagatgggatccagtcgagtagcttttcctaatgaattgactaaggatatggctcaaag
ttcgtaccaggccgagagaactatatggaccaaagaaacacatgagtaagtatataagat
taatgagtgatgagttatgatttttgttagttagtggaatttacatacacgtataagttt
caattataaacataatgattgattagtactggtattggtttgtggatctagctagacgca
ttctcctcttagactccataattcacatagttcatttctgagattaaaatgtatttagtc
ctatgatcttacgatttacacattctagcattattttttaatgtggacaatatagtttt
tttttttttcatttgactcaatttatatcgttaactaataatgccatggtgaaacttg
attaaaatgggctgatctatgaactggtggaaattgtaatgcagatggtttctgattggg
ttagaccgatttgggaaagattggcgtaaaatagccgtcttattggattgcaagagcccg
atccaagtcgagatatatgcagaaaacttttaccagtggcaaagctcaaagaaaaatgtg
attaacgacttaaatgtggcaagcaccgatgtgaatgtgatgaaacggcagggagcaaat
aacaccaatgtggactcaacaggccaacaagagagtctcgtcgctctggaaatcggtcac
catgctaaggcccccaactcaaagtgatataaatctctatgactgtattaacaaccaaaa
aataaaaagcctaaagatattgaaggcccttgattattttttttgtgtggtatgttaaag
atttgggatttccatttttttggcaaaccatattttatttaattttgtggtcatctatat
tattttctttaaccattaaaataatatatatttcaatgtttttatttttttgctttatttc
atctaggctaaacagaagatacacgtaagatggagctttcaagttaagtacgtagtatat
ataaagttggggttaaataggaaactaaaataacttgaaattacactccaagttttaccc
actgcgtacgtctaaacgtaaacgagaagaaaacttctgagtcaagttgtgcgtgtttaa
atacgaaatcgtgtttctttttgtaaagtgagggctaattttgtaattttcacccattac
ttgatcctctataaattgtcaggttgccaaaattaaaacgaacaaacaatcgctcttccg
taacatattccacaaaacgatcttcttgtttcttgaatttttagccatctcttttttttt
ttctcattttctcggatact
>transcript:AT5G58900.1 - Up_Stream_Len 2000
tgataatgcaaaacgcggatctcacgtggacccctttgtacaacttggccatgaaagtta
ccgctgatctcgaccgttcatttggcggctccttttccctcatctgacacataaaatccc
aatttgccaatattatacaaaatgtacgtaatcagtgaaaataaagattataaaagtata
cagaagcacgtatatgtttgttgtatattagtttaatatgaatgttgaaaacttggtatt
gtttttttcagaggcatcgtacactcccacaaaatgtcaacttcgcgtatttaaattaatt
aaataattatcatacattgtttttgagttttttttctcccttttttgtgcctcaatgtca
attattttgtatttcacaatttatcagagtgttcaccagcattattattttgtaattata
tcgttcacggaatttttcttcgattcattttattttctttctcaaagttccaaaattata
tttttcatgtctttccgtgtgaccaaatcaaacacaagaatttacgatttcatcattaat
cgatttacatttagttcgaactctaacatatttgttacgccatttatactagtgaatatg
taagacagttataactgtaaagttctaactatactaattacttttccattgaaaaagctt
aaaagtttttagggtccaattaagtagctaaagtttaatcaaaatgatttcaaaagtagt

agaaaaatatgcagttttgtttaaccggtggtgaaatgatgattatgaaattaagaaaaa
aaaatgtttatgattaagaaagagtttgtaactatgtataaggagtaaaagtaatcttga
ttaggaaaataattttaaaattacagcaaacaatttagttttcattttcaataatagtga
atataattaaacaatttataaaataaaataaaaataggagaaattgaaaaatggaccaca
tttggtgtatagatagacacggacggtagtgagcaatttttaagcggttcataaccact
ttattttaaattttaatacatgttttaatcatcttttctcatttcaaatatctatttgaa
gcgtccgtttaaaactttcaaccattttacccaaaactgttaagtcatgagtatttttaa
ctattaagcaggaatattactatgcaaggtgacaaaaaaatagaaagaagtaaaaaagag
aatcaaatcttaaattttaagaagaaaagaaaaaacagcatcaaccaaaaataataattt
atcaaggacagaactataagagcccaaatcatcacctcctttctctgcctacttctctct
ttttttttaactcgagaagtttataatctggtttctgattttagttttctttccattta
tgggttcatttgcttcttcttgcacgttatagttttcctaattgggtaagctccaaggta
gcttcttcaatattaatttcatttttttcattttctgtagccatatgtttattttcccga
gaaatattatccacacccagatctttaaatctcttttaactctgagtctctgatacttaa
ataccactcatgctgcttattagctccaccaaatctggaaaaccaatagattctttttat
ttcttctgcaaaaatctcagagctaccctaaaaattctctctttttgactcacttaccag
atctccactagattttactcaattctttttcaaaaggtttaaacttttatagattccttg
aagtgggtctaagcaatgcttcagagaaattccgatgatttcttctctaaccaaaataaa
aatgaagtatcatttgatagtgatttttgtgttcttcttttgattctcttatctcttcct
tcccatcaataaacccaaaaaaattaatatcatttttgggatcttattgatcccaaaatt
cgtaaatccgttgacctttgtcggcatgatttgttcttacttaccgtaaaaaaaacagag
ctaggttttagttttcggac
>transcript:AT3G49850.1 + Up_Stream_Len 2000
ttgttgttttgtcagggtatcctcctaaggaaggatatccaccagcagggtatcctccac
cagcaggttatcctccaccacaatatcctcaagcgggatatcctccagcaggttatcctc
caccgcagcaaggatacggtcaaggatatccagcacaaggctatcctccaccgcaatatc
ctcaaggtcatccaccacaatatccttatcaaggtcctccaccaccgcattatggtcagg
ctccaccgaaaaataagaaagataagaaggattcaggaggcttcatggaaggatggtgcg
ttgaccaattatctctctctttttatattaacatatagttttgaatacttgaagctgatg
atataccttttttggttcattattcataaattttggtatggagtaatatggatattcaa
agtctatatgtttggtacttataaaatgtgtatttgttttgtattacagtttggctatgc
tctgctgttgcgttctcttggaagcttgcttttgatcggaagatgagctgatatttgccg
ttgaccgtcttgaggatgctattcaattttgtcaaacatgttaaaaagacgtgtttgata
tacattttagatctcttcttctggttatgtgtgtccacgttttgggtttgtttctttaat
tttctttaattttatgaatgaatccgaatttgacttgaggtcttgtttactttcatgact
attgattgattcagggtatgttacccatcttttgtatatgtgtgttttatcttttgtata
aaagccattcattctttccaactagaggaacacaaaaaaagaaagcaacccaattttgtt
ctctttctcatcaaaaatgtaagaagaagtgactgtatcaacgtgatcaaaacttagcat
taccatcaaacttatataaatttatacttacggtccgatatatttccattttccgtctca
aacatatatgtcacatccaccattcctcggttgatcaactattcaatgtacaaattttta
taaaaatactactaaatcgtatataatcttgagtcttcacttcttatatttaatgtaaga
gagtggtttagtatccaccgattaactcggttcaaaccaaaaatacaccaaaatgatatg
actgaattgtgaaatcagtccaagaactaaaagtcgatgagctcggcatcgacctccatg
aagataaggaaagattcactcaaatcttaagaaataatacggtaaagaatattagacata

ttcccatccgaaaaataagaagctcgatgaccaaacaaaaaatccgtaattctattttcg
aaaaccctaaatttattatattactaaaaaatctcttgtaattgtcaagtttactctcaa
aaatcatttttcttgaatctcttgtttactctaatttctactttgcttttactcgcattg
ttaatttaatttcataaattaatccctaatagatcaaatgggtgtatttttgcaattata
aagtttaagacgaatttgtaaaaatgaagataataaagggctcccagtaaagcacctcaa
aatcgatacatacttcatcctccaacatgcgtaggtgaatcgaaagattaaaggtcaaat
ttttgtgtcttctcttcctctgtctcatgccacaatttttagatttcaaactttgatcca
tgacactctccatagcttcctctctcattcccttcagctttctcaggcatgtcttcttct
tcctcttcttctccttgttaatttccccatttccctgatcgaaagctttaatctttttct
cgtgcgtgtcttcgtaatctctggcgtttctattgaattactaatgcaattgaaaaactt
ggggatttcattataaagtattgatctttgtgatgttttcatctgttcacagagaattga
agtcgagacattagaagtgtttgttgtgtgtgtgtgtgtttctatagaatttttgaat
tttgtgataaaattgtgtga
>transcript:AT5G67580.1 - Up_Stream_Len 2000
ctccagctttagggttaaacacctcatatgagtagagctcgtaagtcataatgcggcatt
agcaaaaagatagttactaaccttcaataatggtgtgttgggcttcttgacctgcaatga
tttagatgtaagtttttcagtactctgaattctatattaagggatcttaaaacagaacca
tcacaatggtgatcgaaaaaaaaaaaaaacagaaccatcacattctcataaaatgtgagt
gaatgcgaccagaaaaaaaaaatgagctaaaaacgaagaaggaaacaacgtaccttataat
cccaaccatgacgctcagcaaaagacttagcagcttcttcagaatcaaaagcaagagcag
agtcaccaacattagcataggggtcacctgtagaagtccatcccatcaacggattctccc
acctagaaacatatgaatgccatagataagtgatgtcaatcgaaatctaatcaacaagtg
atggactagtactagtcacaagcagaagacttaagttttgaaaagataggaattttaagg
ataagaatcatattaggacataaaatgtgaggaaaagaggattatcttaatcaaaaagta
gcatactttaaggtggagacgaagttaatcttccattttccgagttttccagatcctgac
tgggtagctgttctagcaggtgagtatattatcacctgttaatttccaaattcaatactc
ttagcactattcgattgaccaaaatcaaatcaagaacacgatgtagctcaattaagacga
ttctctgatcacgaaccctaattttctaaactgaattcgtaagaaatcgaatgcttattt
accttgcgagaaagatgctcctctgggattccggagaccttaccgatctcaccacgcttg
taatccgactcaaccaccgcgtcagtagcgaaaggtcgagcaacacgccggagcgtcgcc
gctatacggatagtcctttgagtagtagcacaaagcgccatcgtcaattagagaaatctg
gaaaattgctgagctttttgatttcgctttggtttcttctacttctgtagattttagaaa
gaaaaaaaaaacacatatttcaatctagccatcaaaattttataaattacgctttcggtc
ttactattatatgaaaaatctttaatagcccctaaactatctgtgagatttaacaacgat
aaattttatagatacattatttttaaaaaatagtactagtatattttattcagcttgatc
ctattcatttagttggtaggttttattgtgtaatttgataaacttgaggcaaatttgtga
atgtggagaaatatagggttacatcttccttagcttgaatacaaaagaaaagctcaagtt
tgtttctcgtttctatggatattcttcttccttatgctagcctctctggtcttcttcttc
ttcgttcgtttcagcgaatcacatttgatctatgacacatctatacaaggaattagcttt
catcatcaagtctctctctcgaaaactctcctttggtttcgtttgatctggcatgcttct
tctcctccttcctcctttctgcttccttctctgaattttccatttttgtttttaattttc
tacgatcgccccatttgaatttggttgataatttagctatcgctttagattgatggctgt
atttccaaattcctgttgatttgcgacgttgttaatgggctttgatcctctttttttggt
tcgagattgctcgaatcactcatttttttgttgaattgagctttggggcgtatctaattt

ctttggatttgtgtagttacatttttatgttgtggctttgtgtatctgctggatttagat
gaccaaaattttgtattggataagaaatgttcaacaatggctttgatgtggttaagtttc
gtacgatttaacagattctaatgttagcgttcacatctattggcagagaaaagaagaaat
tcaaatattgataactgtgg
>transcript:AT1G49950.1 - Up_Stream_Len 2000
gtctttgttgccatatctcactcgcttcgagaagggctactctctttgtgatgagagctg
taaattcaaactaaggctttgcttaaaacggattcaaaagtgctaagtaatcgttaggcg
ggcaactttgttgactctttcttgctttttttactttctgtcttgtgttctcgtgcacgt
aattaacactaatgctatacgtagaaaaatgaaccggattaaaccactggtgtgacttaa
ccagaattgtccacttgtatattcatgtgaagaatatgatgtttaatattacaaagtctt
tataagtagcaaattattctcatcttaatgccaaaaaagtatacaactaactccatccac
cacttaaacattctctttaaccaatatcaagaattataactaatgactaattggtatgac
ttattgttggcttaatgaaaataagataaaacaataggattatcagttaaaaaatccaga
aagatttaaggaggtttattttgaaataaatttgatgaattattgttgctgacttgaatt
attatcaagtacatatttcatatcataaataattttgggctcatgagctaaaaatgttaa
tttggtaatttaataatttattttggatggaactaattgctaatgttttattttgactgt
aatttgaaattatttggacaattattaatttaaacccaaatatgaaattagtttgtatta
taaaatcataaatatctaggtgaaattagagaaggttctttctttttactgaacatctgc
agaaaataaacctagtaaaacaaatcaactaaaactatgttcaacgaacttttctttttt
ttacgaaatgatctttatatttcaaattttggagaaattcaaaaataaataaattataaa
actttttaatgtgtttaaaacaagtgtatagatttggaaaactctcaaaatatcgcaact
accatgtccacaatttttttttaattagtggtacagtcaactagataattttcagaaaaa
ataaacaatagagaaaattctatggataaaatgaaaatagcatttttgaaaaaaaatatc
acaactaccaccccaccacacatttctgaattatatctagcaacatatttgtattttatg
ttcaaaataccacttaatcatcaaatttttaatcaccaccaaaactttcaaacataaacc
aaattgttcgtgtaaatatgatctgggtcactgcgtattagttttgataatatattttct
tgacaatcatattaacacttcatttggggaatttgatattttctttatcattattaaaag
ttgaatttgtaaatatttagacaatttataatcttcagaatataataactagcgtaaaca
aaaacgcgttttaaggccgtttgtaaaaaacgcgttttaaggccgtttgtgaaaaaacgc
gtttaaagcttaattggtctaagcggtttgccgcatcagaattgacggaggtccgttact
ctgaatccttgtattcgcattaaccctaactttttctgcttcccaagcgtctcgaaatct
gaatccagagattaaacaaaaattggtattgcgaatgatcgattcttcttcctctaaact
ctgattcctggtgaattcatctgtttcttctttctcgattgtgtatattctcagcatgag
attaggcttcattctgaaagaacggatttttctagggtttgagtttcggcatctttcagt
gatgtgaagttggtgtttgctattgcgtcttagtttctgaactattgctgcggtatagtt
tgataatctatgttagggtttcctcggaacagggaattagggttttactattctgtggta
tttgatgatgaattgtgagctacaactgaagaaaaagtttacatttttatgtagaaactt
tagttctgattggtttcgtgacatttgatatgtattgttgcagaacggacttgctttgac
acagcagaccgtaaatcggg
>transcript:AT1G15720.1 + Up_Stream_Len 2000
atcaaaataaaattatggacaaataaaaactctagtccttcagaaaaagaaatattgtaa
atgcgggttactaaatacaaatgaatacaaaacagtacctacaaatttccttggaacgac
ttttaattttgacctcaatttttatttccattatggtccttgtgttgttgtgtatttag
cattttgtttttcttgggtcaatcagcatcaccacgtttaggccacgacataaaattgat

ccgtttatttttgaggctcgctcgcatcttcttcctcctttggcgcaaccgcttcttcct
cttctattctcacagatttctctaagctcccaactttggagctaaaactcaatcaacact
actcacaatgctactccatttctctccggcgaaacccctcatttctccacccaatctccg
ccgcaattcacccacattcctcatttccccgccgcgatctcttcgaattcgagcaatcga
cgccgcccaaatcttcgattacgaaacccaactcaaatccgagtaccgcaaatcctctgc
tctcaaaatcgccgtcttgggtttcggcaatttcggccaattcctctccaaaaccctaat
tcgacacggccacgatctaatcactcactcccgctccgattactccgacgccgcaaactc
aatcggagctcgtttcttcgataaccctcacgatctctgtgaacaacatcccgacgttgt
cctcctctgtacctcaatcctctccacagaatcagtcctcagatcattccctttccaacg
tctccgtcgtagcacactcttcgtcgatgttctctccgttaaggaattcccaaaagccct
cttcattaaataccttcctaaggagtttgacattctctgtactcatccaatgtttggacc
tgagagtggtaagcattcttggtctggcttgccctttgtctacgataaggtgagaatcgg
agacgcagcttcaagacaagagaggtgtgagaagtttctaagaatttttgagaatgaagg
ttgcaagatggttgaaatgagctgtgagaagcatgattattacgcagctggatcgcaatt
cgtgacgcatactatgggaagggttttggagaaatatggagttgagtcttcgccgattaa
caccaaaggttatgagacgttgttggatttggtggagaacacatcgagtgatagctttga
gcttttctacggtttgtttatgtataatccgaatgctcttgaacagttggagagattgga
tatggcttttgagtctgttaagaaggagctgtttgggagattacatcagcaatacaggaa
gcaaatgtttggtggggaggttcaatcgcccaagaaaactgagcagaaattgctcaatga
tggtggtgttgttcctatgaatgatatatcatcatcatcatcatcatcatcatcatcatc
ttaagatcacaagtttgattggattatgctagattcaaaaataaaaagttgtgactttt
tggaaaacgtcaagagtggaaaataaaaaagggattttggtgtgttcatatgtatgttgg
ggtctcttttggcttggtatcaataaacttgtcttttttctctaaatgtatctgtggaa
atgcttatccctctggtcgccggaaacaaatacggacaaattgtctccttctccggccat
atttgtattggatcaacattgtattgctcaaaagtcattactgtttttctctgtctccaa
tgatctcgggtcacccacccgattcggatcaggacaacatcgtttcgttatgggccgatt
atataatgggccgcttgaatgtctcgtcttatttaattaaaagaaaaaaacacattttaa
atttgaatgattcacacgtgtgttgttgccaccatagttggtcggagcttttcctcttcc
caaaattgtaaccctaaccacgcaccgacggcgaaaactgatcgcgggttttcgcatctt
ttacttgcaaagtcttggaa
>transcript:AT5G58340.1 + Up_Stream_Len 2000
gttacaagagactttatagcatcaacaatggaaacagcagtagaagcagcagaagatcga
ccccatttctgaattaataacccacctctctgtaaaacaaaacgcaaacttactcagcaa
aacattatatctcattgcagaaatcatttcattatcagaggaatcgccatcttaccttct
gcacactctcagtgaatccctcttctaaccatttgtgatctgtaataacctccttcacag
gcaggccattaattctggcatttaagaagtctggcacctaattttcctctaaaagattag
agaaacatcccaagataaatgtagagaatagatgacactagagtaagataaggcaaacct
gagtcgtggagtgatttccccatatggtcatattagacactttgtcatagaaaacaccgg
ctttaagagcaagctacaaaatggaaattgtcaatcatatgtatgcaaatatattgcata
caatcacatataggtgtgaaatctagtacctggcatttggcacgattttcgtctaacctc
gtgagggcatggaagttctttgcaggaatgttgggagcatttttaagacaaatcaaggca
ctgaaagaacacaaaatgaggtaaagtatacttccaatgtttgtcactgattacgatatg
aagagggttgaattaaggaatgtagcttacttggtgttgcaagggtttcccactacaaga
accttgacgttaggagaggcagctttgttcagagctttgccctgctcagcaaagatttgg

ccattgatgtccaacaagtcagcacgttccattccagggcctcgaggttttgctccaatc
agaatagcccactccacatcttggaacacttcatttggatctgttcctatatcaacttct
ctcaacaatgggaacaatgaatcctccagttccattgcaacaccttcaagagcttgaatt
gatctctctgatcctagcagtttcaatgcaatgggttgatctggaccaaatacttcccct
gaagcaagctacacacccggaaaaccaaagatacataaaataagaccaattattggtaac
cttatcctataaaacagtaacttaaaggattaacttactttgaagagaagatggttagaa
atcattcctgcagcacctgaaactgcaatattaattaacttcttccatgatcttgtctct
tcttcctggcaaacacacacataatactattatatccatgtaaagcagaggattatctca
attctcaagtctcaaagataagattcttttatagaaaaatagtaagagggaatagtaaga
tacagctttaagatcataggtgaggcagaacactccataacactctttcttcgtcttcac
caatccattttcttgcacagcaacaggagcttggctacttttatcatcaattcacaaaag
catctaagatcagataaagttgatttatttaagagagagaagaaacgattaggagcttaaag
agacttacttttgagaaacggagcaagagattttggagttgggagttgttgtgtggagag
gtggaagaagaagatggcgaaagtggtttgaaaggtgcaattttgaagagagacgaagcc
gagacgaagagttgagaaaaggcgacgtcgttttgggggttgagagctctgccatggcca
ttatcgcacagacacaaactgtgaaagtgagatttcagaactggaactatggagagagag
cgcgccaagcgacagagtagtaagaaagaggattggggaatctatggataaggctcagct
cttgacacttgtcactcttttagcatcctcaccgtttatcttcttttgacttttaggccc
agttaaggcccaataacagaatatacctgaaaccgttcgaatttgatcactgatcggaga
ttttcgtcttcgccggcgacattgcatccgctgaccagagaattaaaaaagttgcgactt
tgatcgcgaaagtttcaata
>transcript:AT1G17520.1 - Up_Stream_Len 2000
gtagcacataaaaaacttttctacgaaactacaaaccgaaatttgagagtatagtcttct
ttgaataaaaaatggtttgacagcattagctgcaaagaagatgcaaacagtcgcacacaa
aaaaaaagttcttcttaaaaccagaattaagggaatacctgtcggagctgcaccagcaat
gttagctgacagggcactttgtcaatttgggactatacgcgcttaggctgaacacttaga
ctcgagacttaagagacaaaagcagaagttcacaacagatccacattacatcatggaatt
ttttgtttaagtcgttacctggagaaattccagagatgacgatgagagacaaaaagcttc
aagagaaactccagagaggtaaaaaactgattacaactagggatcaagtgtgatactacc
acgttccggatcactgaataatcacaaacaagaaaagaaaacagctagaaaagccaagaa
gcattaaaaaaatcttagaaaagaaagtagagattagatggccaactattaacaaatgag
ccctcaacatacatagcagacacatagtcatttctctttacataatcacataatcataca
gtctaaactatttctctttacaaaatgataaaagatagattgaaagccatttcacctctg
agtcattttgatatcaacaccaacatacacgctttctggacaagtgaaaaataagcacaa
catataacaaaagctagcatgatcataagcaataactcatggagattcgaatatcaaaag
gtaaaacctttattattgttattattatatctataaagaaaggttcgcaggttaacatac
gctaaaatgtaaaccagaattctaccctagaaacccgaaaaagctttgatttttatgatt
ttcccatataggtgggctctaaagatcactccggttcttcatatgtgagcataccgcttg
aaaacctgcttccccgccacaactgcaccagccgcaattccgccaaaagcaccagccaca
gccgcagatctcactcctcgtgctgccctaaagaccgctccagttcccagaccagcgaca
acactggtccaaacatcgtccttatcagttaccgccaccacaccactctcaatccctgcg
tagatcaacccaacaatcccaaccctattaccccaagtacgacctgcctgaccagacgaa
ttcaaaatcctgttgattttcagcttcgtcgtgtctccattttcgaaactcttgatacca
gagaagatcccagctgaagcaccggctacagagccggctagataacccgtccctgtgtag

aaagtgagattttctccccatgatcgacgctgcttgagagactcttccgtgaatagaaat
tccggagaagtcggaagcttgtatagatactgagatttgatcgggacctggtagttctgg
taaggatggtaaagacgggtgttttcgtcggattcatgatcggagctacgattgatcgcc
attagaagaagacgggagacgttttaggaacggtggcgacggagaaagagacgacggagt
cagagaacctccagcttatgatgtggtacagaataagaaaggcgaaatgttttagggtat
gtaagattttgtgactaattcgaatttgaccccttatgttttattttgaataatttaacc
caagttctttcccgactttacgtttttcattttagaccctataatgtttgacgttttac
ataatatggtacttaaattgtaaatcctaccataacaacctctttgtagattaatctcat
tacatataaaagaaaaagaaaaagattttgtttggatttgggaagaagctttgaattaaa
aaatagaagcaatagtttttgggggacaagcccggccgacgaatcggcggcgagacgttc
tttgtcaccgattgacatattcaaaaacgacgaagctaggtaaaggaacggagagaagag
tcgataatagaagcgtagag
>transcript:AT1G72740.1 - Up_Stream_Len 2000
aaaattaatagctaagagcagagaacagtccagagaaatagaaccagggggatataatag
gtaacaaaataacaccatcaaatatcaggttagtactcaatgagataaacagaggtatac
aattatgtgaacacactacaccatcccaattaacaaaatgataagaaaaaacttgagaga
agagcttcattttcaaatgtaatacaacacatcagtttctcaacgaatcaagaaagagtt
aacattgatcaaaaacagaaacataaaacactaactagaacaaacacactcatgacaaat
taaattaccaatcggcaaaacatttaatcagtatgaatataagaaaaaggtttgcaggtt
attcttcaaagtgaaactgcattatgacttgacatacagacttgttgtacctctagaaac
caaataaagttttcatttttatgattccaagacataccaaacctatcaaattctcaccat
ttcccctatcaaaaatttcaacaacgacccgaaaaagagtctgccaattagactcttgca
gtcttcaaatgggcacataccgcttcacaatttgcttcccagctacaacagctccagccg
caagtccaccaagagcaccagctacagctgcagatctcactcctctcgccgctctacaaa
cagctccggttccaagaccagccaccacactggtccaaacatcatctctatccgtggcag
ccacaataccactctcgatccctgcgtaaaccaatccaatgataccaatccgattacccc
aagttcgaccagtttgaccagaagagttcaagatcctgttgattttgagcttagtagtgt
cgccagattcgaagcttttgactccagtgatgactccaacagaagctccggcaacggagc
caccgaggtaagctgttccggtgtagaaagtgagattctcaccccaagatctacgttgcc
ttaaagcctcctccgtgaagagaaactcaggggaggtaggaagcttgtaaaggtactgag
atttgttgattgggacttcgtagttttggtaaggattgtaaagtcttgtgttttcgtctg
acccatgatcggatctgttattagccgccatcgatggaagagaaaagtctaggttttaag
gattttggtggtggctagagagagagagacgacggagaggaaagagagagagagaaacgt
gtgatgatgtggttgtgttgtgtctctctgaatgtttggtttggttgccgaaatgagaaa
tgaaaccaaactaaactaaaaatctatgtttagggtttttagggaaagactcagtccagt
tgtttccagatacgcgcagtaccagattcgcttaatactcgcgtttacgtttatttcgaa
cttaaccccatttattttattttctctatttaaacccaagttggttttctcttaccacca
cacactgtatttgctgatatttcgaatgtgaccccataagtttaatttttacaaaattaa
cctcttcaacattggacttttcaaagtcatacttaattactttgcaaaattgcaatccat
cgtgacaaccaaacatcctcgaattttcaggtgtttctcttataatgtcagaaatatttt
tacgacgaacgaacctaattttatgaatttttttgtcatcagtttttatttagagaact
aaacttcaaataaaataattaaggattcaaatacaaagcttatttgtttttaatttaagt
caaaaggccaaaaaaaaaaaattcgatttaatttcgagagcgtaaatagaagacaaaataa
aaagcataaattcggaagtaaataatcgaaaaagttttgcaaactggaaacctcctcccg

cagtcacacaactgcgcagagccaaaaataaacgaaacctccggcgaaagctacggcgaa
gagactgcgtctaactccgtttacacgccgattgtcttggagagagagagagagcacgag
gtaaatcacgcaagggttag
>transcript:AT1G06910.1 + Up_Stream_Len 2000
cactgaagttgttgcaggcgacatgcatcattttggagggcctggttaaattctacaaat
ttggattttgttagcaagacacttttaactatatagaaagatttatatatctgaatgttg
attggagatggatcttttgataatttcaaacaaagaaataagagtacgaaacaagcaaga
taaacctgaatcaacggccaggacttcccgttccatagcttcagtcttcatgagcggtgc
aacaaaaaactgagagaatctaataataacgagaaaacagttctaaggtcaaataagcat
tttgaataagaaaaatgaaaaggaaaagattaaacttaacagaaagctcaaatattgcgt
acggaatttttttacaacaactggtataggaaagacaagtaattacataatgacgaccag
gaaggataaaaaactatgcatcttccttacaatgataacaatctctgggcatgcataccg
acataaatatcgactgccttggtaaattttacagcaaaatgacgtttatctagatatgtg
aagtagcagctcaagcaatttttgtaccttttcaaggccccttgaagaaattctctcttg
acttcgaagtgatagcatgtatgctccatttctgtgtatgcgttagaggatccaccatgc
ttagacaagtaactatcatactgcaagaagtcaaaaccggaagaattaaaagatacatca
tgctaacaaaacgaagtccatatccattatatccaagctccgtaaagattcattgttata
tctaatcattctaactcggttagtttcttgattcaaagatagaaaattctaaagtaagca
gaatacttatttcatatgaatctataaataatgccaaaccattgaaaccatagaaaataa
tgcaatgcttacttcattctcatcggggaactcagtgctacccataaaaagcatgtgttc
ttcaccaaacataaacatgagagtgattcaattagtaaaaaaaatcagaagaagtgcatt
caaaatgtagttcaaactcatagaaagaagaaaataccaagaaagtgagccaggccttgt
gcctccggaggatccaagaagcttcccattgaaacacacatagctgcagcagcctgagac
acacaacacaaaatatactagtttaaaaatcaaaacccgatacaagaagaaaacagagat
atagcttttaattgaaaacctttttggtctgatgatcccctttccctttcacttcatctt
catcctcatcctcatcttcttcatctccttccccatcttcctcatcatcttcatcatcat
catcatcttcagagctcccgtcactatcttcttcttcaccgtcctcgtcatcttcatcaa
tttgatccggaacagagccttcggggtaaatatctggatcgtgaatgagcagagcacaca
atccattctccaattcaatcactctgtataacctccgatcgtttggtgacttcaccacta
cattgtccaaagccgagacggatttcattgaagacattgttctcgttttgagaagaagat
agcgactggtgaacaagttctgctgctgctgcagcttcctcttcttaggtcttgcttctc
tgtacccaatgaatccgattccgccgcaaatgccttctgatgatttttcgtttattaaaa
aatgtaaaaagttagaagcagtgagtatcttgccgttttctccctagggatattgaatgt
gagccgtttatagtaaataaaacgacacgcgtttctatttttaaagatggaaacggcaaa
aagttatggttctttcttccatcgcttcgttttcgcctctgtaatcggaagaaaatcgaa
aagtgaaaaattggaaatttgaagtgttacaaacctaattgacctttcctgtgtaaccca
attcgagagacggaatcggcgagtgagaaagtactgatttctctaaaaagcccaaacatt
gcttggttgatttagcaaca
>transcript:AT1G17460.1 + Up_Stream_Len 2000
gtgatgttttaaagctatttgtggaacagcttttacttgtccctttatagatctctttcc
ctcttgggttcataagaacaaaatcctcttccctactttgtacgattcatgactattcag
ctgaatcgtgttgggttcttgtctgaatttgagtgttttaagattggattattactggaa
atgtctgtactaatggaatcttatgtgagctttaaggattagagctctaaaatcaagata
gggtcaggtcaacgcatagagaataaattgtgttattgtaactacttttctagtcaacgt

tttctgttaaattgaatccctttcttggctttgtttccatgtttttgacatgattgtatt
gaaacaggagtaaaggatcaatcaagagtttgatcttgttagaatagccgataatggaag
gagatgtgttgtcaggatttggagacagacacaatatggatgggaaattgttgcagagtt
tccagaagagctttgtggatgttcaagacattttggaccaaaaccgattattgatcaacg
agatcaatcagaaccatgaatcgaaacaaccagataacttgggtcgaaacgtggggttga
tcaaagagctcaacaacaatatcagaagagtagctagcctttatggtgatctctctcatt
cttttgcaagatcagtggatgcttcatcagaaggtgaatctagtggaactttgaaatctg
atgggaaagctaaccagaagagatttagatccggttagtattgtaaccaagaggtccaag
accatcaaagttgattatcatagattcatattcagattcagagctaattgattgaggaga
aaggagaaaggagagttttacaaatgtaatatctctattgaaatgattaagagtctttac
aagaaacttgttgtaaagtttacacatttcggtttaaggattcttctgtttttcttacct
ttggtctccagtgttatcattgcatgtttgtttgttgttgtatagcctgtgcgagtttta
catgagtgcagtattttgaactctagactaagacgacacgtattcgaatcctgaagtgta
agacggcacgcagttgtggtgcacgagagtcgggacggaacgtagttggctctttttcgg
cagatatttcgctattcgctgacgtggcaacctctaggagcttcccgcataactatacgg
atcccaattggcattctgaaatcgagtgaataaaaagctttggaatccggacctgtatgg
cactgtattagggaaaaaaaaagccgaaagcgagccaaaacaaaaaaaaataaagcagaa
caaaaaagtttgcaaatatccaaggtttttagcttagtcaagaagcctctcctccgagat
ttctcgccgatgaaactacgagctcactggaaagtctgatttttttcttctccgttcccg
ttctactttctctggtaactctcttgtgtacttgttgctcggttaactgctttcctcgtg
acttttcatatttctatcctgaaaattaagtagtgggtttgtttgatgcgagttaaactg
agtggaatctaccaaaaacttgatgtgagaactgcaacacgcaaattccattgaaatttg
ataaatggtagcttaaagttgaaagctttttattattctgaagatgcagtggtgttttt
tattggggatagagtataatcttattaggattattactatatatcttgtgatagagttta
ggtcctatatttgagggattagagatatataggacctaaactctatcacaagatatatag
taataatcctaataagattatactctatccccaatatttttctctctatgaaagggtaaa
gctaggatttttgagtatcagtgattgtattgtgtaagagtttttttgcatgactgaaaaa
tttattaagcatataggtatatttgcttatgcctttttgtatgcttgaagttgcatttgg
taggttaaaatagggtaaca
>transcript:AT1G72650.1 + Up_Stream_Len 2000
ggataactacaaaaatacctgctattggatcggataagattgttatgcaaggtgtccaga
gcaagtcctttcattttgcaaccttatctcaacgttcttctgctgataaagtttgggact
ttcttcataaaaatgtgtgcatgctagtttctagttttgttctctcttttttgtgttat
cttctatgagtctctttattcatagatgttttcgtttgattatatataccttagtcatgt
tatctatttatgatgtagctcgttcttacttgagattgcactagtagtcttgcttaatga
catataaatcttgatttaaactccctctggatattctttcatagagaaaaaaaattctta
tgatgattttattgttgttgttacttgttttctcagcattgatccatctctgctcaatcc
ttacaccaaattttgatggtataagaggattcagtgacaatgcttatattaaaaatggag
tttatagtctctctgaactatctatgtccaagttcttgggagttcggaagcaccttacat
cgttgacatttgttctaagttttcctatgacaattcttttcgtttgcacattcagagatc
agtttgagatccactaaagttgatgtggtctgatactcacttcttatcccaacctaatca
ccatgataacttagtgcttttgcttaccgtcctaatggttttgtgtttagtttttttctt
tttcctttcaagtttccattgttgcaggagaaacaggtctactcagacatgcaacttcca
ttcatgatgcagcaaaatgatcattgttgcaagagaagagaagtccacaatgatgtctca

aggatcaaggacattatttagcaacaaagaaatgttgatttatgttttcaactatcatgt
actaagtaataattcaaactttgtgaaacatctaacaatttgagagcaaaaaaattacaa
attaacatatgagtgtcaaattcacagacggcgtgcaatcctgatctagatagagcagac
gacacggcgtgtcttgcacacgtgacctaaaacggaacgtagtttactctgttttggcat
attaagctatttactgacgtggcaatgcttcgatatcggaccaaagctgaataggtatat
acggattccaaatcacaaaataaagttatttttgagttaataaaacgctttggagtccgg
gacctgttaattttgctctaagcactggaaaaaaaaaaaagcctaaagggagccaaacata
aaagcagtaccaaaaatttccacttttctttaaccttttttgcttgtctaagaaacttct
cctctgaaatcatcgtagattaatctgttttcttagacatgattaagtctccgacgcttt
gaggtaaccggaaagtgtaatcaatcgcttgttgctgtatttgtcactctccggcgacaa
aacttgatttccggtggtaagcaataaaaattcaacctttttttaattcgtttctacatt
tcaagctttgattttgttctttcttaatatgttgttgttgtatttctgtgttgtattcgt
aattcgatctaaaattgtcaaattgattgtgagaatgcaaggtcttagtttgtcaattgc
gaaacttgtgaattctttggaatttcaatttttaatctttcttaattttgtatacttag
tgttatacttaatattcaaagggctttacttttgtttgataatagatacttctcactttg
gggctgctttgttttcgtctgatggtgttatttgtatgagaatttgctttgtgttcaggt
ttttggtgtgaaattgtacagagatgctgtcaaatagtaaaagaattgtttttttctttg
gtaaagttatatgtagtactgaaacatgctttggtagattggttttttagtacttgaag
aatatattgagcatatatcaatttgtttgctttttctctgtttgggattgcagatagtgt
gttcctaagttagatttgcg
>transcript:AT1G07540.1 - Up_Stream_Len 2000
aatcatcagcggccaagatgtgattgatttatcccgtgaaaattgtaacatagtggaatg
gaccagttttatactcgaaaatggtgatattgaaagtatagtggatccaaacctccatca
agattatgacacaagttctgcatggaaagttgttgaattggcaatgtcatgtgtgaaccg
tacttccaaagagagaccaaacatgtctcaggtcgttcatgtgctaaacgaatgtttgga
aacatgtgaaaagtggagaaaaagccaagaggtggacttaagcagtcctctggaactgag
catagttgtagacaccgaaataaacccaaaggcacgttagtcattattctcatggttggg
tgtataatgatcagtctgtataatttgtattatgaagcgtctattgcacaatttgggtgt
taagtctgtatgttgaacgtgttgatacttaatagcttcttcttgtgctatcgaaagttg
aaagtactcttttaattatacaagaagaatatccactcatacagatatttgtgattttat
tcggtttaaagaatctgtttatgcggttcggtttttcggtgggcttggaaattcactgg
gtttttattttgtgggccgaaagtgtaggctgtggtgtagaaatgttatttttgggctg
taggaactttgtaagcccaagaagaaatcctaatttcatcttcttgaaacacgtgttctt
tgtccttcgcgatttcgatgctaccgacagagaagaagaagaagaagacgagcttcactt
cggtatttactctcagatccatcgttccgttcaaagtttccagcatttagaaggactatt
caattcggattctcggcaggttgggaaagaactgaagaaccgttcggttcagtgatgcca
aagtatatttgaagcattgccgatcggttgatagctgcaaaccgaattgatcgatcggtt
aggttaattaggtttaggttaaagtattttgcatcagaaaatagatagggttctcaatga
aaacaaatttgttattataaaataaaatagaagaagaaaaactgaaaaagacaaaataaa
ataaacaggagaatgacatggcttggactgaaaggaaaaaaaaaacattacatggcatgt
gtgactcaacgttattgtcgaagtttgtgtgttgtaaaaaaagccggacgatccgacaga
gacaacacgtggccgaatcaggggaagtacgtgagacagtggtggtgacccaagaaggg
agaagaagaacaggtcttcagggtggggtttactggttatagccggttattcgctcttct
tctttttcagacacgtcctctgtgttactactctcgtataagaccaggggtttaagccc

ttgtctccttccgtgctttttcccacattaaggggtttttcgatttcccacaacaaaaa
ataaataataagacagaaactttttgctttgtgtgggttgatgagaaaataagatcata
aacattctgatctcaccttgtttcagaagaaaaggtgagattatttttgaagttttttg
gatctgcaatttgaattgcggtttttatgtttatagttttctagcttgagggtttttg
atgttgtaatttggtttctcacagaagctgaaccgtgtttgtttttcatgaaaatattac
aagaccagaattgttcattatttttcttggtaaattcatgctctgttttcactctacggt
cattaatcaaatgaggctttagatcgtggaaatttcaataacttggattctagctagggt
tttacaaaaaatgatgtctgaaattggaaataatctgctatcacatatacagtatgtgag
attttcttgcaacgtaagaaattttatagataaatgctaatacattgttggactcagtaa
cctaattgtttttctccagggatcttgtaatccgtgagaatgccttcgttttgttgaatg
attgaaatgttcttcaaatc
>transcript:AT3G53790.1 + Up_Stream_Len 2000
tcctctacctcgttcttcataagaacttcgactctgagtcaacgatcgttgttgcccaga
tacagaagaggacgactctaaatccattggctgtatattgttgttgttgctcctctctct
ctctcgagttttcccccaaatcggagctgtttctgaatctttctcacccattgaagcaat
taaaaatccgagctttgaacagaattcgagctttgaacaaaaattggagagatttgagatg
tgtgtagggagaaattaggtttcgtgtttagctctttatgggagtttttaatcgggtgtt
ggactttgggagctaagtgcaaagttcaatctatacaacaataagcaaattttggttgga
gtttttgagtctgaaaacaagaaaaaaaaggaaagagttaaataagtcaatggtctttacc
aaatatgcatcacacatggttcctaaaaaacaatcatcaaaacaaaaaaaaggaaaagtta
tgttatttcgtgtctcaaaattgtttacaaactaatttataaacaaaagttttagtaaga
aaaataaataaattttgtgatcataaggataatagttatttattaccctaaataaaagtg
gccctaaaaagaaaaaagagaaccgagtcttttgcttaatttgcttatcatccgaattag
gactgacttgttaagtagttcaaccgctccgtcgtccaggggtttctttttattgtaatt
gactcgtctacttgtatctttagtacgtaagcatcgagctttgacatcgacgcttttggt
cgtgctttctatcggtgacctactctttctctatattgtttatgtacctacataccattt
ggcatttggaatccgaattgtctactaaaaaatccaacctatcattatcagtttgatttg
aaacagttaaaacataaatagttaatgaaagtgacgaagttgaagctttcttaagaatga
ttattgttttatggttttcgctagatatgagaattaactaccaataatatatataacctt
agattgctttggcaactttaaatttgttgaccatatattgtcgttaacttgggagctacg
ttagcgaaaaagattaaatccaaagttttacttaaaattaagtatcattcaacattaatt
ttaaacattatcaatttaatttattgttctgcaatcacattttccattcaaattcatttg
acttgatttctattatatatacaccttattcaacacgtttctttaactgattgccagtta
catcctaaaatgcttccgaatggaacaaaatatgtactccattttgccacgttatttatc
caaaaagtactcgcaaagaaattcctccacaacaagaaaatattagtgaatgagaagaga
tttatccatgaataatgtagagtcgtccacgtgttaaaagaaggatcgatgttgagaagt
catgggaacgaagaactggtccaaactgttcggaggagggttttaggtctaaaagctaaa
aaaaacgttggttagttttagcccccaaaaaaaaaacatgtctttagagaaaaatataatt
cagaaaacgttttaaatttactgataatttcagactactcagatgttgacatgtgttgtt
aaaattgagttcaaattttcttaaaacattggagttttttgtaaagaatttttgtggtt
tcatggggtttttgttcagacacaaacccaactttaaaggcggatgtttttatacggctc
atatctttctatatattttacttccacacaaacaaattccaacataaatctcacttttct
ttctcgcacaaaatcaaaatcgtttcagataatcaaaaggtttgtgaatttgtatgagat
ttgtgacgaatgtttgtttttccaattatcctcatcatttttgtgtctcatatatcggat

atatgaatgtttgataaaccgagattgcgcaggctcaggcttaacgtaagcaatttgcag
ttgtgtgtgttgcaaggaac
>transcript:AT2G30424.1 - Up_Stream_Len 2000
acatttaaaatcattggaccactttagagacatattgtggaccaagttcttaaatcgatc
aaacatctaccaatcaacaagccatgtatttattcgaacatgcatgtctttttgtttgtt
tttctagttgatacttattgatctggcttttaaatttggcatcactcgtatatatatata
tatatatatatatatctcgtagatacgttattgtcttatagatataaggtatgtcttt
gtcacagggcaagctctaagaacgttagttgaagtgacatactgcaaccatgtctgatgt
ttcaaatttctttccaagctctaaaccaagaaaaagaaaataacttatcaaaagaacaaa
cttgatttagcaagagcatcaatacacatgttacaagtacgaagctggaacgagacggaa
cactgatcaaaagagatcgttgaaattagaaaccaaatcatataaatgaacttacttcag
ccccagcgatgacgcgcactactcttttgcccatctaactatctaaataggcctagtcga
ggataaacctttggttcttttcgttagttaataggcctaggatttgtcttgtactaatta
aatgttgtataataatgtatacatatatatatatatggttctttatagtttcacgctgag
acatgaacattaactgagacaactttaaaccttgaatataattgagcttgttatacgtgt
cagtttcttattacatcaactgaatttatttatcactgagacatttattgactccagtca
taaatagtgcgtatatgtataattgtgtaaaaaaaggtatgtaaaatgtatgttgagaaac
aaaaaaaggtaatatgtgtagaatgctaaaaatgaaaacaaagtacaaaaaaatcagaaatt
tcattggtgtggcatagtggttactggctcggatctactaggacgagtacgatttcggcc
cacgtacagatctaatatcaccgcaccaaattaacagattgttggagtttgtccaatttt
caagaagtagattcaaacaatactttcagaaacggaacaaaagatctaaacgatattgga
aaagtctactgttgtaactttcctcacaggaccacatccccatctccgtcagtagaagaa
attccaaaataataaaaataaaaactgagaataattgatatgtcaaataaattagaacta
gcgatatactgcagtgaatagtagattaaactatgttagaatttggttgtttctacataa
aaccccaaagactagtaaattagtcatgacgctttagtggtgaacatttttctacattta
ggaaagatcgaaataccaccattttcactgtgaccacaataacgtcttagatgatagaaa
aattgggcaagttttcgcttaaaaataatttagtactcggattcaaatacatgcgaattc
caaaaaaaacattctatctccatcatttttcttttggttgtgtaattgtgtatgagtgta
tccacaattcgggttcaacgcggagctggatttttttaatatctttttgtatccataacat
gtggtttttttagataagtctttatgattatacccattgtattttcctaatgtatttgttg
tgttcacataagactttacccttaagtggaacccacaggacgaaaattctccgggacatt
atatatgtgtatttaagagcaaatcatgtatccatagttataaattttagtgtttagtat
ttgataaatattactccaatttacaattatatggtggatattcagatattttatccagtt
gtatcaagagcaagtctactggtccagtagtcctcattacggtagcttgggacctccttt
tctatatctctctctttactcttcgtcacaagttttctatatagttttctctacctcaca
tctacttttttttcattgcattctccaactccaaaatcatcagttgtatattatttgtcc
tctttcactttcaaatacca
>transcript:AT2G30432.1 - Up_Stream_Len 2000
agtactgtataacttcaaataattttacaatacttctatatatatatagataaataatga
atttgataaaaaagattcctgtatttctaatattataatttaatatgtatcatagatcct
tatctaatgtcaaatatagttaaaacttaaaagtagaaccacagtgcaaagttatattat
atacatatgtaaattcaaatatagtatgcttgatacattaatgttaattttaacttttga
taagtactttttttataggtaggtaataaatacaaattaggtcatatgtgtggatacaaat
ggttcttattgttaggtttgagaaatcaaatcgagggcgtaaaaaaaaaagtaacatgaat

catatgtaagtttgtcagtgggggatgttaacatgaatcatatgtaagtttgccagtggg
ggaggttaacacatgaatcatgtaccgttcattgggtgtggtttaatacctagtcaagtc
gttcgtacatgacatttataatcatcgtaccacattaaataattgtcgacaaatcttggt
ttcaactttcaacaatctaggggtttttcttcttttttttttcttttctttcaggctta
tatttagattgggttttaagtggtggtcaacttactaatttaaatacgaaaactcttctg
ttatttagtgttttaaaaccataaaactatttgctatgataaaattgttagaatacactc
caatatagatgaattttattttacaatttttttgccaaaatcaaaattgttagattttt
tgtagactaaaaaaaccattgtgtgattgtgtctacttccgatttgagcggatatacaga
cttagtcgaggaactcgatgttaagccttgcttcttttgttgattaaataaaaacttctt
ggactagttggattagataacttgttgtgtatacttatatatgtgatgtccttcacttaa
atgtaacacgttatagtactgaaaacataataaaatgaatcagttgcgacttaattcata
aaccatgttagattttggatgtttccatacaacaacaaaaaaaaatttaaggattcaagca
acgtgagtcatgacgctttagcaatgaattgttttcctttctgtgagtgagtgaaagatc
gaaaattttgtggttgattatttagagcataaattattcctctcaagtattaaactagtg
actttgacccacacattagttttcaaaactattagaccacccatttggtttcaatatttt
caatgttttataggtgataaagttttcgcaagttttcgcttagaagattatgagtactcg
aattcaaatgcatgcaaattccaaaacaattattcgtcctttccttctgtatgtcacata
ttttgttgtgtatgtcaacaactaggctttttgtttggtatctataattcgttcctttt
tttagtttgccggatcagaatgtattttatgattcatatcttctacaaaagagccggata
tactatatagtttcctagtttatgttaaaatttatgtatccactttgttccatcaaagtt
attaaaaagataaaagttgcaagataagaaaaaataactacatacaaattgcaaacaaaa
aaataatattttataataaattcctcattttgaatacataataatatatcgtttataaat
acaacatatatcatatgcatgtattattattacaaagtggaatgatatccataatttaaa
atgtttgtatttgtgatctgctttgcttctgaaaattagttttagcatttaatttattca
gtaagttatggtgtacctatattttgtaagttaaaggttatgtggccaagtaggagagtt
ggggacctctctttgtgtctctacatagttttgtaaacctcacatctctttcttcgcttg
cattctccaaactccaattttttttgtttctctcaataatatttgtgttcatactgtttc
gctgtttccaatacatacta
>transcript:AT2G30420.1 - Up_Stream_Len 2000
ttaaggttaattccacccacatgctatcaccataattaacaaaccactctcaatcaatac
tatcaaatcacataatctcatgcttaagagttaagacacctaaatcgttcatacaatcat
gctcacattcacatatcatatacattcaatttcatgcacatgttcgtatgcactattgtt
cgtaaccacttagctatctcaaccatccatcatttcactatcttaaccatcgagccctca
gatataatgcttggcatctctccatctcttagtcatcttgaccctcaaacttatctgatt
ctcaagatagtgagagctcaggactcataatataggagttttagattttgctcaccatat
tgggccattaagccctgtttcctttttagtccaatcagatcctataattccagtgtata
ccaagcttcctgatctttaaacacttttaggcaaatataatttcttgtcctttcttactt
agaaccattcttcacatgagaaccatcatttgtgtatatatattaataactttcaaaacc
ttaataatgatcacgacataaaatctttataatgagagcgggtcattacaggtgaactcc
tttgtccgtcatatcagccgtcttagaagctcgtatgtgcccaatactctcaggaccagc
cctgagctcgtatgcattaattactgatctggttttaaaatttgtatatatacgtacata
gccatatgggatacttcaaatgtatgattttgtctcgacagcttttacaagtttagtcga
agtgaccagacaacagctgcaatccgaaagcatgtttcaatgaagtcgttctagtccatg
actgaaaatttgttatactgaatgttgtcgataactttccttttgttaacgaatgcttgt

cgataaatttatatatatccagtattatgatcacataatgataaattagaggaatttcat
atttttgaattggtcttagctaatacgtaccacgtaggatcaattttctcctaacctact
tctttaatatatagtgggtaaaatcataaatggttcattctgatttcacgctgagaaaat
gatatgctcattatataagtaccgatttctctaagacaactttcaatctgtcaggaaaag
aaattgatacgtccgtttcttacagttactgcatttattatcactgagacgataattgag
ttctgtagatgcataaataggaattatctgtagggcatgcatatgtaaaattagacacac
acaaaaaaatctaaagagaaaaaagcacaagaaaacaaaagttttgttggattttgctag
tattatttacgacccacaaatctatatcacaacacgaaattaacggtttatggactttttt
ttgtccaattttcacgaagtcgaaaatttcggggaaaaaatttcacctaatgttattgga
gaactgtaaaataaaattatagattatgtacatccacattccacagccatctccgtccgt
aaaaatttctaaaatataaaaagaaaaactgtgattttaaattagaacttgtaggattac
gtgttcataatatttattgagaagtgtatttgcatgtgatgtttttcactcgattttaat
acgttactaggcaaaaaaataaaactagcacgccattttagtgactgttaaaatgttctg
ttataggtcagaaacaaaatctgatcaaaatttattgtgagcattatgaactttttggaa
tagtaaaaaacaaatcgaacgtatcacgaaattatgttttgacgtattcgatcgggatat
ataaaaacaaaaatgtgaattataaatttaggaatatatacgtagaaattccaataaaaa
ataaatcacctatctctctctctctcttttatataactatttttttccctcacatcttt
tctatatcttgcattctccaaactctacattttcagtttctctgtaaatttatctaatat
tcttctattttccagtagtt
>transcript:AT5G53200.1 + Up_Stream_Len 2000
taatcacatctttgtccttttgtaccagaattttagacattagtttagctatgcaaacca
atttcgttcttttagctccaaactaaagacacattttcattgatgcattcttcgatatcg
aattgatttgtttatcaaaagtttatgaaaatcatttatattgctatattgtatttaagc
ttcgagtaaagagatattttaccaactaggcttcctattaaaaaaattccaaatctatca
cataatacacagtttctatgcaatgaggatgatgcgatatacatcacatgactcgcctca
cacgatgtctttcgccgtagactacagatttcttccgtttaatcttttatccaattatga
attatctggtagggtactttatattttgaaaatctctttcgtgagaatatgatagtaatt
gtatactttcttacaaaatttattgagaagcgtactttatatatgatgtttctcactcga
attaaatacgttagtttaatgggtcgattcagatttataatagatttaaatatttcgaag
aaaacgcaaatcagggattttggttgtatcatgtgtgagagattgaatttctccagaagc
actcattaaatcgcgtgtgtaaggagttgccatacagcgtcgtatgtaatgtcttagtca
ctgttctagaattaaaacatatgtcaaatgtagaattaaaaaacaggggttaaaatgtta
aattattcggagaagtggaattattatattaaaaacattgtgtgattttgggataaaatc
tgaatcatgtttttttattgtgccaaaaaatttctgtttcaacctcttcgtggagatgaaa
gaaaagtcaatcgggaatgagacttcttgtataacgaatgaaaatatgaacatgttgatt
aatgaattaattaacatacgtactatattaagtgggaatattaggtataggccttcgatt
ttccgttttgtttggtttttttcgattttaataatatttctctaaattaaaataaatttta
gtttggtttatttaaaatattaataaaataatataaataactatctaattaatatattaa
aaagtattttctatatatattgatataaatattattcccatgttttatattgggtgacgt
tatcttttaagtaaagttgtaagatatctaacttattcctacattataataactatcaaa
ataaaataactaattgtagtgacaatgatgactatcaaaatatgtatgtgttttggctca
aacatagcgtttaataacaaacacaatggacaattttaataaatttgatcgattagattt
tgtttcattttcatgcactagattaaggttcgaattagattcattttagtgtatatagtg
aaactaatttgttactatatggaaaaaaaaaataagtttatttttggttcaagaattttta

ttcggtacggtttggtgtgtatgtccaaccctgaaaaaaaaacttgaaaaactaataacc
taataacaactaattttcttagaaatacaaattggacttgaatacaaaaatggaaagaac
aacaacaaatcaataactaaaacatcgttgaacttgcatctgccaaataaaaatcaataa
atcccattattctaaaaagatagtaggtttagaaaaattgaataaatggaaaaaaaaaat
tgttaggatctacgaatgagaacaaagttagccttcaaaatacttacaaatcccaataaa
agacttcatctccatgtgtatttgagtgtcaacgacaagtctacacaaagggtaagaggt
caacaagaccacacaacacttcttactattagttttgcaaaggccgttcgttggacattt
ccttctctctcctcccctcttcttcttcttgttcgctctataaactctcatctctcacgt
cttttttccttacattctccaaactcaaaatttcatcacattaatttctctctattttt
cttttcttacttcaatagta
>transcript:AT1G01380.1 + Up_Stream_Len 2000
ttatatccctttgaatcattagttacttgaatatcatatataaaatgcttatctatatct
gtttttgttcatatcagtgagaaaagactttgaacttgcacgccggcttggaggaaaag
gcagaccatggtgatagaaaactcactcactattcacatctcttacattgtaagtgagga
tcgaatagttaacaacaagtgtgtcgtaggttgtataaggttattctctccttgtctctt
ggtgatgcatgtccttttattttactaaatgatctgaccaaaaccttcttttgaaacata
actggaaacccgaatttggtttgattccaatatctaggctgtaaaacttctttctctatg
tgtgtttctatctgcttttcgttggatctgattgttttccattgagattccacttgacaa
aaacgtttctttcgttaagttctaacttccaaaggttcatgtatatataatttgaagcct
cagttagaaagggttgtgattgaaagaagaaaaaaaaaagtaagaggaagatttgaaagaa
catgtgttttgggagggctttagtagaaatatctctcagacgagtgacgagtggattccc
aattacaacttgtcaaggcatattaaaaaggttgagatatccttagcacttcaaatttaa
ataattcatattgatttttagtgataattcgcataaaacatttgtgatatcatagtgttg
tcactaaaaaaaacactataaattttcgaaaagctcgaatttcgaatttggtgatattta
tttcattgtcaactcttttaagttgcctctttttcttgggttctctatagtctcttttgt
tttgtttttacatcctttatgatctctctctgtacattcgtaaaaattgaatgcagacc
gaaaaaactaaacagaaacgaggatggcaaaagttggatgcgcccatgaatcatgcaaca
caatggtcccaagttcccaacagattcattcgcaaaaattcagagtagaccgaaagaaaa
aacaaattgtagtattataaaaaaaaagaaagaaagaggatgggtcaagtgaaggcgtca
tgaatcatgcgatggtcccaacaaattaaatgaaatataaaatccacgtgtcatgttcag
ttgcaactgcatagtttgttcagtacgttgctgatcaaccttttctttttaaagttgat
agataaatataataataaaccgaacaaaatttaaaagaagctttgaataaaatttgcgat
gtgaattaatggaggaataaagggtatatcctcgacgttaaattaattttatattcatcc
ctaacaaactttcaactcaacttttgatagttctaacaatatatttcattaaattgacaa
aatttaattcgatttgtaatagattggacttcataaatgataatcaagtgattctcctaa
tgctttttttcttcttgggaaaaatgaatacatatattgactcttttttttcatacatat
ttgtactcaacatagttgatttatatcgacttaattatgatttattcatctgcgtgtaaa
tataaaacatcaagtagtatcattttaaaaaaagcaattagacaagaacatgacggagca
aatgatgtaatgcaacatgtcttatgattctcattggtccttaaacgaaatcactcgagc
attgcacacatactgacatatattggctgccacccataagttgctaaattaattaataag
aataatattagttaaatccgatttaaggaaaacaaagtacgatatacatcatacacgatt
ccttaatgtggaaaccgaccaaaccggtcgttaaactttgcccttataaatacagaatac
acttcatgttcttcccttaaaaatccatcattctctcttcctctctgctctctctttacc
ttaaaccgtaccaaaccgtattgattgataatattgcacggtgcgatcgtaaatctttgt

gtactaaggtgtcttttgag
>transcript:AT4G01060.1 + Up_Stream_Len 2000
aaaaaggttaaatggggtaatgaaaagtgtgacatatacttagcataaaccaatgtgtag
aataatccatgtttcctttattaagtgctatgcgttaatcggcatctataaagtgttgca
ttgatgaacaaagtggatgcctaaactagacgtttaactaaatgtttagaatgaaatctt
catctcatctaaaaagtgttgcattgatgtaaaaagtggatgcccattagttcttggctt
tgaaatgtttttagaatgaaatcttcatcaatctccatatgtggttcaatccactcattt
tatcttttgttaaagatgttcttcaggccaatataatgatgaccatggatggtttgcaac
tcgcatataacacttctttatccgatggttacaagtattacatggctatagatagctttt
gcatgcaacaaattatctatcaaagtttatgcatcctctaaaatatggtcattggcaagc
cactaaacgtatatattgtgacaatgtatgatgatatatttttatgtgttgactccgttt
ttcattaagtaatgaaacatgttgctctagattaccattttaatcgcaaacatatatgtt
gctctaccacgcattgcatcaaatgataagcttgaggacgcttcgacaaaaacatatttc
tccttcttcttactgaaccaaatgacaattgacaaaccccattaataaaatcggttagtg
ttaatgtgtcactcataatattaacttagtaaagaacaagaccacattaattaaatcagg
tgttagtctgagaatatacgtttctcttcctcattccaaacttaaattcggaatttactg
agaatatattgttagcactgaaaaaggttaagttgaaagtttgctagggatggcaattaa
atatagtcttgccttggggatattcccttcgtggacttgtaagtttatttataggtcctc
ttatgtatatatagatgatctaacgatcgatatactatgaaaaaagttgttactagattt
tattgcaggtaatagtgttgaataacccgaaccaataaagcagttgtaacgaacacacga
cacgttgcttactgcgaggaccactttgtttttgttttttttggctttaagccaattta
ggaccaaatttgcatgattgaggatgcaagtatccaacccattttcatctttcgtagtga
cactcatttacttttgtgatggacacgttatagtatatcttaaatattaaagagacatga
ttgggggatcattgttttaatttaaataatgtagattctattcttttcatggtattaatc
caatttatagaaagttatgtgttattagcaattaagctaaatgatgaaaacaatcagttt
agtgaaacaaactcgccgagaaaacatgaatggttgaaaatattattgtgttttacaaac
gtacacgaggacaatagttttgtaagtttttcttaggcattgaaaaatgtttgatacaaa
aagtaatgttaaaataattaaaaatgattttgtcttaatatatccaaaatttcaatctat
tatgaacaaagggagtataatttctgattgaatgaactggaatagcaatcagaaaagctt
tgaaaacaattgttgttgattattaatgatcttaattaacggcatgtatcaatatttata
caacttatgttccagtccaagccatcacaacggagtaaatgaagtcacgggtacttgtgg
tttttattggttgcaaacttgcaacttgcaaagatagctaacaataattaatataattaa
tgagaacaaaaccaatttagtaaattaaaatcctttaacatagaaaccgaccaaacccgt
tggaccgttggttacttgatttggttagttgctataaatagaaatgatggttcgtgtgca
accttcaaaatacgaccactctctcagagtactctcttagtttctttcttcttcttcttt
gtaatacggtgccgtttgac
>transcript:AT2G46410.1 - Up_Stream_Len 2000
tccattgactacacgacacgtgtgataattaccggagaaattaacgtcggggaaactggc
cgccgcgtggcatacagatgctgacaaattcagacgggaacggtggtagacacgtggcaa
taaaagacaggaggagcgtggagggtgcttgaccatttatcgccttggaatttgagactt
ttaaggggcaaaaacggtagctcacaagtcaacacttttgataatctttttgccgtttcg
tataattcaattcaaaagttaatgttgataaccacgtaaacacttcttaaaccgaccaaa
taatataattaaagatttgattgattgataagcttttgtgttttctttttctgttgttgt
gcgtgtcaacgtgttgtactctccgtaaacattcatgggccttttcaagtaactgctcaa

gctggccgatagcaatttaaaataatcaataagcccatattgtttttgaataggcccatg
gcttattagggcaacatataacgctttaaccacttcgtcatgaaaaaacccatacaacaa
catgcagcaactaacattcaacataactgttttcattagcatataatgtacgaaaacatg
caacttatctctataaactgttagatcatgtacttaaaaatgcatagattaaacgttttc
aatttatcataacatgtaccacacaaaaaaaagtagctagagcagctattcatcttataga
atatatagacgtatctcaccttcgcactgcagatcttatacaaatctttttaagaaaatg
aaaaaagggggggctaatctcatatattcttcgaagtctttttgggttattaaatagcttt
aaccaccacatgtcataaataattgttgttagatacttgtctgattcatgcctttttttt
tgcgagtcaaagctttgtatacaacaaatgtgcttctttgctctttcgtctcactccatt
tagtcaaaaattccatttgtgatataaaaatgaatagatgaaaccttgaaatatgtaatg
aaaccggtctcgagatgtggttaaagcatatttgtaatcaaaaatatataattaatgaag
aaaaacaagtacgttttcggaccacatcggaccagtagaggcacacaaccatacgggtgt
tgtgcccacttttgacgacattaccatcagaccaaaggaatatatgaaagatacatagag
agagatatgttctcgaactaactatttcttttttaccctcttctgattataatattatt
ttcccgttttgctgttctcttctaccaaactttcctcttatatcaaactttcaaatcgaa
ttcctgaactttatatcacagtccttatttgttttctcttgcaacaaatgaattttttg
ttattgttgaatagttatatttttaaataagtagctttgacagaatgtatatttaatgta
gaatagcaaataaactagaaacaagaacacatataagggacatactggtcatttcgaaag
tcaaagccagaggagaagctactatttctccaactgtctgtaaacgaggaatttttacaa
ccgcaagtcaaaggatttaaaataagtagttatggttgtatctgtcgttcttctttctct
atgaactaaaggccgttggaagcaacacacacacacacacagtgaaacactacagtttac
agattcgttttctttagctcaatcgaattatattatttcttaatcagtcaatatttaaat
aatggaggatgatgataagtattaaataaataattattctaggattaatcagacatgcgc
agttatataataatcagtttgattttcttttccttttcgagcccctctctctctcactct
tttcttttccgagaacccaacaaaaaaaaaagctactattaatccttcccctcgtgaggaa
atcatttcttcttgtttctcgagatttattctctttctctctctctttctctgtgtgttt
cgtgtcttcagattagttcg
>transcript:AT1G71030.1 - Up_Stream_Len 2000
gccggaaatatagatttcgaatcacaaacgctagtaacgcaaggttcttccggttcttct
tctccaacggtctcgacttcattgtcgttggttccgattccgcttatctagcgaaaccag
tatcgaccaaatctgttctcctagctccgtcggagattgtcgacgtactcgttgactttt
caaagtcaacatcgaaaacggcaattcttgccaacaatgcaccttatccttacccgtcgg
gagatcccgtcacggaagagaacagcaaagtcatgaagtttataatcaactacaaatcag
aagttgacacgtcgattattccgaagaagctaattgaatacccctcctgctcatgtatcaa
cctccacacgtacacgttacattgctatgttcgagtatgtgtcaagcattgacgagccaa
cacatctatacatcaacggtctgccttacaacgctcccgtaaccgaaactccaaaaatcg
gcacaagcgaggtatttataaccaaaaatttcactattcactaaccgtatagttcaaatct
ttaatgcttacaataatgaaggtgtgggaagtgataaacttaaccgaggataaccatccg
ttgcacattcacttgggattgtttaaagtattggagcaaacggcattggtgaagagtgag
gagttcatagagtgcatgactaaaagaaacgatgccgtcaagtgtgagattagcaaatac
gcacgagggaacaagaccgcggttacggtacacgagcggggatggaaaaacgtattcaag
atgatgccgggacatgtaacgaagatactcgttagattttcttacattcactcaaacgaa
tcttactcctttgacgcaactcaagagccaggctatgtttaccactgtcacgtaagttgg
taaaatactaaaaagttgatgacgaatttagctttttcttttagaactaactgtatgata

ttggcattgcagatattggaccatgaagacaatatgatgatgaggccctttgcaatggtg
ctataattctaaaacgaaaacaatttacaattacgagtgcagaatacaaacgagttgata
tagattgagatttcagattacataaatatcttatacagaaagtgttaaaagataattcga
ttggattgatgttatatctctatcattgtttttcacttggcctcggagattagaagcagc
ttgttaatgggcttttaacagttacgggctttaacgggcccaataacaaaactcttttgc
ttcttatatgattttggagtagatggtaagtgagatagggaagtggtgagtgaaattaga
aaattcctcgtatacaaagtcctaaccaccagtcaaattcaattgaatgctcccatgttt
ctcccttatctgttcctgaacctacactctttaatttcatggtttcctattttctcttaa
tttgtttattttcatgtcatattcggaagcttcagatattttatcaaataatgcatccaa
ataattttgagccactagtcaaatatcttcttcgtcttcttcttgcttaatatatatttt
agtgaatctattattgataccacacacacatctttgatatttcacaaaaggtatctatcg
gagccgtacatatattttacgaaaatgtggataagagcgagatttggattggtattggta
aggcaaattaaatgcgacacacttccacattcatgattccacaaattctaatggatttga
acaaatatgggaccaatttttatttgtttcaactcctaaatgtgggaccaattaacaagg
ttgatgtattcttcttgttcttcctcgttcattaatagtttgattgagcatgcacgcttc
tctcaacccaccagtccaagtcaaacctcctcaagccatcccctaatcctatatatcagt
ttcttctcgcaaaccacctcacaatctatcaacagttttaaaaaaagacataactcaacc
aatctcactttgaaaaagac
>transcript:AT1G18960.1 + Up_Stream_Len 2000
gacatttatggggaagcagtttatgatgaagaatatctgaagaagcgtaaacagaaaaag
ctctctagcggttctgagggagatgaagaaaagggagatgaagaatacaaatgggatgaa
gacaatgctgagtatgaggaagaagaagaagaggaagaagaagaagattccctaagtgca
agcgaagaggatagtgatgaaccccggagggctaagaaaatgccacggagagaaactaag
ttaagatcaagatcaaatgattttcggccaggcctgagacgaagtaaaagagccacgcga
attgattaccaacaatatgagttctcagactcggacaaagaagcaacaggactggcaaaa
cgtaagcggtttgttgagccggacgaaccttctgatgaaacagggaatggggatttcaca
atgggaagtcaggactctgaagaaaatgcaaacgacccagaaacaaaatctggtgaagaa
gaagagccgagagatgtcaatgacaatgcagatacaacaaatggaaaagagaataaccaa
cttaacaaatcaaacggcacaacagaccaagaagaagttgagggtgtagtaggcaaaagg
cgttacctcgacttaaacgagctggccccggtatctggttttgatgatggcccaagtaca
gtattgaaggatgatgataagacagacaattcataagcaagaaagagagtttgccttcat
aaagctccctctggtcgatcaccattgtatgataagcaagattttcacgagttatttctg
gcaacatccccttgcagagaaaagttgaaacggtaagagacactccattggctttttctt
cattcaagcatatctgccttcttgttgttttaaagtaattcaggcaaattcggctgataa
gtgaagaggagaatgttatggaggccgaagaggtatgtgtagattaggttagtcattttt
tcttaccatgaagaaagaaacaatgaagagggtgaaccagatattcctaagagctgttta
atgttttatggaattattattagtgtacaagtaactttctctaactttttgtattttga
tcataagaacaagttcaaggtctcttagattttggttaaacgacgcagctttgagactgc
tgaatgtgattatggtaccatggagcaaatctttctaatggctgtgaaacaaactattgt
tgctttatcccaggtcatattgtgagcttcaaagctcatgaaactacataccttcaaatt
aataacttggattttagatatatataggctatatatatatgtacataaatggttattaat
ttgatcaaaactacaatttactaatgtctatgcatatgtatttaaattatttggtttgtg
attgcacaaacttgttgtgatcactaaattcattgtcatagggaacccaaatgggtttac
aaaaaaaagagaatccaagtattttcccttttacattggtgctaatttaaaaataacaaa

aaactgttttaattgttaccacttactactagaaaaacttgtacaatatgtcaatcactc
actaaaagatggcttataccctctttaatttcagtaaaagttgttatagattatattacg
aaacaatttttataacctaaaatgaattgtgctatagaacttggccaaatgtttattctt
tgataaattttggagattttggaaccaaagatgtggcaaaataaagaaaagggaaatgat
aattttatattgtataattaaagaatcatgcatatgtaatttaagaaagatgaaaatata
tattcatgcctttcttgccacacacgcttcttatacctccaaacccattttgcctaaccc
cttccttcttcacaacactccctttgtattattattattacatgcctcattctcaatctt
tctaaagaaaagcttttaatacatttctccaatactatagctataaacatcctttataat
tttgtttcttttaaaggaaa
>transcript:AT4G39250.1 - Up_Stream_Len 2000
ttagttctttaacttttgacctaccaataattagtgctattttatcgaaaatgatcgtca
ctcagttttgcgccgatccaatgtggacattgggtatttaatactagtatcttctgttta
aatttttgactgagaatatatagacaacaagatattcatcgaatattccccttccgttac
ttttctttttgtcggcatctttttatcgtgaaaggatatcatcgtcaacgtagcccaatg
agctcaaaccaaaaatgtaaatcctaatccataaaatgactattaaacaaacaataaaat
cacatagatgagactttggatatgctacaatatgtgtacttttgtctcttgtaaaatgtg
taggcttgttgagcaatgctctgtttactcactctccaagtgacaacaacagtctgcacc
gcttggaagaaaaaaacaaacttagcatttgttaacaaagttattatgaactactatagc
aaccccatagaaataagcatcttcattgagctacaacagctaagtctacaaaggaactta
agattacatcaggatttagttcctcaagttgcaaaaggagctaccactagttgaggaata
taatgcttaccctttcctttcttttccccacatattcttgtaaagctacagtggcaattt
acaccaatcatcaccagctaagcaacgtgtttcataaaatggccggtaccttcactatag
gaaatgcaacttgaagatggagatatggaatcaaatccaccaaatcttaccctatgtaac
ttttcatgtcacaaaagtaaagatgcagcgaatccatattgcataacaaccaattctgat
gtttctgacataaactattatctcacagctcaagagattacaagacgctgaaagtgaaat
gtatatttttaccattttaacacctaggtaagaaggaaacaatgatgagaatatatatc
gtgaaatgcctacttgtagaatcccgtagcaactagcaactatacctctagaatatattg
agccatcagtctcatgattaagattcttttgtcttgtttgaacttttctttcttgtaaag
tagtttatcccaaaaccatggcagtctcttgagttctcagaacatcaatatcttaaaacc
taaaacttgtaccacaattccacaaatggaaatatctcgcaaggccacaaattggtgcac
atccacaagatagactaaatctcgtaagcatgttaaagaattaatgcgcatgaatcatac
catgttagttattgaagtaagtagtgaccatttatttagtatgacatctaagaccaaaat
gcaaataataatatagatgaccaagaaaagcaacgttgccaaacttcaaagagacttctg
tatcttgatatgatgttcaagacagagtatccctgaggtccaatcaacaataaagtttga
tctccaactttcctgcaaggactttagttaaagagaagctatatggctcatggtgaaccc
ttgaaaagttccacctagttaatatcagaaacaatgatagtcaatgagatccaatgtatt
tgcaaatttttactgttgtagtcaatgtatatcagagataacgttaggccagagtttccc
aaagtagatgaagacttaaatctagaattgaatgagaaaacctgctctgacggtaacacg
tctctaactcaatcaagaagacatgaaagtaatcacagcagtaaaaaacacagaagaaat
atttctgccaagtatcatatactgatctccacaatacttgctgatattctctttttgctc
attagtcgattactatggagacaatgacttaaaattctcttcgtttttgcaagatatttg
ctacatctggcaccaccgaaactcaccttataaataggaagcattctgcaacatctatgc
acatatcatcctcttgactcgtaatctccccatacttaaataatctctttctaaccagtt
atattagtctataactcaac

>transcript:AT4G36570.1 + Up_Stream_Len 2000
aagttgaaaggagatgagtatctgccaaaagaatgttttatcacaaagcttagaatagag
accaaaagggtactacgagagctgatatttttaaactttttggagaagaaatacagtcca
tatcttcatctttttgttattttatatcttataataataagtacaataaaacatagcgag
tggagagggatcagagtagttaaacaattaatgtccaaaacaaaggagcagaagaagcgt
aggaaaggcgcagatgtcgtcgataaaattcatctacgagatcttcccaagatttggaat
ttgtccacttgtattgccactcaatgttaaataagagagaaatataaacaagaacacctg
aatatcctcgaaaaggtgctgtttttttcttttctttttttcgtttgtgtcatgcatta
ctaaaccaaagctaaatatgcatctcgtatatgaacaaactaaacacacacacaaaaaaa
aaaaaaaaaaaaacactttgttggagtgtctttgtaaaagtacttaatttatatcttacg
cttgtgtatatctttagaattactatcattaaaatatcttttaagggttatacacaaaaa
taattctgttaagttttaataaagttgcaaacaaataatatgacaagtattgacaatttt
tcgtacagaactgtttcaactcattctccggattttatgtatataaaattaatttttaat
taagcattagaaaacattttgaaatagaacaatttctagaattttatctccatgctttac
agttttatatccacgttaatttttttatgcatgcgccaaacggagtacatctttaaaaa
ggagtggtatgcaacaaaagtagctgctcaagtttactataaggtttaaatcagccaatc
ttcttagcccattttaagaaaagaatgacaaagcaaatattcctatacctataaaagata
aatattgactaatttgatgacgaattaatcaataatactataacctaacaaggttcggtt
cttatcatcttatggttgccaaagaatgtttttgtgatcacggtccagaggattacatgt
ttaggggctaatctttcattattaattaagccaaatcacttcttagtactactctgatat
ttgtcttctcattggaaggaatataatttaaagctaaagagcatcaagccatgtaaagag
tttgatgatattcactaataagggttgcatgcacgagtttctgttcttatcaacagatgc
tcaacacgagccaaatgtataaaagagatcactcagataattctattaccaaatttgcta
taagagccatgagacaagaatggcaagaatcgacgaagtgggattgtgttttatttttat
gacattaccaaatgacttcgactatatatggcatctacgaaaagacatatcatcactaac
ctatccatcataaacagatatttgatatttccaatcaaaatggatggagatcaaaagaag
catcaaacataatctaacaatgtcatgtgtttataataagcatccgatactacggtctag
tgatgactagatctatacaagagccttaaagaggtaagggaaggtccacaaacatgcatg
ggttactgacttagtcgtttactgtacggaaatatagtagcaattaatacaaaagaaaaa
cggggaagaaaacatcttatatttctctattgcaaggtcaaggaatctcaagtttcattg
gattgtttcacaattgtgtgtaccttctctaagactttatgtggttgctaaaagaaatag
tcactctcttaaagtatatattcttccaactggcttaaccccacaccatgctttttcctc
ctacttataaggagcttctccttctcttagctaaccaaatcaactcctttttttatcaagt
catagatacatcttccttcttatttttttctccctcccaagaagcagatcttgtactctc
gatatacatacttattttat
>transcript:AT1G19510.1 - Up_Stream_Len 2000
tcgaatttttgtaagaatttacaaaatttgtacaattttatttatttatcgataaattaa
tacctctctaaattaataaattttattggtccaaatattattaatttatagaggttttac
tgtatgtcaaagttaggcggcgtgagtgcatttttctaaataagtttgcgttctttccaa
attgtaactagtgtttctaagtcagaagtagataaaggtttcgtatagttgcggtaatca
tggcaattctttgatatagtaagcacaaaaatgatacatacaaatatagtaatatacaat
ccacacaaaataaaatggtctaactagtaactaatactaaaaactaaaaacaaattacct
agtacttgagtaagaatgagtgataacaaaggtgactacatcaaaaactcgtgcatccaa
tatccaaactcggatcatgtattatctatgaagactcaattagttgttctcaaaaatgtc

cttcaccaaaacatcataattcattcatgtcgggattaaatttgccatagtgaccgaaac
tatttctctcatagctaacaaaaacctatagagagtaggatatggtgaatggtgtggtca
tcactctcactctagtcaatatggagtaaaaaccacataatttagatgagtcgtgtaatc
ttctaacttatattggaagagattgtagtacgacgtgtcttaaaacttttcacaataaac
gttatgtggtgtttttgctgcaccatagaggaatggtgcaaaaagacattattatgctat
gagaccacactgaatctccaaattcttgtataattatcagaatcaattatacatatataa
tttctgttaagaaaaagaaataatgaaacatacactttactatatttttgacaaaaaaaa
catataatttgttataacttataattattaaaatgaattcattttcataaaacaagaaag
gattggataaaaatgtacaactatgaatgagaatcaatgcagagatagggatattggaat
tttatcccaaaagaagacgataatatctcttatggtattattctttttcagctaaattt
aagtggctcttatcaaaatggttgtcgtccaaatttacttttctccatcacacgggataa
gttattagaattaaaacaaagtgcccaagagtatcgatcccatctccttcaaattattat
tattattgtatattaatcgataataagttaataacaaataaaagttatgaaaaatctcta
tgaagtttagtacaggagatgacatatcgaatccattaattcgatattaaggaattttct
ttttcttttcatatcaaacgatgattatataaaaccattttaccagtttaacaaaaacta
tttcgtatgtgaataaactatcataattcatcgtcttagcacaactttgtacgtacttac
attatagctaaggtgaatctgattcattggagtataaaagacgggatcttgtttgtccac
tcttttttctatgaataccaatgtctttaacttttcttttgagtttttaatgattcctt
tacgttattatgttaaaaccaaagatgattctactacgtaccctgcaagtcataggctca
acaaataaccattaaacgcttaaaccctataatttaaagaggttactaattttaaacttc
ataagaacacatacgagcgttaattaacggttgacttctcgacattcttcatacaatacg
acactatgctctgaaaacccttatggaccttaaaaaatgtagacccataaaaagaactat
aattctttattttggtatacaaactaaaaaaatattggataaacactacaacttgcggaa
tctctcaactctcttttcctccatataaggagcctattttggcattcgtttgtattcgta
acttagtatcgatcccttttctttctcttttcttgtttccctatatatatctcatatatt
cccaaaagaatttaaataaa
>transcript:AT1G75250.1 - Up_Stream_Len 2000
gaaaagtttcattaattctggaaaaaaactgaatcatattcaagaagaatgagtgaaacc
aaatcatctcttagctattatgtgaaattcaattcatcaccatttcactcatgtttattc
cactaagcactcatcttatacgttcgatatagacctatcataataattagtgtttctgat
taaccaagaacttttgtatttcgttttcataacatttttaaagcttatgtatacaactat
acatacataattgttctttactgtgactcggatctaaaaaataaccaagaaaatcctttc
aaaaggccttagactctaatcaaccaaatatgaactaccaatgagaccatggacaacttt
ttggtcgttttcattagacatgaagtatttacaacagaaaatagattagatttggatata
ggaattttatcacaaaaggaaacgataatatcccttatgttcttattcttttttttcaag
ttttctcggttatgtccttattctctatgcatcaaacttttggtggctcttataaaaatg
cttgtcattacagatgggtctcttccattacatcacacttaaagtcattagatccaaggt
ccaggagaatatttatctatatcattttgaaatggtaaatgaaaatgattttctttaaca
aatatccacgaactttggttaaggagataaggatgataacgtatcgaatcctttatttgt
ccaatctctagcatttttgcattcgtttcattattagttgatgctgggaatagaacttaa
actatcattcaaacatcttttaatatgctttggatgtgaaaatatatattaggatatcta
caagggcctaaatgtacgcagtttttagcaactagttcacttttcctcccacataattag
ccagaaacgatgcccaaataatggttcatacatcatcatttagttacatgattttttttt
tatatgtacaatttaactacagaatacagaattggcatatctttctatttacttgtgact

tcatatcaaaacctttcgttccaataatgccatgttatatgcactatgtctactaatat
tttgttaagttaatcaatcaccacacaaattatcttaataatggcttacgttaagatctc
gaaaactgtgaacgaatatacaaaaaaataagtattgggactatattggagaattgttgt
aatcttttgtctatcataatcacattacgccaagttgttcaacttaccaactttaacctt
gcgtttttaaccggtttatcgatatgatcaatcggttccaacttttaatctgcttagacg
tgtattatccttctgttgattgtttagattcttcttctgaaaatttatcaatcatataga
tgcgtagacgaacatacgactatatatataactaactaaatagctgctcctaactaatac
taatgcgtcagttgtgcgttatgttttacatgaactgaaaacaccaaaaaaaaatctttga
aaagttttgaaacccgatattttaagttgaatagactttgtacttaaatcaactggtaat
gataaattggttcatttcttaattattactttacgttttatcacactttccatgtgagtt
tttaacaagatacacttaattttcagaaaaatacatcagaaattattactatggcgttac
gaatgactcttttgtagaccattaatatggttgttatcgtgagtaaaatatctttagtat
caacttaattattatagaccactaatatggctgttatcgtgagtaaaatatctttagttt
tcaacttaattaatgaaaagctagtgaattgaaaaagaaattaaaatagaaaactctcca
acttgcagaatctaggaactctctttttctcctccttataaggaaccctgccttgctatt
cattttatagagcaactttcaacactcagtactctctcttttcaatctctttcctttcca
tctcaagttataagaaatta
>transcript:AT2G18328.1 + Up_Stream_Len 2000
acaaagcaaaatattcagagaaaaatagagtccataactccatatatgtttgttttctta
atgtcatttactcaattatttttcatatataagtaataaccaacatatagctgtcaaaaa
aaaaaaacaacatggttggaaaatggtcacaaataaaacactgctaagtttgccaaaaaa
aaaaaaaaaaaaactgctacatgcaagtggagctaccacgcaaagttagaggcttaaaca
catttactaaaaagtaaaactttaggtacgaaagttccataacttctcaccttctctgta
gtcgaagaatctgaggattgataaatgttttaaacaaagaagcacaacaagcgtaagaaa
ggcgcagaagatctctcgaagataagatcccaagatttggagaactagtccgattctcta
accatgttcaaaggtgaaaattaaaaagaagacaagaacacctgaatattctttgataaa
tgtacttcttttcacatctctaggccagccagtaacttcgattcaagaactcaatcatcc
caagagatcaaagagatttacacaagatgaactagtttacaagaggctgcacaaaagaaa
agaaaaaaatggtttggttagtgaggaatcatcttgattaatttttcattttaataaatg
aacatatcattgtcaaagaatgttattccgtggcacccacgaatgaacatgttttagagg
gctaaaatctctgattatgccaaagtacatctttttttagtactgtgtgtcgtctgagatt
tgtcttattgtctaataattggaatcatgttctgacgtacacctactagctaaaggcacc
agacataaatagtgtttaatattcagcaaaatcatcgtccttttctatagatgctcaata
taaaccagtaaatgtatagattacccaagttgctcaacgatcttgaagccaataatggca
tgattcgctttttgtcacactccggtgattaaagacttcaacagagctaatcagatcgaa
aagacatctatcactgcaaagaagtacttaactatatacttttctcgatcacacaagatg
gaaaaattgcaagaaatattacacacaagcttaacaattcacctacaactacaacactac
aacagatactcaataaatttcagtaaatataagattacctgaagttgctcttttttttatt
tattgaataccatgaggttttaagccgaaacccgtaattctccaaagcccgaaatcacat
catgtaatattagtttcatctcaaacgtcactcgaaccggcgacctctaagcatgtaaat
tgcttaacgatctttggagccaagaatggcaaaatcacaatcccacaatggaaacaatat
aaaagttaactaagctaatccaacaatgcaaagaggaactctatagagattatattattt
tcaatcatacaagaaaaaaaaaattgcaaaaaatattaaacacaagcttaacaatcacatg
ctttatgaatagtatcggatacgataggggggttaattcgacactaatgatgatgataaga

tccaaaatgctgaaagaggtaagggagggtccaatagttattggtaaactttacggacac
tagcaagaaaaaggaagcctcctaatttccttatagcaatggtcaagagaatctcattcc
actaaaactattaaacctttattatgtcctttaagggactagactctaatgatcattatc
atatcaaagcttctatcaaaacttttgtcccttcactaaaattttatgtggtagctaaaa
gaatggtcattctaaaaatctctcatttcaaagtatatattcttccaactggcttataac
cccacacattgcttttctcctcctacatataaggagctccacactccctcaaactaaacc
aattcaagtcatctctatccgtcaacaatacaaaccaaccttctcaattcctctcttttc
atccacattttcactagcta
>transcript:AT2G42150.1 + Up_Stream_Len 2000
atgtttaactaaactaaattggatgacataatttgaaagaattatatatgcaaggtgtaa
atttaacgtatagacatgtcttgataattaagaaccacaaatgtcacagctagtggtata
tataaaactaaattttaaatacgtatatatgtgtgtgaaatgtcatgataggttaataac
catttaaacgaacttatgaatagtaacaagacgaacttatgaaatgaacttgtgaaatgt
catgagaggttaataaccatttaaacgaacttatgaatagtaacaagacatgaaaagata
agaattaatgtacactttatcaccaaaaaaaggaagataagattgtacgaaatcggacgt
aataatgtaaggacatggaaatggcatgaccctcgggtgcacgaggcagttccacttcct
gacgactctccattttacttggtttacttttatcttatctcttttcctttttaccttgcg
aatcaaattgaccttttaacaaattcaattcctaagtgtttttgtacaatatttgtgaat
tataatgtgtagtgtatgtctatgcttgtttgattttctaacgcttgttggatataagaa
acaattatcttgaaatctctacagtacttatcacatccaaacacactatataatatattg
gacaagcgatacactgtcttatacaaaacaaaccaatcacaaacaaatagaaaacaatac
atctgtttcgagtcaagtaacgtcctatatattttatatagaaagtataaatatacacac
actagttgaaaaacttgaacaagaacatgttagagggcatacatatatcattcttactaa
caagagatgttttgtataattaagccaagactttctaatattatatttgtgtggtataga
tatcataccaacaattgaactagaataagcaatatatttaccatactatatcttgtttac
ctaagatttaaaacggataatagccatcggatgtacatgtacacgtgaagagttgggata
tgaaagaaatccaaaggctgagaaaaagaaagagaggtgaaactccaatgaaagaattgg
gtttgtatacggaaacacgagtcttgtgctctcttttggatccggaccacacgagcggct
aaagaagcaatgttgtcaaaaacgttagagccgtatgggttatgggccccaccatctctt
tacttgacgtggttcccagttccaaacttgtgttgtgtatatatcttgtttggctggcaa
ttaatttttgaaaattttaattcgatttctgaggacaaaagtggttgctgattaatcaac
taatccaaatttgcacaatgctaattcaacactcgtgctgcattaggtgaaaacttattc
acactgggaaacctgcctttttatatagtatagattgtttctcatgattattgttgatta
attaaaacatttttaaaaattagtgttgcaaaagaaaactaatcttatatgttgtttta
ttatatgaatgtagatgcggatatatgattgagtcccacatgctaacacgtattgttccc
tgctggggaagaaggaaatcaaaatcatgggagaattataagcatattcactcgcctacc
taccttgattactcatcattcataatttcatagaatcattttccctaaagaattaaatac
acccttgatttataaagtttaaacgcttaaaaagtccaataacgagagggcctttgaatc
ggattcgcaattatatttgtaacaagtaaataattgggccgactaaaggaatgaatatac
ttccaaaacaacattctcagtcggtgcatatggtacggacctaacgaatccaaacctaac
aataaaaagaaaacgttacaaaaaaatatgtaacagagaaaataacaaaaatataacgaa
gggaaagactaaagaagagacacattcattaacaattcatacacctttgttcaaatcaac
gaataagcttctttatttta
>transcript:AT3G57980.1 - Up_Stream_Len 2000

aatctggcccttctctcaaagtcgcttaccgtccaaacgattcgtggaatcctttctctc
taatcgtcaaaaccggatcgggatcgttcggttctccgatttcaagctctatgctcatga
gcgctgaattcaatctcctaggtcaaggaaaccctagctttatgctccacttcaaacctc
aattcggcgatttctctatcaaaaagtctcattcttcgtctggatttgagaggaatttga
tcaaatcgatgaatggatctgtttccgaagatgattcttcgattgaggtggtggatactc
cagcggtcaatggatgcggcggaggatttagaaaagtcacggttctgccgtcaacttctg
ctggagatatcgctggattgttatctggcgttgaggttgcggcgaggacgtctctgcctg
tgagaggacgcgccgttttgaatttccggtggggcgttagggttcctacggagatcagac
gcgatttcgatccaacggctgctatttcgttgaggagattcccttttttggtgatgaata
aaataggaatcgaacacgtggacggcgcagatgctaaagtgaccaaatccactggtgatc
cgggtaaagtttcgggtccggctcagttcacaacaagtggtgacgtggcggaggtgatag
aggagcttcgaacggagaacaagcagttaaagagagccgttgaagatctccgggaagtaa
tatcaaacgtccgtccatactcgccggcgacaattgattacggatcacactcgaagtatc
gcgaatccgagaggaacaacaacaacaacaacaacaacaacaacaacaacaacgggagat
cgagagctgatcggtggagcagcgagagaacgacaacgtctgattacggtgggaagaaaa
gcaaggaagagggcaatgtagctgaggagctgaagaaagctttgaaaggagctgcttgaa
gcttctatggcggctattggtaatcgcaggctttactcgaattacaattacatttgtcga
ccttaatcagctattgcttttgtatatatatgcgttgaattatctggattagatttgcac
aacttgttttgaatcttttgatggaatcttcagagttttgtatgagattatgcttttcaa
ttctctttattgaacttatatgcaggttgattgttgaattgatttgaatcagtattctat
tatagtatcaatcaatctgaagcttgatggtgatacactgagctctattaccttgaacgt
attttgtatatcttctttttaaggcattggcaagtgtagttgaattaaaagaacttatct
atgcattacaattcgagaagagtttgggaagagatttgccggtctctctaagtagcagca
aaactatatttgtcgatggcaaaagtaatctagacgatttaggacatctcagtatcatcc
tcggaagacatagtcatatagatacgaaagatccggatgctcatccatcaaatggtgaag
gtcaatgttatggtgaacaattagacacaattctttgtacattgcagaaaatctgaaaga
atgtattctctacagtactctttttttatatatttagtttggattaaacattagctgcttc
cgtacaatccaattttaaatgttatgagaaacgaaattggaccctcttggtgcttttggt
tccaaaccggtaatgccgtaatggattcggtgtaaaggttgggcctactaaagtgtgata
cgacaaaaacgagcaaagtttttcaacattctctcaattggtgcgcctttgcacgaacat
gttatcaacgaaacattacataaataaaaataaattctttgtcagacagattaaaaaaaa
aaaaaaaaaatcagtctcaaggtttttttagtggaggcctagagcaaagatttgcagaaga
cgattatttctatttcagttctacaaattggagaagtgacaataagcaaaatccactttg
ttccttttttacatttactta
>transcript:AT2G44430.1 + Up_Stream_Len 2000
gaatgctgatggtgttggtgactatgccaaaccagagggttattcatttccgttgtgttg
caaattagtagaaactttgcagaagagaagagaaaactttcacattcaaaccctctttat
tcttgggcacaataccaagttggattcacaagcaaacacgtgccccatttttaatggatg
cctggttagtagtctaatatgtctgcttcttctctactatgctaattagaagatgtacta
agtcattctttacaaatagagaaatggaaattgaccctcattcatctaaacttgataggt
gacatgtactaagtacacttttgtaattggttttcattgtcaaaatactgtctttcttcc
ttagggactaactagacaatagaattgcttgaaatttgttcatatgaatcagtaatcagc
gtaaccatgtttctttataaccttttttatcaggtgaataccagcaccggtgcaatcttgc
cagccagtttcaacagaacatctagatgtccggatgagatcgttagaagaattcgagttt

catcatcgtttgaagattctagctggaagggaaagttacttgacacatatgacacaaaaa
ctgatagattcatcatcgcaccatgccgctggtaaaaacaaaacgctacttctgtcacat
tacatataattgcatctaccattatatattgtctttctcaccagatttaatgtaattact
ctatccactactcctcattacagtttcataaatcgtgtttatgtttgtaatataggacaa
tgcgcttgattgaatatgtttgggaacttaatcagcttactgatgaagaaatcctcacta
actgttccacctcaccttctgctgaaggtccagacttcgtaaacagtttaagaaggttag
tcattttggaagcttccactggatcattatctagaacacaaacaggacataggaaaattt
gcattctctttctttttgtgtgttttaggaattggggatatttactgaaatatccagaat
ggagcaaaacattcccaaggaggcaaccacgtgtgtttgaaagaactgtcgatggacact
ggaaaaaatgctgactttctcatctaatcatcatgacattatccagaaatctggctaaaa
acaacggttagtgtatctaacattttgagatttatgtaaatggtcaagaacacatgtgat
tgattcttggacacagaaaatgtttgatttatctttttgactttttgtatcaaacgttgc
aagtttggttcctatgaaaatatgggccggatcaggcccggtagacctatatgttgccca
tggtagacattccaatctcaaatccaaaccagagacattccaatcgaaacaataatacac
ttaggccttagcatgatttcgtaaacgtagtccgacctaactttcaccaacaaaacgacg
gatataaggcattctgtttctagaaagtagaataaaccaaagagggccattcttgtaata
ccttatttccgaaagatgttgaactaagcctaaccgggctggatataataaggcatcgtt
atgggttttaaatttacaatttaactaacgaaaaggaaaaagggaataggtcaaaggata
aaaagcagcggttaaaactagggttacttaatccacttttcggttttaattacttattta
tttccccttgaattggtgtagtcgtatgctagtattactattagatctcattacttatag
ggaacaaatataaactcgatagtttgatttgatttgaatttatttagattagattatacc
gtaatgaaaaaacacagtagtatttaaataagaaaaaaaagcacaaaaataacaagcgag
cgagttagacaagaagaaagcaacaatacaaatttaaatacaaaataaattatataaaaa
gagaataaaaatccctaaaactgcgagcgtttttgtttgtgagagagaagaagagagaga
cgagtctgtatggcaaaagg
>transcript:AT3G60110.1 + Up_Stream_Len 2000
aatatagatactacacggtgctttttggcgtctcaagaagtcttggaatctgctcgcagg
tacatacatacattcatgccatcgacaattcgattttagtacgtatcgccatatccttca
caataaagtcagactaatcttttattccttttctgtttcagctgatatgggacagggcac
ttggactgccacttgagagaccaaagagtgtaaacatggactggctcgataacttcacgc
gcttgaaccgctaagttaatccctgactgatgttggtcaaggttctctcttttatcactt
cagccttctcttccatttgattgggtagcttttagagataaaaaaaaaacgatttttcgt
cgaaattttgttctacagagttttcattttggttcttctgcaaaaacaataaagtcaaac
aagtaaagaaacagtctttctcagattgagacaccatattattgttcctcaactttgata
gacaaatgatgaccattgatatgtattgattagtctttggtgaattatcgcataataaac
aatactcatgcaaccagtcccgctcaaacagagtgtttgaagtgaacagaagcagagtca
agaaattcaaagcctcgggatcaaacagaagcttcctcctatcttaagaagctccctacg
aagctttatgatgattctacaaaacacatttgctatcttcagtgaacagtaagatgcaac
gtgttctcttctaaaccttcagatatttggctaagtataagactcttcgtaatcacaaaa
agccagaaatgcgaaattgattttgagtcttcttcctcacaagttttctctaactttcag
aggagtcgctggatcctgttaccgagaattattagaaaccgagagcagaggtttctagct
aggacaggaaatatattattagaacatatcctagtcttccttgtcatggccagtccagtg
gtgcaagagaaacatacaattccaactcaagtatgtgatggacttgacttgcttaagccg
tacggcatcctcggaaaaccaaaaccatgtcggaaaaaggaacttgacttccttgaggct

tacggtttcctgggctcgaacccaactacccaagtcaagtgacagagagtcttgacatag
attaggcacctccttactaaagccagacaactatacaagattattggttcaaatattagt
caatcctttcgcgttttctggcttgacccgtctcgtcatgcaagggttggtcacgagttt
cttattcttctaaagtttaccaaaattaatccccttttctttatctttgcgtgtgagtgtg
aattaaagtattgatttatttaatgatgggtttaacttaaaaagtttaaaattgtgtcat
caaaacccatcaagacgtgtcttgtaactctgtatcacacaaacattcgtgtgtactaat
aaatgcaaatattttagtcacaccttttcgtaattatttatttatcatttaaataagtga
caaatctgggtccaatctccattagaggcccatttacaacgaattaaagcccatgtcttt
ttgaagtggataaattccatgcccaagttactcacattaaccaacatcgatataaaagga
acattctatttctagtaacccatgagggtcattcctgtaaatagatctgccttccttcct
cctctccgtagacggttgttgcacacaaagcgaggttggtttgacccgaacctacccgac
ccgttactatatatataaacacaaaatgggtaaagcaaaggtgaaaatcttatacgtagg
tctatatcatttccatatgaagtggagatatatatatatagctaacgagaccctaaatat
ttttttctttcttataacatatagtttcatttcatatatttaaaggcttcataatacgaa
acaaggtgataagaaaaaaaaaaaagaaaaaaaaaaaagcaaattaaaaattaaatccctaa
aaacaaacgagcgtattgtt
>transcript:AT1G09710.1 + Up_Stream_Len 2000
tgccccctaacgtattctcacagctcccctttaaaccttcaactctgaaactaaccatga
gcttccctttaaaccttcaacgctcaaactaagcataaggactactgctgagatggcaaa
gctacaattcgctgctaaacccctaggctaggttcaatagttttgcagtgaacttaacaa
taatcacgagcgaaataagaaagatgaggtgcttacaacaggttgtgaaacacattggct
tagctcactggattttgctaattcccggagagcaacctcggcagctgattgctctgcagc
cttacgattgaagaatccaggcaaagaattatatctgacaccatccagtatcacagtcga
ttgaaataaagatttgtgtgaagggccttctttaacgatctcataaacaggcgttgggag
cttgtacttctgagcatactcctgcaaccgacttttgaaaacatagcaattggaaacacc
tgagcaggaaaaaaacttcaaaatgaggcaaaagataaaacagaaacagtgatgatgcta
tccactacagcataaattgcaaataaactcaaaacgacaacagggaacagcagtgaacga
gttctgaataaagctgacgactttcacaaagaaactatacttatccaaaaggggagagac
agttttaaaccaagcttctatgttccctaacaaattgagtcaataaatcatcatctcaaa
agagtaaacttgccaattgccagagagatctacgcttaaacccacaagggacagatacac
ttgtaaattctaactaatcaaaacactgcaatccaatttccaagaagaaagaagtgagta
aagaacagaagaaaggggaaaaagcatgatctggtttcaaaattaaaacccggaaaaaac
aacccatgaatgttgaattagagataatgcagacatgatggtgaagaagaagaagaagaa
gaagaagtaattcaccagaggaaacatcagtggaggtcattttcacgagcagaatgtagc
tgaagagacgaataataggttagtagggcacggcgaagagcaagaaagaaacgtgctgct
gctgcgaagaagcagagagagagagagatgattcttcttcaccaaaaaccctctcccctcga
cttcctcctctttactgatttttatttttgttttttttcacaagggcaaacgaaagaag
gtggttcttaattgttatgttacaaatgttaatattgaatttaatttcaaacctttataa
attatattatattccataaatttaccttttggtcaaattttccataaatataatttact
tgtgaatattatatgaaagcaaaatgtttttatctttgaaatatttaatttaattaaaaa
actaattacatatagcctaatttcgtatttcatatgtagttaactaatgtatatatccac
aattgattaaccttttttatggctaactttattcactagtaatccctttgttatattcaca
cagtttttgtattcacaaataattaatttgttctccaagtaaacatcaataatgataata
accgtttaataaaaaataaaaacaatttgagcccatatttgagcccacttgagcctatgt

cccttaacagaaaagaaaaatatttgagcccatatttgagcccacttgatcctatatccc
tttaccagaaaagaaaaatatttgagcccatacttttcgcttctgataagcgtagaataa
taatagtcgaatttaccaccttccttttcgttcctttttttcttctctcttccgaaaca
cctctgccacgcgagtactctccacgctcttcgacagtcgtgaccatcttcttctccgat
cgtcttcaccggttttcttgggcattccctcgtgcgtaagtctctgtcgtttcgtttcgt
ctataactccctcgggttttggtgaaatttgattcctttgtcctgttctctgcagaattg
ttgcaataaatacgacgaag
>transcript:AT1G58220.1 - Up_Stream_Len 2000
tgttaaggaattctttaaaaaaacaaacctgaggctagattgaatcttttgagatattca
ttataagatgggaatgattttagcatcttgtctttgttttcgacagcaaagaaacctcca
gtggaataacttccaacaggttcataagatggaacatcgatcgagttcccatctaacaaa
aacgataaattatcatagaagattgttatacattaaaataattgacgattgagtattctc
attagagacaacaaaaactagcataacaaaatacagttaaaatagttcacacataaaaat
tgaatatattcatatactctacgaaattttatgtaattaccacataattatatatttata
tgcctgcatgagaagaaactttggctcacccatattctcgaagaagcaataaacaactag
agccaatattgcaaccaaacctcctttgcttgatctcgctttcattttgatactaagctc
tactactaatttctttgttctgtttttttttattggtgttttctttaatacttttgagt
tgttttgaagtggaaggatatccgaaacaaagactacatgagttatttatataagatcta
aaatggattcaatttttccaaagtttctccaagtcaatatgtcgttgtcaaataggataa
atggatgattaaccgtactttacacgccttgacttgattgacttgaaaagttgaacttat
aatatggtttgtgttgactaggtgttcgttgaagaggtcatgtttgccaatataaaagat
atatctttttcatataatgcatatatgtctagaagattagtgtctactgtctactaattt
gaataccaaaacaaaattatataaaaaatgttaggaattctacgtcggtccaagtatgac
atgagttttattgtcaacaaatttgatagcgacattagttatcagaaggccaaaaaccac
aacagaagcatgtagtagggtttttccttggggattatgccccctacaaaagtccctcc
taaagttttacccttcttcttgcacacagagacacaatctttagaaacacaaaacccggt
ttttttgttaggtggaaacgggatacatcatcgtctgcttttcttttgttcgacattacc
caccacacctgttatcattatatttaaaatataaaatctcaatttcactattgaaagtaa
gtttgtaaatcttgtaaactacacaagctctataagtttgaaatatataatgtagtttta
gattgtagtaacgaaacagacaaactctttataatcagctgtctactatgtattaaaagg
gtaaatgttatgaataaaatctttaatatatgcttagttaaatgatatgctattaaaaat
cattaaagatgttggattaataaagaatacagtaggtatttaaatttattgaaattactc
ttaattttttttaagatatatatatatcatacagagaattttttttgccctcttccattt
ctaaaataaatattatatataaaaaataaatttgatttaagagaaaaaatattaagaact
tgaccaactataacgtaaaatcatatgtcgaaaaacgaaaagaaaaaaagccgtaaaagt
aaaacttaaatcggaggggcaaatcggtcatttggaaaatcgtagaagattctaatatct
agtcaagcggcgagcgtgactgcaaccgttcctcctcctcctccgtcgcttttttttttc
ctcttcaaaagaaaataaaaaaccaaagccgcacgcgtgctcttcacgcgctttcacact
ctcctcctctcgccgatcatcgctgcgattcccttctccatctccggtaacaattggatg
cgccgtctccaagccctagaacaatttcccagtatctctctctcattctcaaaaatccta
attagggtttgtcactgaaatcaacaccagatttggctcaatcgtaacatgtgattgctg
ttacaaaccacgaacaaaaa
>transcript:AT2G13960.1 + Up_Stream_Len 2000
acatgtcgtaagaagttccacaaagaatgtgtggagtctccacttgaaatcaaacaccct

tcctatccctttcaatctctccaactttatagttctccctttgctcttgtgaattgcata
tgttgtgatataagtttccaagacattatttatcattcttctaatcacaaactgagtatg
catcctgtttgtgcgatgaaaccagtacccatttttatagaccacacaaaaagacatccc
caccctctcactttttcccctagggaagctttcttaccttgcaatgtttgcggcctgatt
aaagaatctattctcacctatgtttgtgttccatgtgactttgtagtccatcaagattgt
ctatatttcacgcatgtcataaaaatatctcgtcaccaccaccgtatcatttttacctct
tctcttccatatggaaaatggtcttgcggagtctgtcgtagggaggttgacaatcattat
ggtgcttactcttgcatcaagtgtggtgattattttgttcatacaagatgtgcattgcga
aaagatgtgtggaatggagaagatctcgagggagtacctgaagaacctgagatagatgtt
gagccatacgagacgatagctgatggaattatactccatttttctcatgaccatcatcta
aaactcgagatcaatagagtttacgatgaaaacaagttttgtcaagcgtgcatccttcca
atctatgagggtacgtactattcatgtttggataaatgtgaattcatcctacatgaagca
tgcgttaatgcccctagaaagaaacaccatccgttgcatactcacccgcttacgcttaag
gtcgtcagtattggatacgatcattacagaagaagaggaaattacttcgactgtggtgct
tgtaagcgtgcaagttgtggttttgtctatgatagtcaggatttctcacttgatttgcgg
tgtgcctctatttccgaaccatttcaatatcaaggccacaagcatcccttatttctagcc
ttagagccagatgaagaaaaagcggcgatatgtcaaatttgccaagaagatggtgatgga
aataactatattagaaaactaaattgcatggaatgcaagtatattatttgtttcaggtgt
gctacattgccatacaaggcaaagtacaagcatgacaaacatttcctcacatttcgggaa
ggggaagagggaagtgatcaattagactggtgtgaggtatgcgaaaagaaattaatatac
tcaagaaaaggtgggttctatgcgtgtgatgactgttgcaccactctccatgttgattgt
ttgcttggggagatgagtgtatgttgaaacctggtcatactgtaatgacttataggtcaa
acattcatattcttcccaacaataccatgactcgacctttctgccatcgtcataaagaag
atcgttgtccacacaaagtaattttcaaatgacatgaaatgacattttgctcttacagtt
gttcgatggattaaatgtcatcatcgccggtgcttcgtcgtttacttctagatctctact
tttctagttccccatttgcttcatcttcttgttgtctggttgtttacgttttatcaaacc
acgaattaaactcgttgttttcgtttctattcgaaagaatattataattagcccagtcac
ataaataaattttgtaaaaaacacatgtttctcgattcatttaaccggtttattccaaac
cgactcgaaagaactataagaaaaacatttgagtcggttttgaataaaccggccggtgcc
aggttaacggcggttagcagttatgtcggcggaagaagtattcgttacaatatttgtttt
aatcccaattagtctccttctcctttctctctgctctctcgttcaacgggcaaagttttt
ttcgaataaagttttggtttttcttttaacgttcccttcttcttcctcctcctcgcgaat
tcgcttatctgtcgatcacg
>transcript:AT2G36960.1 + Up_Stream_Len 2000
ttaaactgtcgccgtagaaataacaattgggtcaagaaaagttttctaatccttatcaaa
atagaaaagttttctaatataatagaaaaaacacccgtaagtgggaaaacatcttctaca
aacttaataagaaaaggatatacatatctccaatccacatgcgaatagttttgggtcaaa
tctctacgcttcgcgtaacaaattgaatttgtcaaacaatttgagcatgacatgatgaaa
aaaggccagagtcatgttaattcgtcgtatcgtaactccgacttctgactttctccattc
tttttaacgaaacgataagtcaacttttaaccgaaaaggactacaagtcaatttattcat
taatgctctacgtgcataggttatacttattggttaccctatagaagttgaaccgtgaga
tcaaaaatcatgttagataaaccggctccgttctttgtgacgttgacatatatttttctt
aggccagagactatagctagataaataactccattcttggctgctcgtaggatacgaacc
caaaacttggtcatattttatctcaaaaataagatcccaaaaaatatcgactattagagc

tatcgataccatacgtgtttcaaagatgtaaatttgaacccctctaccttgaaagcgtgt
ataaggcttattttctgaaaaaaagtgttagctcatctaacatatacaaagattaaagat
tggatcgttgacagaagaaaagatttacaaaaatttggatcttgtgaattagttactatt
gggctaaataaaagcccatgtatgtatttattggaccagacctaatttttgtaccagggc
ctgaggactatatccaacacaaaccacaatttcaaatattattttggcgttttaacttct
aaacgctttatctttatcatgcgacttgccagatgccaacccaagccacgtgcattacga
ttcaactaatttttgttgtcttttttccccttttaacgaggcaaaatagtagaaata
tttttctattttcatgaaattgtagaagaaaatagtaaaatttccgaattttcctaatct
atagtttcactttagataaaataatacttagcgtgacgaaggcaactggcttattcaact
ttatgtgtttaataaaagataaaataaataataatttcgtatatatttctctcctttgtt
cataaaataattaaaatatttttgattgattctttcgaaaagtaaaaaaactcatttaat
tacaagaatataatatatgttaacccaccatgacaaacatatattttgacatcttagata
acaagtaactaaattaattacattcctttttattttattttctttttaattttatgtgaa
tgtaaaaaacaagaaaaaaaataaaatttatttttctctctcgcttggttcaaccgtggt
tgttcatcggaaaaatatcttcggcttcaatttcgttcgatttaccctaaattctcgact
agtgttcgtcaatcccacttccaattccacatttcaaaccttttcaggtaatatttctca
tatttttcgtgtttttttcggggtcaaacttattttttaaaaatttcaattcaaattt
cgagtttgtatttagtaaccctagttcttaatttttcaatttttcgtcttcccttatgag
atttagcttaattagggtattgaattaagtagaattgggtttttgtttttgttgtgatct
ggtgaaattaattttagggttttattgaaggtgaaatttgtttgttcttcgttatatctt
cgaaattagggtttttcaaatttgtgtcgtggatgtaaaaattatgaattgcctgtaaaa
tttttctgtcggttgctaaatttcttgtgattttagttgatgatcgctatgtagagtttc
gcctttttttgggattttgaatgttcatggtttaggattcattgctctgatttgtagga
agttgaaatcaaaagcatga
>transcript:AT3G21430.2 - Up_Stream_Len 2000
tttttatgtgatcatctctctttttggttattgagtagagtctagcgacttaacgttcaa
gaaaagaatatctatagctcttgatgcagccaaaggtaaatcaaaatggatgatccaaaa
ctcacattgattgttctaaaaactaatattaaagtctctctgtttttcaattcagggtta
ctacacttgcacaacttagatcctccagtacaacacaagagatttacggcgagtaaagtt
ttgctcgatgccaacctcaatgccaaggtatcagatggggcaatgttgggactgcttcaa
gcaagtgatattcatctcgctaatccaaggtattaattaattcacttatgatcatataag
ttacatcattccttcaaagtcctaacaaatatgttcttatgaatattttctatagaggtg
gttcagaggagacaattgatgtgtatagcttcgggttgttccttctagagcttatcacct
gtcaaaaccctggactgttacaatcagactatgaagttttacaatgggtacgtaatagaa
aaaaaaaccctctctttctccatttacaactttttttgtttgttaattgtatgtactta
aaaatatattgtttggaaatttatttgtgatttgtagataagtccgagactttttacaaa
cacattttgggataagaatatacctgcagaaacattcacgagaccatcgttccaagctta
tctgataattacgtcggaatgcttggaatatcccccgataaatcgcccaaagatggatgt
ggtcgtgaccgagcttgagacgatttaccttaatgtcatcagtgagaaccatgaggtttc
tctaggaagtgagctttttaacataaccattgagtaagcgaaagacatggtgttgttgct
tcagtctatttcgaaaacccctataaaggactataataaaattcaacttgactattacaa
accagaattaaaccgcattggattcgaactgtgtggtttaaatttggtttaaccaatcca
aatccaatttataaaggcgaaccgaaccgaattggatttaatttgtaggtttgttttaaa
aaaatgattgaatcttcgcaaattatttatatagagagactctctgcaacaaaagttgta

taacgaaaaattacattttatcggattcttctaatatttatttttctttttattttaaag
ttttcgcggttatatccccaaaaaattgtttcaatctcttgtttttgctgcttcttcatct
tcctcaatctctctttcgatcgctcaaaaaacccgaagaagctctgatctgttcatattc
cgatctcttaccggatttctctaatttctctctgtaatttcttcttcaagtcccggcgat
ggcttccgagtgattttggatttgtgtaggtaagctctttcaatcttctctttttttttt
tttttttaattctgatttgttctcatcgaattttcaaataatttgagctttttttaatcgt
tcgtcctgcaattttcgtttttattcggtaggaaagtttttggattaggtgcgaagtt
tgtgagtttaaaatttggttctggggaatctgcgcactgattattgagctttggattttg
aaattcgaagctcaaaatcgatgggtgtcaggtcttgtgatttgaattcgcataaatttc
actagctggagttgcttaaggagattgattttctaatttggacaagttgtttcttaaact
tctctgggtttcatattctattgagcgaaagtagggttgatactgataatgagcacactt
ttttggtatgaggtgggttttgcatgtagactttagtttcattagtactggtagtcagtt
tctttggagatgattaactgataaatagatgtatctatcaaggattgagcttatgattta
ttgtgtttgttgtatcttggtaaaatctaaacttgggttcaattttgggaatgtttcagg
taacagaagagctttaactt
>transcript:AT4G39160.1 + Up_Stream_Len 2000
tctaatctcaggctcatacgattctaattcagcatcgattgaggctgctgaagcaagggc
aagttgcccaacgtaatcctcaaagagctcgctaccaaagagcataccaaacacagctgc
aggatcaaccattgcatccctgcaagaaaatggaagtgaaattaacatactatagcaatt
ataccaatcacattctcaaatgacatataaacatcacagaagttatctctaggcttttta
tgtggggaaaaccgaaaatcttcaaatgtagaatgaatcatgaggaatgaaagtgtgaaa
acttactgttgaacccccttcttttccatatttgtcataagctgtacgcttctccggatcg
cctagaacttgataagcttcacctagtatcttcattcacacaacaccaatttataggtta
gagtgatccagaatatcatccaatcaaacactccttgaaaacccttgtgaaagtcgttaa
gcaaactaataaacatagcaatagagatgattctatattttaaggtgaaaggaaaaaatc
acctgaaagtttttagcagcttgaggatcaccagggttcttgtctggatgaacttgcctt
gcctgcagaaaaaaatgagagccctaaaatgatcaaccaaaattgatcttacaatcaaaac
ctaaaaagctaccaaatttggaaacacaacaagtttcttatcaatccattatctgattct
tagcatcataatcaaatctacgaatttaaaagcttatgcttggagtctgacaatctacct
gaaactcatagagatggaaatctgacgataaaacataaagacttacttgaacataataag
ctttcttaatctcagcaccagaggcgtcaatcttgacgcctaatatgtcgtagtactcac
tctccttcaccatagctcaactctacaaatagacgcaaaaaaaaatgttagacccctcgag
ctccctaataaaaaccattaatacaggaataaaaaaaaacaaacccttgaggaaatgcgaaa
caaacgaaatcagaggagacagaagggaaatggtaaaagttgattacagatcttaggaaa
ttttgaggaattaggaattgtaaatcaaattagaacaactttgcatatgaaaaaaaaaac
aactcaaaatctaaaattcataatctggaagctatagattcatacagagacttactgctg
ttcgcgaagcacaaaaactaaggatttcagatttggtgagtggtggagagaataaaatta
ttaatttaaacttgttgctttccattggaagagagattactgagtcagccacgttttact
acgggtggatttgggcttttaaatccaaaaagtcatttataaattgggctctctcatata
aagcataatttcattaagcccgttaggcccacgatggatgtaaagcatttaagaggttgt
gatgtggggtgtgagagaatcgaaccctagtgcaaggtcaaaaccaaactcgtagacaaa
ataatagaccactagcctgcacgatctgcttcattaactttgtaaatttattttgattta
tcaatgcgataatagacgcagatttttttccggccaatcttctcctccgagtagctaaacc
ctagcgacggcggcaacaatggtctagattcaacgtatcgtttcgtggtttatcgggtat

ttctagtttccaattctcctcatcaaagtttcagtttttaatcaccgttgatttttctat
tcaatggtacttgaattttctctggaatgatattcaattgttctgttttgattgcctgat
tgattacagtattctcctttttttttcttttccttttctgtgaatttatgagtttatgg
atatactaatatacatatatatcttgagatctgccattggtcttgtttttggcctagtat
aatggtttgacaaaagctccttctatcttgtattgaaggtgacactttggtgagagggag
agtatttgtgaaagaaatca
>transcript:AT5G41020.1 - Up_Stream_Len 2000
attgagatttcagcaaaaccatatttcatcttctcactttatgtatgcacaatctaaagc
aaacgccaaaaaaggtttgagctttaggaaccattgttctcttgctgatcgaagtaaaat
aagctatgaataagctatcaagtttgagatttcactagcaatcacattctcgctaaatat
atctaattaaaagccatttaaaggtttgagctttatgaaccattttcctctgcactctgc
tgctgatcttcatgtgaatgatgatgaagaaccctgaactgttctttacccatttgttga
aaaacctgtggattcaaaaccccaacttgagacagccttaattcaagtccaggcgtttga
ttatctccaaaaccaatctctggaacaccattgaaatcaaacccaattccgtaattcaga
tcaaaccctccaatggtctgtcctgtttcatttctccacaacccatttgcctccatttga
gtccttgaagcttcttcaacgctatgacaaatcatcaaatcagccgtgagaggaggttga
gaaacaacagactcagtgggtttgataccatttacagtagcagagagtatcgatggctca
gcatgttgaagcaaccattcaagagtctcaccatctgatttgtgacctaattctttagtc
aattgataaatccttgctgcacagagtggaggtaacctaactctacgacctcttccttca
actttaaggtgacgatctttgtttggtttcttcttctccttctcataaagaatctgaatt
tcttggtcttcctcttcattctccttcttcttcctcttttcgccgaccacaatgtcgaaa
cctttaacctctgtttgtttcctcttcttgtcgttgtcctgattctgtcgtgatgggttt
aagaaacttggtagattttggttcttcttgggctccatgaccatgtcaaggtgtggggac
gtacttctctctctctctcagattcaaggtggggcgtgtgttgtgacctcttgacctaag
taaaaaaaaagggtaacaactttttattattttcacgcattattttattttttttacata
attagaagttctataatcacgaaatattcttacttaccgaatgaattccaatccaaagaa
aaaaaaaaaaaaaaaaaaataaaaaaaaaaaattccaatccaaagctcaatcaaatcaaca
tggcaacatccatgagagttaacaagggcaaaattaaataatatgaaaacattagaaaaa
tttagttttgagcataaatcacacattaaaaagaatttatttaaatcaaaagaggttatc
aacatcattaaaaactaagttttacattctttcatcacttgataataaggtcaaaactca
aaacaaacctttgtgtttatgtttttttatatcagttttcgattatatatgtttagtatg
ttcttcaattgcatccctttgatgaaaactagctttgtcccaaatgatcatgtaagatcc
aaactccaccacacaaaacattgaagttaatccaagaagattctacatttttaaatactt
ttcccaaggtctcttatatactgatttattctagtagtaattatttgatttctttttctt
ccaaatcatttagaaatttcacaatataaccaccaactttgtaagttgtaacataagtga
tagaaattagatcaaactcttgttaaaacttgataacgtcaagagcaatacgttgggcct
agtagtactcaagccccaaatatgggtagtgggtcattgaagacccaagttcaaaggaat
tttttttaacaaaagtaattaaataccctaatcatctcaaattcatgaaggaagccgccg
tatccaaaagctccttcacaatcaatctcagcatcgctgtgtcttaaaatcactcaatcg
aaattcggatttagatttgagcgaaagtcgcggcggtttttgtgattagggtttcgtaac
cgtgtccgccggtatatgag
