# Supplementary material for: Characteristics and Functions of MYB (v-Myb avivan myoblastsis virus oncogene homolog)-Related Genes in Arabidopsis thaliana
Source: Genes (Basel). 2023 Oct 31;14(11):2026. doi: 10.3390/genes14112026 (PMC10671209; doi:10.3390/genes14112026)
Supplement: Supplementary file 1 [file genes-14-02026-s001.zip › Supplementary legends.pdf]

Supplementary Figure 1 The logo of the conserved motifs in *AtMYB-related* genes.

Supplementary Figure 2. Phylogenetic tree, motif and gene structure of *AtMYB-related* genes. (A) NJ phylogenetic tree of 59 AtMYB proteins. (B) The motif composition of *AtMYB-related* protein. Different colored boxes represent different motifs. Detailed information of each motif is provided in Supplementary Figure 2. The x-axis represents the protein lengths. (C) Exon–intron structures of *AtMYB-related* genes. CDS, UTR and intron are represented by yellow, green and gray lines, respectively.

Supplementary Table 1 The sequence of the conserved motifs in *AtMYB-related* genes.

Supplementary Table 2 The overall information of *AtMYB-related* genes.

Supplementary Table 3 Cis-elements analysis of the *AtMYB-related* genes.
