# Supplementary material for: Characteristics and Functions of MYB (v-Myb avivan myoblastsis virus oncogene homolog)-Related Genes in Arabidopsis thaliana
Source: Genes (Basel). 2023 Oct 31;14(11):2026. doi: 10.3390/genes14112026 (PMC10671209; doi:10.3390/genes14112026)
Supplement: Supplementary file 1 [file genes-14-02026-s001.zip › Table S1-.pdf]

Motif	Sequences	E-value	Sites	Width
1	FVGTKTPTQVRSHAQKYFLKV	3.4e-255	22	21
2	TEEEHDLFLEALKLYGKGW	9.9e-262	51	19
3	QKSGTAIHIPPPRPKRKPAHPYPRKAPKN	1.3e-114	11	29
4	PDFAEVYNFIGSVFDPDTSGHLKKLKEMDPINFETVLLLMRNLSVNLSSP	1.7e-095	5	50
5	KVRKGRTITELKDKW	1.1e-095	42	15
6	PKQKWTTEEELALKAGVRKHGTGKWRSIL	7.8e-064	8	29
7	QETKDYKRIIRQHJDFETIRKKVEEGSYKSSRLNFYRDLLLLFTNARVFY	2.8e-038	4	50
8	GSDVSSIFLYIEERYEVPPBFKRLLSTRLKYLTAQGKLVKVKHKYRIQNN	6.8e-036	5	50
