# Supplementary material for: Characteristics and Functions of MYB (v-Myb avivan myoblastsis virus oncogene homolog)-Related Genes in Arabidopsis thaliana
Source: Genes (Basel). 2023 Oct 31;14(11):2026. doi: 10.3390/genes14112026 (PMC10671209; doi:10.3390/genes14112026)
Supplement: Supplementary file 1 [file genes-14-02026-s001.zip › Table S2-.pdf]

Gene ID	LOCUS	Gene Name	CDS/bp	Possible Function	Reference
AT1G01060	AT1G01060.1	LHY	1938	Regulation of circ adian rhythms	(Lu et al., 2009)
AT1G01380	AT1G01380.1	ETC1	252	ETC1, TRY, and CPC act in concer t to repress the tri chome cell fate in the shoot epider mis and the non-h air cell fate in the root epidermis	(Kirik et al., 2004a)
AT1G01520	AT1G01520.1	ASG4, RVE3	864	minor role in cloc k regulation	(Hsu et al., 2013)
AT1G06910	AT1G06910.1	TRFL7	1173	Binding to plant t elomere DNA	(Karamysheva et al., 2004)
AT1G07540	AT1G07540.1	TRFL2	1893	Binding to plant t elomere DNA	(Karamysheva et al., 2004)
AT1G09710	AT1G09710.1	DP1	1833	-	-
AT1G15720	AT1G15720.1	TRFL5	1173	Binding to plant t elomere DNA	(Karamysheva et al., 2004)
AT1G17460	AT1G17460.1	TRFL3	1815	Binding to plant t elomere DNA	(Karamysheva et al., 2004)
AT1G17520	AT1G17520.1	-	891	-	-
AT1G18330	AT1G18330.1	EPR1, RVE7	1119	Regulation of circ adian rhythms	(Kuno et al., 2003)
AT1G18960	AT1G18960.1	-	924	-	-
AT1G19000	AT1G19000.1	-	858	-	-
AT1G19510	AT1G19510.1	RL5, RSM4	303	Influence growth and development of Arabidopsis	(Baxter et al., 2007)
AT1G49950	AT1G49950.1	TRB1	903	Binds preferentiall y double-stranded telomeric repeats; Telomere DNA-bi nding AtTRB1 pr otein as a highly dynamic nuclear c omponent with fas t turnover in inter phase and tight ce	(Hwang et al., 2001) (Dvorackova et al., 2010)

				ll cycle-dependent regulation	
AT1G58220	AT1G58220.1	DRMY1	2505	Regulating cell ex pansion either dire ctly by affecting c ell wall architectur e and/or cytoplas mic growth or ind irectly through the ethylene and/or ABA signaling pat hways	(Wu et al., 2019)
AT1G70000	AT1G70000.1	MYBD	786	Enhances anthocya nin biosynthesis vi a repression of M YBL2	(Nguyen et al., 2015)
AT1G71030	AT1G71030.1	MYBL2	588	With GL3 togethe r, negatively regu late the expression of GL2 and supp ress the formation of trichomes; Ant hocyanin biosynth esis; Phytohormon e pathway	(Kirik et al., 2004b) (Zhao et al., 2021) (Castillo et al., 2018)
AT1G72650	AT1G72650.1	TRFL6	1875	Binding to plant t elomere DNA	(Karamysheva et al., 2004)
AT1G72740	AT1G72740.1	TRB5	870	-	-
AT1G74840	AT1G74840.1	-	798	-	-
AT1G75250	AT1G75250.1	RL6, RSM3	381	-	-
AT2G13960	AT2G13960.1	-	348	-	-
AT2G18328	AT2G18328.1	RL4	234	-	-
AT2G30420	AT2G30420.1	ETC2	339	Overexpression res ults in the suppres sion of trichomes and overproductio n of root hairs	(Kirik et al., 2004b)
AT2G30424	AT2G30424.1	TCL2	303	Involved in the ne gative regulation o f trichome formati on	(Gan et al., 2011)

AT2G30432	AT2G30432.1	TCL1	255	Negatively regulates trichome formation by suppressing GL1 (GLABRA 1)	(Wang et al., 2007)
AT2G36960	AT2G36960.1	TKI1	2235	-	-
AT2G42150	AT2G42150.1	-	1896	-	-
AT2G44430	AT2G44430.1	-	1941	-	-
AT2G46410	AT2G46410.1	CPC	285	Determination of hair or nonhair type cells, mediate lateral inhibition during trichome and root hair patterning; Over-expression results in increase of root hairs and decrease of trichome numbers; Regulation of anthocyanin biosynthesis and stomatal formation	(Wada et al., 1997) (Zhu et al., 2009) (Serna, 2008)
AT2G46830	AT2G46830.1	CCA1	1827	Regulation of circadian rhythms	(Wang et al., 1997)
AT3G09600	AT3G09600.1	RVE8	897	Regulation the biosynthesis of anthocyanins; Increase the acetylation level of histone H3 to promote the expression of histone H3	(Gray et al., 2017) (Farinas and Mas, 2011)
AT3G10113	AT3G10113.1	-	1898	-	-
AT3G10580	AT3G10580.1	-	915	-	-
AT3G10590	AT3G10590.1	-	621	-	-
AT3G16350	AT3G16350.1	NID1	1164	Increase ABA accumulation in low-nitrate conditions, promote plant growth	(Lee et al., 2020)

AT3G21430	AT3G21430.2	ALY3	3399	-	-
AT3G49850	AT3G49850.1	TRB3, TBP2	888	Telomere binding protein	(Schrumpfova et al., 2004)
AT3G53790	AT3G53790.1	TRFL4	1203	Binding to plant t elomere DNA	(Karamysheva et al., 2004)
AT3G57980	AT3G57980.1	-	1953	-	-
AT3G60110	AT3G60110.1	-	1926	-	-
AT4G01060	AT4G01060.1	CPL3, ETC3	234	Induction of root hair formation, co ntrol flower devel opment and epider mal cell size	(Tominaga et al., 2008)
AT4G01280	AT4G01280.1	RVE5	909	RVE3 and RVE5 promote clock pac e in conjunction with RVE4, RVE 6, and RVE8	(Gray et al., 2017)
AT4G09450	AT4G09450.1	-	603	-	-
AT4G36570	AT4G36570.1	RL3	177	-	
AT4G39160	AT4G39160.1	-	2260	-	-
AT4G39250	AT4G39250.1	RL1, RSM2	303	Influence growth and development of Arabidopsis	(Baxter et al., 2007)
AT5G02840	AT5G02840.1	LCL1, REV4	3332	Minor role in cloc k regulation	(Hsu et al., 2013)
AT5G17300	AT5G17300.1	RVE1	1164	Promoting primary dormancy of Ara bidopsis thaliana s eeds; Regulates hy pocotyl growth; n egative regulator o f freezing toleranc e	(Jiang et al. 2016) (Rawat et al., 2009) (Meissner et al., 2013)
AT5G37260	AT5G37260.1	CIR1, RVE2	864	Circadian clock an d seed germinatio n; positively regu-lates cold-responsi ve genes	(Zhang et al., 2007) (Guan et al. 2013)
AT5G41020	AT5G41020.1	-	1767	-	-
AT5G47390	AT5G47390.1	MYBH	1098	Hypocotyl elongati on; Cell expansion	(Huang et al., 2015) (Kwon et al., 2013)

				and leaf senescen ce	
AT5G52660	AT5G52660.1	RVE6	993	Minor role in cloc k regulation	(Hsu et al., 2013)
AT5G53200	AT5G53200.1	TRY	321	Mediate lateral in hibition during tri chome and root h air patterning in Arabidopsis; May be involved in sto matal formation w ith CPC	(Schellmann et al., 2002) (Serna, 2008)
AT5G56840	AT5G56840.1	-	702	-	-
AT5G58340	AT5G58340.1	-	1347	-	-
AT5G58900	AT5G58900.1	DIV1	867	-	-
AT5G61620	AT5G61620.1	-	954	-	-
AT5G67580	AT5G67580.1	TRB2, TBP3	900	Telomere binding protein	(Schrumpfova et al., 2004) (Lee et al., 2012)
