# Supplementary figures and images for: Characteristics and Functions of MYB (v-Myb avivan myoblastsis virus oncogene homolog)-Related Genes in Arabidopsis thaliana
Source: Genes (Basel). 2023 Oct 31;14(11):2026. doi: 10.3390/genes14112026 (PMC10671209; doi:10.3390/genes14112026)

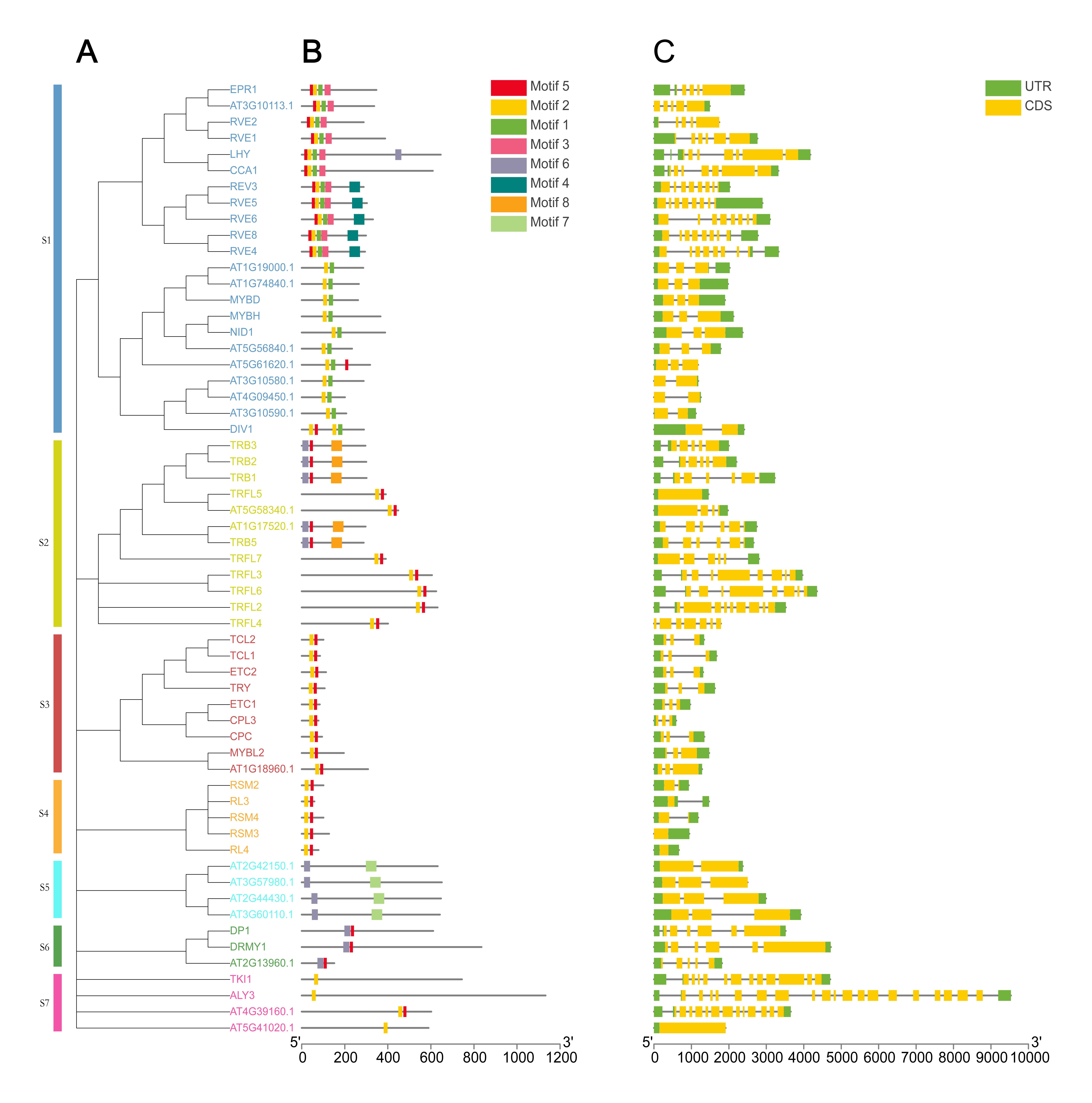

Supplement: Supplementary file 1 [file genes-14-02026-s001.zip › Figure S2 -.jpg]
